# High-Throughput Discovery of Synthetic Siderophores for Trojan Horse Antibiotics

**DOI:** 10.1021/acsinfecdis.4c00359

**Published:** 2024-10-22

**Authors:** Brent S. Weber, Nikki E. Ritchie, Simon Hilker, Derek C. K. Chan, Carsten Peukert, Julia P. Deisinger, Rowan Ives, Christine Årdal, Lori L. Burrows, Mark Brönstrup, Jakob Magolan, Tracy L. Raivio, Eric D. Brown

**Affiliations:** † Department of Biochemistry and Biomedical Sciences, McMaster University, Hamilton, Ontario L8S 4L8, Canada; ‡ Michael G. DeGroote Institute for Infectious Disease Research, McMaster University, Hamilton, Ontario L8S 4L8, Canada; § Department of Chemical Biology, Helmholtz Centre for Infection Research Inhoffenstraße 7, 38124 Braunschweig, Germany; ∥ Antimicrobial Resistance Centre, Norwegian Institute of Public Health, 0213 Oslo, Norway; ⊥ German Center for Infection Research (DZIF), Site Hannover-Braunschweig, Inhoffenstraße 7, 38124 Braunschweig, Germany; # Institute for Organic Chemistry (IOC), Leibniz Universität Hannover, Schneiderberg 1B, 30167 Hannover, Germany; ¶ Department of Biological Sciences, University of Alberta, Edmonton, Alberta T6G 2R3, Canada

**Keywords:** Trojan horse antibiotic, siderophore, siderophore-conjugate, antibiotic, high-throughput screening, iron

## Abstract

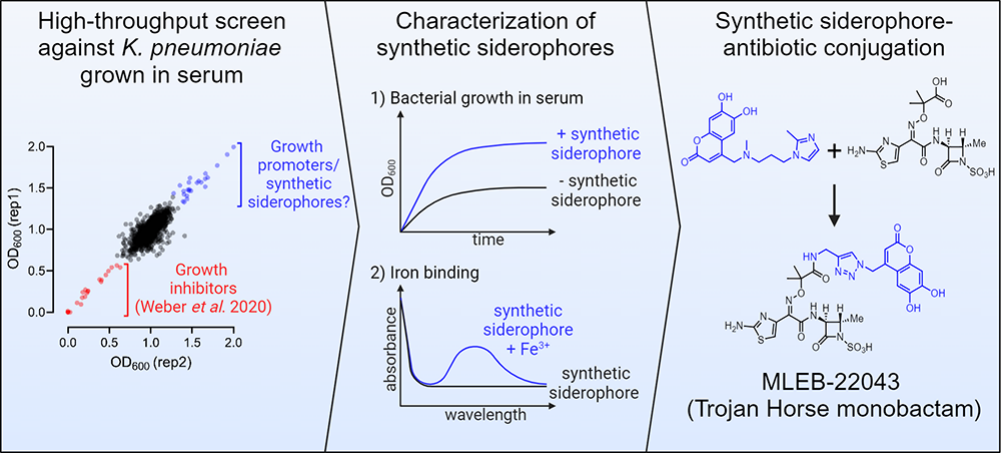

To cause infection, bacterial pathogens must overcome host immune factors and barriers to nutrient acquisition. Reproducing these aspects of host physiology in vitro has shown great promise for antibacterial drug discovery. When used as a bacterial growth medium, human serum replicates several aspects of the host environment, including innate immunity and iron limitation. We previously reported that a high-throughput chemical screen using serum as the growth medium enabled the discovery of novel growth inhibitors overlooked by conventional screens. Here, we report that a subset of compounds from this high-throughput serum screen display an unexpected growth enhancing phenotype and are enriched for synthetic siderophores. We selected 35 compounds of diverse chemical structure and quantified their ability to enhance bacterial growth in human serum. We show that many of these compounds chelate iron, suggesting they were acting as siderophores and providing iron to the bacteria. For two different pharmacophores represented among these synthetic siderophores, conjugation to the β-lactam antibiotic ampicillin imparted iron-dependent enhancement in antibacterial activity. Conjugation of the most potent growth-enhancing synthetic siderophore with the monobactam aztreonam produced MLEB-22043, a broad-spectrum antibiotic with significantly improved activity against *Klebsiella pneumoniae*, *Escherichia coli*, *Acinetobacter baumannii*, and *Pseudomonas aeruginosa*. This synthetic siderophore-monobactam conjugate uses multiple TonB-dependent transporters for uptake into *P. aeruginosa*. Like aztreonam, MLEB-22043 demonstrated activity against metallo-β-lactamase expressing bacteria, and, when combined with the β-lactamase inhibitor avibactam, was active against clinical strains coexpressing the NDM-1 metallo-β-lactamase and serine β-lactamases. Our work shows that human serum is an effective bacterial growth medium for the high-throughput discovery of synthetic siderophores, enabling the development of novel Trojan Horse antibiotics.

Antimicrobial resistance (AMR), responsible for an estimated 1.3 million deaths globally in 2019, is considered one of the top global public health and development threats by the United Nations, World Health Organization (WHO), and other global bodies.^1^ To guide innovators WHO has published a list of priority pathogens, where new antimicrobial agents are needed. The pathogens with “critical” or “high” need are the carbapenem-resistant Gram-negative bacteria, including *Acinetobacter baumannii*, *Pseudomonas aeruginosa*, and *Enterobacterales*.^2^ Thus, identifying new antibacterial drugs that target these pathogens has considerable global public health and patient benefit.^3^

The mammalian innate and adaptive immune systems, physical barriers, and nutrient limitation are formidable defenses against bacterial infections. In response, pathogenic bacteria encode metabolic, biosynthetic, and virulence pathways that facilitate evasion of these host defenses to promote infection.^4−6^ For example, in a process termed nutritional immunity, host proteins like transferrin sequester iron, making it inaccessible to invading microbes.^7^ Since iron is essential for bacterial growth, pathogens have evolved mechanisms to obtain this metal from the host, including secretion of high-affinity iron-binding molecules called siderophores.^7^ Siderophores can steal iron from host proteins and, in Gram-negative bacteria, are imported back across the bacterial outer membrane by TonB-dependent transporters (TBDTs). Some bacteria broaden their ability to acquire iron by producing multiple types of siderophores or by expressing TBDTs that recognize siderophores produced by other microorganisms (xenosiderophores) and siderophore-like compounds that have structural features of siderophores.^8−10^

Some microorganisms have exploited the promiscuity of bacterial siderophore uptake systems by producing sideromycins, a class of antibiotics comprising a siderophore moiety linked to an antibacterial compound.^11^ Sideromycins are imported across the bacterial outer membrane by endogenous siderophore transporters, resulting in the covert delivery of the antibiotic cargo. This is often referred to as a “Trojan Horse” mechanism, as the bacteria are effectively being “tricked” into taking up an antibiotic.^9,12^ This strategy has been co-opted by chemists to produce synthetic siderophore-antibiotic conjugates that are actively taken up by Gram-negative bacteria via siderophore transporters, resulting in increased compound accumulation. Although there have been numerous siderophore-antibiotic conjugates in development over the last 40 years, the first clinical approval for a Trojan Horse antibiotic was in 2019.^13,14^ That compound, called cefiderocol, incorporates an iron-chelating catechol moiety on a cephalosporin antibiotic scaffold, which imparts an iron-dependent enhancement in antibacterial activity and additional β-lactamase stability.^15,16^ Most Trojan Horse antibiotics that have advanced to late preclinical or clinical stages are β-lactams conjugated with a catechol, or its isostere hydroxypyridone, as the siderophore partner.^8,9,14,17−19^ While other siderophores and siderophore mimics have been tested, there has been relatively little investigation into the range of molecules that might function as synthetic siderophores.

Genome-wide mutagenesis and infection studies have shown that many bacterial genes required for infection are dispensable for growth in standard laboratory media.^20−24^ These conditionally essential processes, such as siderophore biosynthesis, are promising targets for new antibiotics, but inhibitors are difficult to find using conventional drug discovery techniques as they have no effect on in vitro growth.^25−27^ The development of bacterial growth media that mimic key aspects of host physiology has enabled the discovery of compounds that target these in vivo essential bacterial processes.^28−35^ We recently showed that human serum can be used as a physiologically relevant bacterial growth medium to identify antibacterial molecules that otherwise lack activity in conventional laboratory broth.^36^ Human serum retains important properties of host innate immunity and nutrient restriction, including sequestration of iron by transferrin.^22,30,37,38^ We developed a high-throughput screening platform using serum and found antibacterial compounds targeting pathways that were only essential in vivo, including iron acquisition.

While our previous work was aimed at finding compounds with antibacterial activity, a review of our high-throughput screening data revealed that many compounds promoted bacterial growth in serum. Here, we show that most of these growth promoters function as synthetic siderophores by providing iron to bacteria grown under the iron-limited conditions found in serum, but have no effect in iron-rich conventional broth. We then leverage these findings to synthesize synthetic siderophore-antibiotic drug conjugates and show that they are iron-dependent antibiotics imported via siderophore uptake pathways. One of these conjugates, the siderophore-monobactam MLEB-22043, demonstrates broad spectrum activity against multidrug resistant Gram-negative clinical isolates. Our work offers a platform for the high-throughput discovery of siderophores for Trojan Horse antibiotics, and further emphasizes the advantages of using physiologically relevant conditions for antibacterial drug discovery.

## Results

### Reanalysis of a High-Throughput Serum Screen Identifies Synthetic Siderophore Compounds with Growth-Enhancing Activity

**Figure 1:**
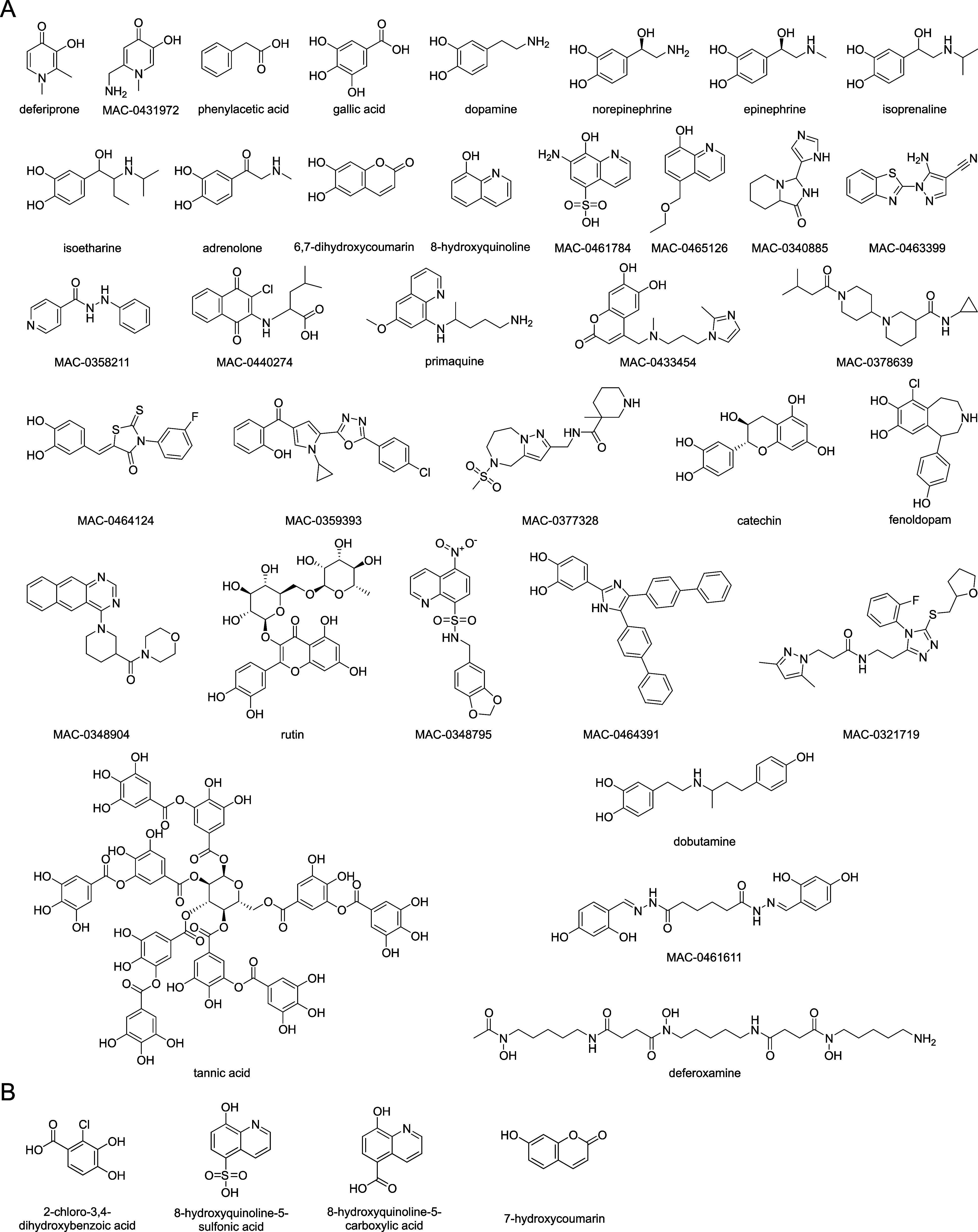
Putative growth-promoting compounds selected for follow-up studies. (A) Structures of putative growth-promoting compounds identified in the high-throughput serum screen and selected for further characterization. (B) Structures of additional control compounds used in follow-up experiments.

We previously screened ∼170,000 compounds from four small molecule libraries, including several thousand FDA-approved or bioactive compounds, for growth inhibitory activity against *Klebsiella pneumoniae* MKP103 grown in human serum.^36^ In that work, we monitored bacterial growth using absorbance measurements and identified several compounds that, based on low or minimal increases in absorbance readings, had antibacterial activity in serum. Recently, we noticed that some molecules in these screens seemed to enhance, rather than inhibit, *K. pneumoniae* MKP103 growth in serum, as evidenced by an unexpected increase in growth (Figure S1). In total, we identified 264 compounds from our primary small molecule screen that showed this apparent growth enhancement of *K. pneumoniae* in serum (Table S1). We found that 163 of these hit compounds were either iron chelators or possessed siderophore-like structural features, including catecholates, hydroxamates, phenolates and carboxylates (Table S1).^39^*K. pneumoniae* MKP103 produces the high affinity iron-chelating catecholate siderophore enterobactin, which is essential for serum growth,^36^ and *Klebsiella* spp. express numerous TBDTs that can uptake its own siderophores as well as exogenous xenosiderophores.^40,41^ We therefore hypothesized that these identified compounds could function as xenosiderophores or siderophore-like synthetic siderophores, providing iron to the bacteria in the otherwise iron-restricted environment of serum.^42^ To test this, we selected 35 hit compounds based on observed potency in the primary screen, structural diversity, and commercial availability, for follow-up studies (Figure 1A and Table 1), as well as additional control molecules (Figure 1B).

**Table 1:** Summary Data for Putative Growth-Promoting Compounds and Controls

#	compound ID^a^	compound name^b^	note^c^	growth promotion^d^	iron chelation^e^
1	MAC-0647634	adrenalone	screening hit	yes	yes
2	MAC-0645049	isoprenaline	screening hit	yes	yes
3	MAC-0645012	isoetharine	screening hit	yes	yes
4	MAC-0644910	fenoldopam	screening hit	yes	yes
5	MAC-0647974	deferiprone	screening hit	yes	yes
6	MAC-0644937	dopamine	screening hit	yes	yes
7	MAC-0646093	epinephrine	screening hit	yes	yes
8	MAC-0646158	tannic acid	screening hit	yes	yes
9	MAC-0646158	norepinephrine	screening hit	yes	yes
10	MAC-0644125	catechin	screening hit	yes	yes
11	MAC-0646163	gallic acid	screening hit	yes	yes
12	MAC-0647892	phenylacetate	screening hit	no	no
13	MAC-0646049	deferoxamine	screening hit	yes	yes
14	MAC-0646815	rutin	screening hit	yes	yes
15	MAC-0644737	dobutamine	screening hit	yes	yes
16	MAC-0646251	primaquine	screening hit	no	no
17	MAC-0646242	8-hydroxyquinoline	screening hit	yes	yes
18	MAC-0646112	6,7-dihydroxycoumarin	screening hit	yes	yes
19	MAC-0461784	N/A	screening hit	yes	yes
20	MAC-0464124	N/A	screening hit	yes	yes
21	MAC-0461611	N/A	screening hit	yes	yes
22	MAC-0464391	N/A	screening hit	yes	yes
23	MAC-0465126	N/A	screening hit	yes	yes
24	MAC-0463399	N/A	screening hit	yes	no
25	MAC-0340885	N/A	screening hit	no	no
26	MAC-0359393	N/A	screening hit	yes	no
27	MAC-0348904	N/A	screening hit	yes	no
28	MAC-0348795	N/A	screening hit	no	no
29	MAC-0358211	N/A	screening hit	no	yes
30	MAC-0321719	N/A	screening hit	no	no
31	MAC-0440274	N/A	screening hit	yes	no
32	MAC-0378639	N/A	screening hit	no	no
33	MAC-0377328	N/A	screening hit	yes	no
34	MAC-0433454	N/A	screening hit	yes	yes
35	MAC-0431972	N/A	screening hit	yes	yes
36	N/A	iron(III) chloride, FeCl_3_	control compound	yes	N/A
37	N/A	8-hydroxyquinoline-5-sulfonic acid	control compound	yes	yes
38	N/A	8-hydroxyquinoline-5-carboxylic acid	control compound	yes	yes
39	N/A	7-hydroxycoumarin	control compound	no	no
40	N/A	2-chloro-3,4-dihydroxybenzoic acid	control compound	yes	yes
41	N/A	SH-263	conjugate	N/A	yes
42	N/A	SH-267	conjugate	N/A	yes
43	N/A	ampicillin	control compound	N/A	no
44	N/A	cefiderocol	control compound	N/A	yes
45	N/A	MLEB-22043	conjugate	N/A	yes
46	N/A	aztreonam	control compound	N/A	no
47	N/A	MB-1	control compound	N/A	yes

**Figure 2:**
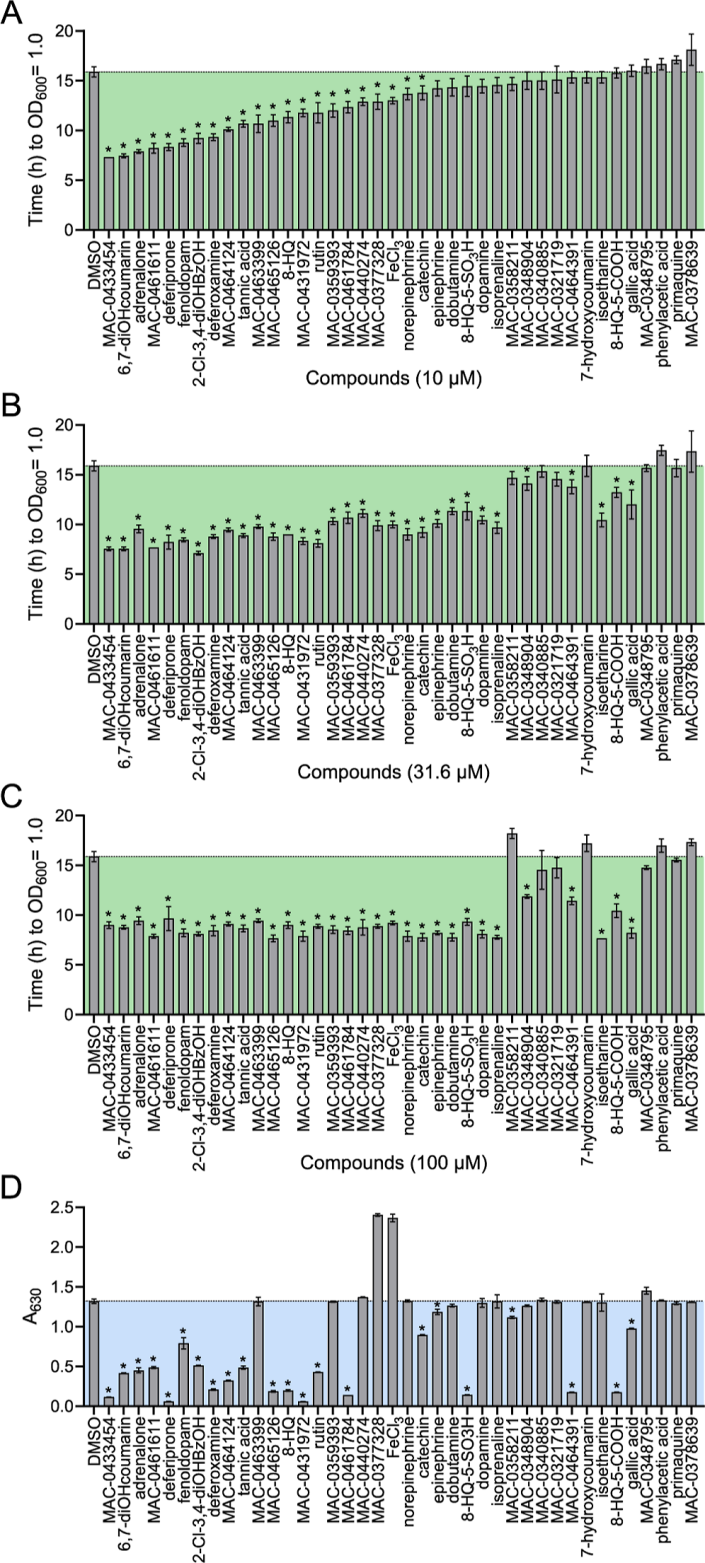
Quantification of compound growth enhancement and iron-binding identifies potent synthetic siderophores. Compounds were characterized for growth promotion of *K. pneumoniae* MKP103 in 50% serum at (A) 10, (B) 31.6, and (C) 100 μM. Growth enhancement was determined from growth curves as the time it took the cultures to reach an OD_600_ nm of 1.0; the shorter the time, the more potent the growth promoter. For the 10 μM concentration, compounds were rank-ordered from shortest (more potent) to longest (less potent) growth times. This same order is maintained for the 31.6 and 100 μM concentrations. The dashed line spanning the graph indicates the value of the DMSO control. * indicates significant difference (*p* < 0.05) from the DMSO control as determined by one-way ANOVA and Dunnet’s multiple comparisons test. The experiment was done in triplicate and graphed data shows the mean ± standard deviation. (D) CAS assay data. Each compound was tested at a final concentration of 0.5 mM in the presence of the CAS reagent. A reduction in absorbance compared to the DMSO control is considered positive for iron-binding. Compound order in the graph is maintained as in Figure 2A. The dashed line spanning the graph indicates the value of the DMSO control. * indicates significant difference (*p* < 0.05) from the DMSO control as determined by one-way ANOVA and Dunnet’s multiple comparisons test. The experiment was performed in triplicate, and the graphed data show the mean ± standard deviation. Abbreviations for compound names: 6,7-dihydroxycoumarin: 6,7-diOHcoumarin; 2-chloro-3,4-dihydroxybenzoic acid: 2-Cl-3,4-diOHBzOH; 8-hydroxyquinoline: 8-HQ; 8-hydroxyquinoline-5-sulfonic acid: 8-HQ-5-SO3H; 8-hydroxyquinoline-5-carboxylic acid: 8-HQ-5-COOH.

a Unique compound identifier; N/A: not applicable.

b Alternative name; N/A: not applicable.

c Screening hits are compounds identified from high-throughput screen. Conjugate indicates synthetic siderophore-antibiotic compounds made for this work.

d Growth promotion defined as a significant decrease in time for *K. pneumoniae* to reach *A*_600_ = 1.0 in the presence of 100 μM compound compared to the DMSO control during growth in serum. See Figure 2. N/A: not applicable.

e Iron chelation is defined as compounds which were active in the Chrome Azurol S (CAS) assay and/or the FeCl_3_ colorimetric binding assay. N/A: not applicable.

The compounds were tested for their capacity to enhance *K. pneumoniae* MKP103 growth in serum and conventional cation-adjusted Mueller–Hinton broth (MHB) growth medium. We reasoned that this would allow us to distinguish whether these compounds specifically enhanced growth in serum or more broadly affected growth. Bacterial growth in serum or MHB was monitored kinetically in the presence of 10, 31.6, or 100 μM of each compound. We found no significant growth enhancement in MHB for any compound tested (Figure S2). Conversely, many compounds had substantial growth promoting effects on serum-grown *K. pneumoniae* MKP103 (Figure S3). Growth enhancement in serum was quantified as the time it took for the cultures to reach an OD_600_ of 1.0, and we found that 28 of the 35 compounds tested showed significant growth promotion compared to the DMSO control at 100 μM or lower (Figure 2A–C).

The most potent growth promoter at 10 μM was MAC-0433454, a dihydroxycoumarin, which reduced the time to reach an OD_600_ of 1.0 from roughly 16 h for the DMSO control to approximately 7.5 h (Figure 2A). In agreement with this, the second most potent compound at this concentration was the structural analog 6,7-dihyroxycoumarin. The control compound 7-hydroxycoumarin showed no growth promoting activity in this assay, indicating the importance of the dihydroxycoumarin pharmacophore for growth promotion. In several instances we identified multiple structural analogs as growth promoters, for example 8-hydroxyquinoline and analogs MAC-0461784 and MAC-0465126. We also tested sulfonic and carboxylic acid derivatives of the 8-hydroxyquinoline scaffold as controls (Figure 1B), and these were similarly active. Other compounds of note were deferiprone, a chelator related to the siderophore scaffold in several published siderophore-antibiotic conjugates,^43−47^ and an analog of deferiprone, MAC-0431972. Both compounds showed potent growth promotion at 10 μM and higher concentrations. As a positive control, we tested 2-chloro-3,4-dihydroxybenzoic acid, the synthetic siderophore used in the siderophore-antibiotic cefiderocol.^48^ As expected, this compound promoted growth of *K. pneumoniae* MKP103 at all concentrations. Additionally, supplementation of serum with exogenous iron in the form of FeCl_3_ resulted in a dose-dependent growth enhancement, although many compounds showed greater potency on an equimolar basis, particularly at the 10 μM concentration.

In parallel, these compounds were tested for their ability to chelate iron using the CAS assay and UV–vis spectroscopy.^49^ In the CAS assay, the ability of a compound to chelate iron from the iron-loaded CAS dye can be detected as a reduction in absorbance.^50,51^ Nineteen of the 35 hit compounds showed a significant reduction in absorbance in the CAS assay, indicative of iron sequestration from the CAS dye due to a higher affinity for iron than CAS (Figure 2D). Next, we tested for shifts in the absorption spectra of our compounds when mixed with an equimolar amount of FeCl_3_. Compound complexation with metals often results in a color change that can be measured as a shift in the UV–vis absorbance spectrum.^52^ A total of 23 compounds showed a detectable shift in their UV–vis absorbance spectra upon addition of iron, including 18 of the 19 compounds that were active in the CAS assay (Figure S4). Furthermore, of the 28 compounds that showed growth-promoting activity, 23 were active in at least one of these iron-binding assays, suggesting that their mechanism of growth promotion involves providing iron to the bacterial cell. The remaining 5 compounds (MAC-0463399, MAC-0359393, MAC-0348904, MAC-0440274, MAC-0377328) therefore may have a different mechanism of growth promotion. Interestingly, MAC-0377328 showed a unique phenotype in the CAS assay, where the compound caused an increase in absorbance when it was added to the CAS dye, similar in effect to the FeCl_3_ control (Figure 2D). Together, these data suggest that our initial screening effort identified molecules with growth promoting activity and that most compounds act as synthetic iron-binding siderophores, delivering iron to the bacterial cell for uptake via TBDTs (Tables 1 and S2).

### Azide-functionalized Synthetic Siderophores Transport Iron into *E. coli* and *P. aeruginosa*

**Figure 3:**
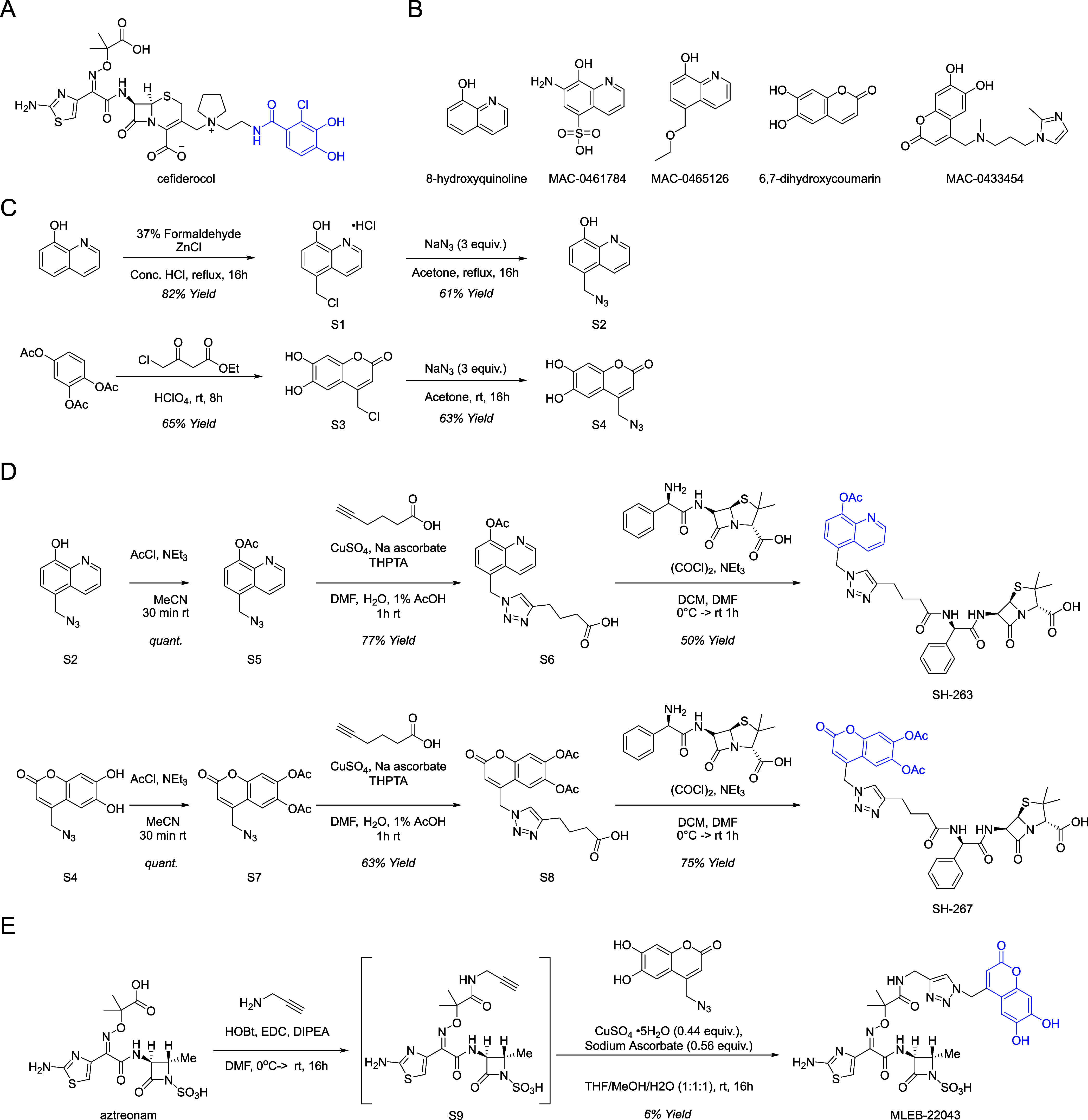
Structures of synthetic siderophores and synthesis of Trojan Horse antibiotics. (A) Structure of the FDA-approved Trojan Horse antibiotic cefiderocol. The siderophore component of cefiderocol is highlighted in blue. (B) Structures of screening hit compounds containing the 8-hydroxyquinoline and 6,7-dihydroxycoumarin pharmacophores that were active in our growth promotion and iron-binding assays. (C) Synthesis of click-enabled siderophore azides. (D) Synthesis of ampicillin siderophore conjugate prodrugs SH-263 and SH-267. The siderophore moiety of the conjugates is highlighted in blue. (E) Synthesis of aztreonam siderophore conjugate MLEB-22043. The siderophore moiety of the conjugate is highlighted in blue.

Several of the compounds identified by our screen resembled the iron-binding catechol moiety of cefiderocol (Figure 3A). Thus, we reasoned that the growth-promoting and iron-chelating molecules we characterized may represent attractive synthetic siderophore partners for a Trojan Horse antibiotic conjugate strategy. We chose the 8-hydroxyquinoline and 6,7-dihydroxycoumarin pharmacophores as proof-of-concept compounds, as both were represented multiple times in our hit compound list (Figure 3B and Table S1). MAC-0433454 and 6,7-dihydroxycoumarin were the most potent compounds in our growth promotion assay, and the hydroxyquinoline compounds were nearly as potent as the cefiderocol siderophore moiety 2-chloro-3,4-dihydroxybenzoic acid, particularly at the higher concentrations tested (Figure 2A–C). 2-chloro-3,4-dihydroxybenzoic acid, 8-hydroxyquinoline, MAC-0461784, MAC-0465126, MAC-0433454 and 6,7-dihydroxycoumarin all showed iron-binding activity in the CAS assay, whereas the control molecule 7-hydroxycoumarin showed no activity (Figure 2D).

**Figure 4:**
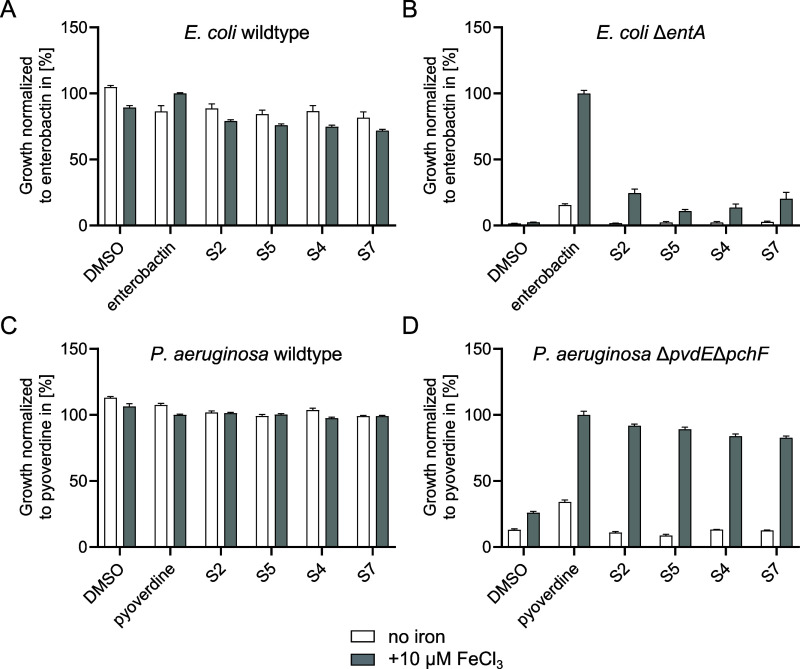
Growth recovery assay in siderophore-deficient *E. coli* and*P. aeruginosa* mutants with modified synthetic siderophores. Growth recovery of (A) wildtype *E. coli* BW25113, (B) enterobactin-deficient *E. coli* BW25113 Δ*entA*, (C) wildtype *P. aeruginosa* PAO1, and (D) pyoverdine/pyochelin deficient *P. aeruginosa* PAO1 Δ*pvdE*Δ*pchF.* Bacteria were grown in low-iron LMR medium in the absence (white bars) or presence (gray bars) of 10 μM FeCl_3_. The indicated compounds were added at 10 μM, with DMSO as the solvent control. The percent growth relative to enterobactin (A,B) or pyoverdine (C,D) is plotted; *n* = 3, error bars correspond to ± standard error of mean. The structures of S2, S5, S4, and S7 are shown in Figure 3.

We synthesized azido-8-hydroxyquinoline (**S2**) and azido-6,7-dihydroxycoumarin (**S4**) to enable a click chemistry-based functionalization of antibiotics with these siderophores (Figure 3C). Previous studies have shown that the use of acetylated siderophore components facilitates chemical synthesis while simultaneously preventing enzymatic deactivation by *O-*methyltransferases.^53,54^ The acetylated siderophores serve as prodrugs and are readily deacetylated in bacterial growth media.^55^ Therefore, we also synthesized the azido-acetyl-8-hydroxyquinoline (**S5**) and azido-acetyl-6,7-dihydroxycoumarin intermediates (**S7**) (Figure 3D). To determine whether the modified synthetic siderophores were still functional, we measured compound-mediated delivery of iron using the previously described bacterial growth recovery assay.^55,56^ In contrast to their wildtype parent, mutant strains of *Escherichia coli* BW25113 (Δ*entA*) or *P. aeruginosa* PAO1 (Δ*pvdE*Δ*pchF*) which cannot biosynthesize their endogenous siderophores enterobactin or pyoverdine and pyochelin, respectively, were unable to grow significantly in low iron LMR minimal medium with or without supplementation of 10 μM Fe^3+^, as expected (Figure 4A–D).^55,56^ The exogenous addition of their natural siderophores enterobactin or pyoverdine at a concentration of 10 μM restored the growth of the *E. coli* and *P. aeruginosa* mutants in Fe^3+^ supplemented media (Figure 4B,D). Notably, the addition of 8-hydroxyquinoline derivatives (**S2** and **S5**) and 6,7-dihydroxycoumarin derivatives (**S4** and **S7**) at 10 μM rescued the growth for both mutant strains in Fe^3+^ supplemented media (Figure 4B,D). While the growth recovery was weaker compared to enterobactin in *E. coli* BW25113 Δ*entA*, it was almost as efficient as pyoverdine in *P. aeruginosa* PAO1 Δ*pvdE*Δ*pchF*. In sum, the azido- and acetyl-modifications were well-tolerated with respect to the siderophore function, and both intermediates were found suitable for antibiotic conjugation.

### Ampicillin-Siderophore Conjugates SH-263 and SH-267 Show Iron-dependent Activity

To test whether the 8-hydroxyquinoline and 6,7-dihydroxycoumarin pharmacophores could serve as synthetic siderophores for Trojan Horse antibiotics, we next synthesized conjugates with the β-lactam antibiotic ampicillin. Efforts to couple an alkyne-functionalized ampicillin via a copper-catalyzed alkyne–azide-cycloaddition (CuAAC) to the azide-functionalized siderophore mimics failed. Instead, 6-hexynoic acid was attached to the siderophores via CuAAC, activated as an acyl chloride and subsequently coupled to the free amino function of ampicillin, generating the 8-hydroxyquinoline conjugate SH-263 and the 6,7-dihydroxycoumarin conjugate SH-267 (Figure 3D). Like cefiderocol, both antibiotic conjugates could chelate iron, whereas ampicillin lacked detectable iron-binding activity (Figure S5A–C). SH-263 and SH-267 were tested for their minimum inhibitory concentrations (MICs) against several Gram-negative bacteria under standard (MHB), iron-depleted (ID-MHB) and iron-repleted (ID-MHB plus 100 μM FeCl_3_) growth conditions, with cefiderocol serving as a control. Clinically, cefiderocol MICs are determined in ID-MHB, where MICs are typically several doubling dilutions lower than in standard MHB.^57,58^ Against *K. pneumoniae* MKP103, SH-263, SH-267, and ampicillin were inactive in all three media types (Table 2). As expected, cefiderocol showed an 8-fold decreased MIC in ID-MHB compared to MHB, and its MIC was increased in ID-MHB supplemented with FeCl_3_. Given the resistance phenotype of *K. pneumoniae* MKP103, mediated at least in part by β-lactamases,^59,60^ we next tested the drug sensitive strains *E. coli* BW25113 and *K. pneumoniae* ATCC 43816. Against these strains, SH-263 and SH-267 showed ≥8-fold decreased MICs in ID-MHB compared to MHB, whereas ampicillin activity showed no improvement against either strain. The enhanced activity of SH-263 and SH-267 in ID-MHB was lost upon addition of excess iron, suggesting an iron-dependent mechanism of enhancement. SH-267 also showed activity against *P. aeruginosa* PAO1, which was modestly enhanced in ID-MHB, despite this strain being highly resistant to ampicillin. Thus, SH-263 and SH-267 show activity consistent with iron-dependent Trojan Horse antibiotics.

**Table 2:** MICs of SH-263, SH-267, Ampicillin, and Cefiderocol against *K. pneumoniae*, *E. coli*, and *P. aeruginosa*

	K. pneumoniae MKP103			E. coli BW25113			K. pneumoniae ATCC 43816			P. aeruginosa PAO1		
	MIC (μg/mL)			MIC (μg/mL)			MIC (μg/mL)			MIC (μg/mL)		
compound	MHB^a^	ID^b^	ID + FeCl_3_^c^	MHB^a^	ID^b^	ID + FeCl_3_^c^	MHB^a^	ID^b^	ID + FeCl_3_^c^	MHB^a^	ID^b^	ID + FeCl_3_^c^
SH-263	>32	>32	>32	32	4	>32	>32	8	>32	>32	>32	>32
SH-267	>32	>32	>32	16	2	>32	16	2	>32	16	8	>32
ampicillin	>32	>32	>32	8	8	8	16	32	16	>32	>32	>32
cefiderocol	0.5	0.06	2	0.06	0.008	0.12	0.03	0.016	0.25	0.5	0.06	>2

a MHB = Cation-adjusted MHB.

b ID = Iron-depleted MHB.

c ID + FeCl_3_ = ID-MHB plus 100 μM FeCl_3_.

### MLEB-22043 is a Potent Siderophore-Monobactam Antibiotic

Although SH-263 and SH-267 both showed iron-dependent enhancement of activity, the usefulness of an ampicillin conjugate may be limited against multidrug-resistant Gram-negative bacteria. Indeed, the most clinically advanced siderophore conjugates have typically used monobactams or cephalosporins as the partner antibiotic.^8,14,61^ We therefore synthesized a conjugate of aztreonam, a clinically used monobactam, with siderophore 6,7-dihydroxycoumarin, which showed the most broad-spectrum activity as siderophore partner in SH-267. The carboxylic acid of aztreonam was functionalized as an *N*-propargylamide, and treatment of this alkyne with our azido-6,7-dihydroxycoumarin under CuAAC conditions yielded MLEB-22043 (Figure 3E).

MLEB-22043 had iron-binding activity in both the CAS and UV–vis absorption assays, which was not seen for aztreonam alone (Figure S5B,C). As with SH-263 and SH-267, tests of MLEB-22043 activity against *K. pneumoniae*, *E. coli*, and *P. aeruginosa* were performed under standard, iron-depleted, and iron-repleted conditions. MLEB-22043 was active against all strains and exhibited a significant iron-dependent enhancement in potency (Table 3). Of note, MLEB-22043 showed markedly improved activity compared to aztreonam against *P. aeruginosa* PAO1, with a > 100-fold increased potency in ID-MHB.

**Table 3:** MICs of MLEB-22043 and Aztreonam against *K. pneumoniae*, *E. coli*, and *P. aeruginosa*

	K. pneumoniae MKP103			E. coli BW25113			K. pneumoniae ATCC 43816			P. aeruginosa PAO1		
	MIC (μg/mL)			MIC (μg/mL)			MIC (μg/mL)			MIC (μg/mL)		
compound	MHB^a^	ID^b^	ID + FeCl_3_^c^	MHB^a^	ID^b^	ID + FeCl_3_^c^	MHB^a^	ID^b^	ID + FeCl_3_^c^	MHB^a^	ID^b^	ID + FeCl_3_^c^
MLEB-22043	8	0.5	16	0.5	0.12	1	0.5	0.12	2	1	0.06	4
aztreonam	0.5	0.5	0.5	0.12	0.12	0.12	0.06	0.06	0.06	8	4	8

a MHB = cation-adjusted MHB.

b ID = iron-depleted MHB (ID-MHB).

c ID + FeCl_3_ = ID-MHB plus 100 μM FeCl_3_.

The potency of MLEB-22043 against *P. aeruginosa*, a species considered to have a relatively impermeable outer membrane,^62,63^ encouraged us to determine its MIC against a broader panel of 97 isolates of *P. aeruginosa* (95 clinical isolates and 2 laboratory strains). In agreement with previous data,^64^ the MIC_90_ for aztreonam was >16 μg/mL, above the clinical breakpoint of ≤8 μg/mL (Table 4). In contrast, the MIC_90_ for MLEB-22043 was 0.5 μg/mL, suggesting it has potent antibacterial activity against diverse clinical isolates of *P. aeruginosa*.

**Table 4:** MIC Cumulative Frequency Distribution for MLEB-22043 and Aztreonam Tested against *P. aeruginosa* Isolates (*n* = 97)

	cumulative % frequency distribution by MIC (μg/mL)^a^											
compound	<0.03	0.03	0.06	0.12	0.25	0.5	1	2	4	8	16	>16
MLEB-22043^b^	23.7	23.7	47.4	75.3	83.5	**92.8**	99.0	99.0	100.0	100.0	100.0	100.0
aztreonam^c^	0.0	0.0	0.0	0.0	1.0	2.1	2.1	3.1	29.9	73.2	88.7	**100.0**

a Bold values indicate MIC_90_ value for each MIC distribution.

b Tested in iron-depleted MHB.

c Tested in MHB.

### Uptake of MLEB-22043 by *P. aeruginosa*

Siderophore-antibiotic conjugates exploit TBDTs for their uptake into Gram-negative bacteria. Indeed, a *tonB* mutant of *E. coli* showed 32-fold increased resistance to MLEB-22043 and cefiderocol (Table S3). Multiple TBDTs can mediate uptake of a given conjugate, and *P. aeruginosa* is a model pathogen to study routes of conjugate uptake, particularly given its large number of TBDTs compared to *E. coli*.^65−67^ To determine which *P. aeruginosa* TBDTs mediate MLEB-22043 uptake, we cloned 35 predicted TBDTs under the control of an arabinose-inducible promoter and introduced each individually into wild-type *P. aeruginosa* PA14. Each recombinant strain was then tested for susceptibility to MLEB-22043, aztreonam, and cefiderocol in MHB containing 1% arabinose. Of the 29 TBDTs that were amenable to overexpression, PiuA, PirA and PfuA overexpression resulted in increased susceptibility to both MLEB-22043 and cefiderocol, but no change in the aztreonam MIC (Table S4). Analysis of MICs against TBDT null strains grown in MHB showed that deletion of *piuA* resulted in a 4- to 8-fold increase in MIC to MLEB-22043 and cefiderocol, whereas deletion of *pirA* or *pfuA* had modest or no effect (Table S5). Consistent with previous studies, deletion of both *piuA* and *pirA* resulted in the highest MICs for MLEB-22043 and cefiderocol.^65^ The elevated MICs in the double mutant were restored to wild-type levels by overexpression of *piuA*. While overexpression of *pirA* similarly reduced the MIC for MLEB-22043 in the double mutant, its susceptibility to cefiderocol was not fully restored.

Next, we tested previously isolated spontaneous and targeted *P. aeruginosa* PAO1 mutants that showed in vitro resistance to the preclinical siderophore-monobactam conjugates SMC-3176 and MB-1 (Figure S5A).^44^ That previous work showed that the spontaneously resistant isolates had mutations in genes associated with iron acquisition (Table 5). Strain ARC4736 has a large deletion in the *piu* iron-uptake locus, while ARC6046 has a mutation in the promoter region of the transcriptional regulator *fecI*, resulting in increased expression of the ferric citrate siderophore transport pathway. These strains showed a 4-fold and >8-fold increased MIC, respectively, to MLEB-22043 in MHB. Strains with targeted deletions in *piuA* or *piuC* displayed 4-fold increased resistance to MLEB-22043, whereas deletion of the TBDT *pirA* had no effect. As with PA14, deletion of both *piuA* and *pirA* led to >8-fold increased MIC. Interestingly, when the same strains were tested in ID-MHB, we saw at most a 2-fold change in MIC, with the exception being the double *piuA pirA* deletion mutant, where MICs rose 16-fold. A similar MIC pattern was seen for the control siderophore conjugates MB-1 and cefiderocol, with significantly lower magnitude MIC shifts seen against mutants grown in ID-MHB; indeed, cefiderocol MICs in ID-MHB remained below the *P. aeruginosa* breakpoint of ≤4 μg/mL, as seen previously.^65^ The MICs of MLEB-22043 and aztreonam against *P. aeruginosa* PA14 mutants lacking *tonB1, tonB2,* or *tonB3* were similar to the wild-type strain (Table S6). Additionally, while we found that a *P. aeruginosa* strain lacking the outer membrane components of the major efflux systems was 8-fold more susceptible to aztreonam compared to the parent strain, the activity of MLEB-22043 was unaffected (Table S7), suggesting that MLEB-22043 overcomes the efflux liabilities of aztreonam.^68−70^ Together, these data suggest that, while PiuA and PirA are major transporters of MLEB-22043 and other siderophore-conjugates, there are additional routes of entry for these compounds, especially under iron-limited conditions.

**Table 5:** MICs of MLEB-22043, MB-1, Cefiderocol, and Aztreonam against *P. aeruginosa* PAO1 Mutants

		MIC, μg/mL^a^						
		MLEB-22043		MB-1		cefiderocol		aztreonam
PAO1 strain	mutation	MHB	ID-MHB^a^	MHB	ID-MHB^a^	MHB	ID-MHB^a^	MHB
ARC545	wildtype	2	0.12	0.5	0.12	1	0.06	8
ARC4736	Δ[PiuC(PA4515)···PA4521]	**8**	0.25	**8**	**0.5**	**8**	**0.25**	8
ARC6046	p*fecI* (−44 T → C)	**>16**	0.25	**16**	0.12	**16**	0.12	4
ARC4238	Δ*piuA*	**8**	0.12	**8**	**0.5**	**8**	**0.25**	8
ARC5406	Δ*piuC*	**8**	0.25	**16**	**0.5**	**8**	**0.25**	8
ARC4239	Δ*pirA*	2	0.12	0.5	0.12	1	0.06	8
ARC4242	Δ*piuApirA*	**>16**	**2**	**>16**	**2**	**16**	**0.5**	8

a Bold values indicate ≥4-fold increase in MIC from the wildtype strain in the indicated growth conditions.

### MLEB-22043 Retains Activity in the Presence of Metallo-β-Lactamases

Monobactam antibiotics are distinct from other β-lactams in that they are active against bacteria expressing class B metallo-β-lactamases, including worrisome enzymes like NDM-1.^71^ However, aztreonam remains susceptible to inactivation by serine β-lactamases. To determine whether MLEB-22043 had a β-lactamase susceptibility profile similar to aztreonam, we compared their activity in ID-MHB against hyperpermeable *E. coli* Δ*bamB*Δ*tolC* strains expressing various β-lactamases from a plasmid.^72,73^ As expected, aztreonam MICs were unchanged against *E. coli* expressing the metallo-β-lactamases NDM-1, VIM-1, and IMP-1 compared to the vector control (Table 6). In contrast, expression of the serine β-lactamases CTX-M-55, CMY-42, or SHV-1 resulted in significantly increased aztreonam MICs. When combined with the broad-spectrum serine β-lactamase inhibitor avibactam, aztreonam MICs against CTX-M-55 and SHV-1 overexpressing strains returned to vector control levels. Avibactam also improved aztreonam activity against CMY-42, but MICs remained 8-fold higher than the vector control. We found an identical profile for MLEB-22043; while metallo-β-lactamases had no effect on activity, serine β-lactamase expression resulted in increased MICs. Avibactam restored MLEB-22043 activity against serine β-lactamase producers, including CMY-42. We also tested ceftazidime and cefiderocol, with and without avibactam, as control compounds. Compared to the vector control strain, ceftazidime MICs increased against all β-lactamase-expressing strains. As expected, avibactam improved ceftazidime activity against strains expressing serine, but not metallo-, β-lactamases. Cefiderocol, which has enhanced stability against many β-lactamases, was less active against strains overexpressing NDM-1, VIM-1, CMY-42, and SHV-1, although the magnitude of activity loss was less pronounced than for ceftazidime. Avibactam restored cefiderocol activity against the serine β-lactamases CMY-42 and SHV-1 but did not improve MICs against NDM-1 or VIM-1. Taken together, this data shows that MLEB-22043 is not affected by metallo-β-lactamases and that its susceptibility to serine β-lactamases can be overcome with avibactam.

**Table 6:** MICs of MLEB-22043 and Antibiotic Comparators against *E. coli* Δ*bamB*Δ*tolC* Strains Overexpressing β-Lactamase Enzymes

		MIC, μg/mL^a^							
plasmid	β-lactamase overexpressed	ATM	ATM + AVI	043	043 + AVI	CAZ	CAZ + AVI	CFDC	CFDC + AVI
pGDP1		0.016	0.016	0.03	0.03	0.03	0.03	0.008	0.008
pGDP1:*ndm-1*	NDM-1	0.016	0.016	0.03	0.03	**>2**	**>2**	**0.5**	**0.5**
pGDP1:*vim-1*	VIM-1	0.016	0.008	0.03	0.03	**>2**	**>2**	**0.25**	**0.25**
pGDP1:*imp-1*	IMP-1	0.016	0.016	0.03	0.03	**>2**	**>2**	0.016	0.016
pGDP1:*ctx-m-55*	CTX-M-55	**0.5**	0.016	**0.5**	0.03	**0.12**	0.03	0.016	0.008
pGDP1:*cmy-42*	CMY-42	**>2**	**0.12**	**2**	0.06	**>2**	0.06	**0.03**	0.008
pGDP1:*shv-1*	SHV-1	**0.25**	0.016	**0.5**	0.03	**1**	0.06	**0.06**	0.008

a MICs done in ID-MHB; ATM: aztreonam; 043: MLEB-22043; CAZ: ceftazidime; CFDC: cefiderocol; AVI: avibactam. Avibactam used at a fixed concentration of 4 μg/mL. Bold values indicate ≥4-fold increase in MIC compared to vector control.

### Broad Spectrum Antibacterial Activity of MLEB-22043 plus Avibactam against Multidrug-Resistant Pathogens

To further characterize MLEB-22043, we tested it against additional resistant Gram-negative pathogens. *K. pneumoniae* KPNIH1 is a KPC-3 carbapenemase-producing clinical isolate and the parent strain of *K. pneumoniae* MKP103, a Δ*kpc-3* mutant.^59,60^*K. pneumoniae* KPNIH1 was resistant to both MLEB-22043 and aztreonam, whereas the *kpc-3* deletion strain *K. pneumoniae* MKP103 was susceptible (Table 7). Avibactam sensitized *K. pneumoniae* KPNIH1 to MLEB-22043 and aztreonam, implicating the KPC carbapenemase in resistance to both compounds. Clinical isolates *K. pneumoniae* C0026, C0612, C0650 and *E. coli* C0005, which are predicted to encode several different β-lactamases, were resistant to aztreonam and had elevated but detectable MICs to MLEB-22043. Addition of avibactam sensitized these strains to both compounds. Both *K. pneumoniae* C0612 and C0650 express the worrisome metallo-β-lactamase NDM-1 and are highly resistant to meropenem, ceftazidime, and ceftazidime plus avibactam (Table S8).

**Table 7:** Activity of MLEB-22043 and Aztreonam, with or without Avibactam, against Gram Negative Pathogens

		MIC (μg/mL)^a^			
strain	predicted β-lactamases^b^	043^c^	043 + AVI^c^	ATM^d^	ATM + AVI^d^
K. pneumoniae KPNIH1	KPC-3, TEM-1, SHV-11	**>16**	0.5	**>16**	0.5
K. pneumoniae KPNIH1 Δ*kpc-3* (MKP103)	TEM-1, SHV-11	0.25	0.25	0.5	0.25
K. pneumoniae C0026	CTX-M-15, SHV-1, OXA-48, OXA-1	**16**	0.12	**>16**	0.25
K. pneumoniae C0612	SHV-1, CTX-M-14, NDM-1, CTX-M-15, OXA-48	2	0.06	**>16**	0.25
K. pneumoniae C0650	SHV-27, NDM-1, CTX-M-15, OXA-232, OXA-9, TEM-55, CMY-23	**8**	0.25	**>16**	0.5
E. coli C0005	CMY-2	**16**	1	**>16**	0.5
A. baumannii AB5075	OXA-23, ADC-11, OXA-69, GES-11	**>16**	2	**>16**	**>16**
A. baumannii ATCC 17978	OXA-259, ADC-95	**>16**	2	**>16**	**>16**
A. baumannii ATCC 19606	OXA-98, ADC-158	0.25	0.5	**16**	**16**
A. baumannii C0015	OXA-90, ADC-2	1	1	**>16**	**>16**
A. baumannii C0044	OXA-100, ADC-79	0.5	1	**>16**	**>16**
A. baumannii C0074	OXA-66, ADC-73, OXA-23	0.5	2	**>16**	**>16**
A. baumannii C0092	OXA-66, ADC-73, OXA-23	2	2	**>16**	**>16**
P. aeruginosa C0292	PDC-5, OXA-50	0.12	<0.03	**>16**	**>16**
P. aeruginosa C0334	OXA-486, PDC-7	8	0.12	**>16**	**16**
P. aeruginosa C0177	OXA-488, PDC-2	0.12	<0.03	**>16**	**16**
P. aeruginosa C0029	OXA-488, PDC-2	0.5	0.06	**>16**	**16**
P. aeruginosa C0410	OXA-488, PDC-2	<0.03	<0.03	**>16**	**>16**
P. aeruginosa C0028	PDC-3	<0.03	<0.03	**16**	**16**
P. aeruginosa C0263	PDC-1, OXA-50	<0.03	<0.03	**16**	**16**
P. aeruginosa C0293	PDC-5, OXA-50	0.12	0.12	**16**	**16**
P. aeruginosa C0070	OXA-486, PDC-3	<0.03	<0.03	8	8
P. aeruginosa C0089	OXA-50, PDC-7	0.06	<0.03	8	8
P. aeruginosa PAO1	PDC-1	0.06	0.06	8	8

a Bold values indicate nonsusceptible based on CLSI breakpoints.^58^ Aztreonam breakpoints used as surrogate for MLEB-22043.

b Predictions from genome sequence by CARD.^74^

c 043: MLEB-22043, AVI: avibactam (at 4 μg/mL); tested in ID-MHB.

d ATM: aztreonam, AVI: avibactam (at 4 μg/mL).

Surprisingly, *A. baumannii* AB5075 was resistant to aztreonam, aztreonam plus avibactam, and MLEB-22043, but sensitive to MLEB-22043 plus avibactam (Table 7). Tests on an additional six *A. baumannii* strains showed all were resistant to aztreonam and aztreonam plus avibactam, but sensitive to MLEB-22043 on its own. These data suggest that, in contrast to aztreonam, MLEB-22043 readily penetrates the outer membrane of *A. baumannii*. In the case of *A. baumannii* AB5075, the GES-11 enzyme is likely responsible for MLEB-22043 resistance in this strain since the combination of MLEB-22043 plus avibactam was effective.

Finally, we examined the effect of avibactam on MLEB-22043 and aztreonam against a subset of the *P. aeruginosa* isolates previously tested (Table 4). Consistent with previous work, avibactam failed to enhance aztreonam activity against *P. aeruginosa* (Table 7).^64^ On the contrary, while MLEB-22043 generally had low MICs against these strains, avibactam further lowered the MICs in some cases (Table 7). For example, against *P. aeruginosa* strains C0292, C0177, and C0029, avibactam reduced MLEB-22043 MICs by ≥8-fold. For *P. aeruginosa* strain C0334, which showed the highest MIC of all *P. aeruginosa* strains, the MIC was reduced 64-fold. While it is not clear why MLEB-22043 is potentiated by avibactam against these strains, MIC testing with additional antibiotics revealed that these strains had high levels of antibiotic resistance, including resistance to meropenem, ceftazidime, and ceftazidime plus avibactam (for strains C0292 and C0334), suggesting their β-lactamase content or expression levels may be a factor (Table S8). We also found that avibactam improved the activity of SH-263, SH-267, and ampicillin in some cases, suggesting these compounds similarly benefit from protection against β-lactamases (Table S9).

Together, these data show that the combination of MLEB-22043 plus avibactam has broad-spectrum antibacterial activity against highly drug-resistant pathogens, including those expressing metallo-β-lactamases. Although both MLEB-22043 and aztreonam are inhibitory against resistant Enterobacterales when combined with avibactam, only MLEB-22043 has activity against *A. baumannii* and *P. aeruginosa*, two clinically important and difficult-to-treat pathogens. Importantly, exposure of mammalian Human Embryonic Kidney (HEK) 293 cells to MLEB-22043 at concentrations up to 128 μg/mL revealed no significant toxicity under these conditions (Figure S6), supporting further development of this compound.

## Discussion

The use of physiologically relevant growth media has deepened our understanding of bacterial pathogens and improved our ability to find new antibiotics.^5,75−77^ We and others have shown that human serum is an excellent mimic of the host environment, enabling the discovery of compounds with in vivo antibacterial activity.^32,34,36,77−81^ The work presented here demonstrates that serum can also be used to detect growth-enhancing synthetic siderophores. Serum is inherently iron-limited due to the iron-sequestering activity of transferrin, and is thus a sensitive growth media to detect chemical perturbants of bacterial iron homeostasis.^36^ Our reanalysis of high throughput serum screening data shows that this concept extends to compounds that liberate iron for use by bacterial pathogens and thus provides a platform to discover both antibacterial compounds and growth promoters.

Many of the growth promoting compounds from our screen contained catechol groups, including several catecholamines. This is likely due to several factors. First, two of our screening libraries contained FDA-approved and bioactive compounds, and therefore were enriched for this widely used class of compounds. Second, the catechol group is clearly an excellent synthetic siderophore, as evidenced by its routine use as a siderophore partner in antibiotic conjugates and ability to be taken up through multiple TBDTs.^16,17,54,65,82−88^ Catecholamines can liberate iron from serum transferrin, leading to enhanced growth of bacterial pathogens.^89−91^ This has been shown for several catecholamines, including epinephrine, norepinephrine, dopamine, isoprenaline, and dobutamine, which were all detected in our high-throughput screen. Interestingly, the administration of dopamine significantly enhanced the growth of *Salmonella* during mouse infection, worsening outcomes.^10^ Thus, it is likely these all represent viable partners for conjugation. We also identified several plant catechols as hits in our screen, which were recently shown to be both substrates and inducers of bacterial iron uptake pathways.^92^ Additionally, we found compounds that, while still containing catechols, were more elaborate in structure, including fenoldopam, tannic acid, MAC-0464124, and MAC-0464391. The iron-binding and growth promoting mechanism of MAC-0461611, which contains *meta-*hydroxyl groups, may be an interesting compound for follow up studies. The fact that we identified known chelators in our screen, such as deferiprone and its analog MAC-0431972, provided an additional level of validation, particularly since this scaffold has been widely used in synthetic siderophore-antibiotic conjugates.^43,44,47,47^ Five compounds were confirmed as growth promoters but did not bind iron, and four of these, including MAC-0463399, MAC-0359393, MAC-0440274, and MAC-0377328, showed relatively potent growth enhancing activity. While it remains possible that the assays we used to detect iron binding were not adequately sensitive, it is tempting to speculate that these compounds enhance growth by some novel mechanism(s). This could include binding of siderocalin, a mammalian protein present in serum that sequesters catecholate siderophores,^93^ or inhibition of other antimicrobial factors in serum. MAC-0377328 may present a particularly compelling case for additional work, given its phenotype in the CAS assay resembled that of the iron-only control. Future work to conjugate these compounds to antibiotics will allow more in-depth study of their growth enhancing mechanisms.

By ranking the relative efficacy of growth promotion, we determined that 6,7-dihydroxycoumarin, also found as part of MAC-0433454, was the most potent growth-promoting pharmacophore. Our experiments with 7-hydroxycoumarin showed that the dihydroxy moiety was essential for activity, but it remains to be seen whether other features of the coumarin contribute to their potency. Interestingly, 6,7-dihydroxycoumarin derivatives may also have antibiofilm activity against some bacteria and fungi.^94^ The hydroxyquinolines were also of interest, since this structure was identified in several hit compounds and showed good potency in the growth promotion assay. Furthermore, conjugation of 8-hydroxyquinoline with ciprofloxacin improved activity against *Chlamydia trachomatis*, but not other Gram-negative or positive bacteria.^95^ Our initial goal was to provide proof-of-concept that our serum screen was, in fact, identifying synthetic siderophores useful for the design of antibiotic conjugates to target Gram-negative bacteria. The 6,7-dihydroxycoumarin derivatives (S4 and S7) and the 8-hydroxyquinoline derivatives (S2 and S5) rescued the growth of siderophore-deficient *E. coli* and *P. aeruginosa* in low-iron media, although the magnitude of growth rescue differed between these species, likely due to differences in TBDT repertoire, their expression levels, and promiscuity. Importantly, the ampicillin conjugates SH-263 and SH-267 both showed iron-dependent activity against susceptible strains. The 6,7-dihydroxycoumarin conjugate SH-267 was more potent than SH-263 and had activity against *P. aeruginosa*, prompting us to further explore this synthetic siderophore, ultimately leading to the synthesis of the aztreonam conjugate MLEB-22043. Aztreonam was chosen for its resistance to metallo-β-lactamases and extensive previous work done with siderophore-monobactam conjugates, including compounds that reached late preclinical and clinical studies.^14,43,44,85,96^ That previous work confirmed that siderophore conjugation could potentiate aztreonam activity against *P. aeruginosa* and *A. baumannii*, two species where the parent antibiotic has poor activity. MLEB-22043 was highly active against several bacterial species and, as with SH-267 and cefiderocol, this activity was iron-dependent. Notably, MLEB-22043 showed >100-fold increased activity against *P. aeruginosa* compared to aztreonam (on a molar basis), providing strong evidence that 6,7-dihydroxycoumarin was responsible for delivering MLEB-22043 through the bacterial outer membrane. The MIC_90_ of 0.5 μg/mL for MLEB-22043 against clinical *P. aeruginosa* isolates is well below the aztreonam susceptibility breakpoint of ≤8 μg/mL for aztreonam, and further highlights its significantly improved activity.^58^ Development of monobactam conjugate MB-1 and monocarbam conjugate SMC-3176, both of which used the hydroxypyridone siderophore, were discontinued due to a lack of correlation between in vitro MICs with in vivo activity against *P. aeruginosa*.^44,96^ Through a mechanism that remains to be fully understood, these compounds were unexpectedly inactive against some *P. aeruginosa* strains during in vivo experiments, a phenomenon termed “adaptive resistance”. Interestingly, this efficacy disconnect was not seen for cefiderocol; this may be due to the choice of antibiotic partner, or, as has been speculated by others, the choice of siderophore moiety.^97^ MLEB-22043, with its unique synthetic siderophore, may also bypass these challenges, though this requires extensive in vivo investigation to determine.

Susceptibility profiling of an overexpression library of TBDTs in *P. aeruginosa* showed that MLEB-22043 uses similar transporters as cefiderocol and other siderophore conjugates, with PiuA, PirA, and PfuA identified as uptake routes.^65^ Additional TBDTs which we did not identify here were previously implicated in uptake of conjugates cefiderocol, BAL30072, and MC-1; this may be due to differences in TBDT expression levels or the strain backgrounds between studies.^65^ To confirm our results, we tested previously isolated mutants of *P. aeruginosa* PAO1 with increased resistance to MB-1 and cefiderocol.^44^ We found that these strains’ resistance patterns toward MLEB-22043, MB-1 and cefiderocol were similar when tested in MHB. Interestingly, when tested in ID-MHB, the MICs were essentially unchanged compared to the wild-type strain, except for the *piuA pirA* double mutant. Even then, using clinical breakpoints, the *piuA pirA* strain would still be considered susceptible to cefiderocol. These results underscore that in iron-limited media there are many routes to import for this class of compounds. Future work to identify the suite of uptake pathways used by these conjugates under physiological conditions will improve our ability to design effective antibiotics.

Several monobactam and monocyclic siderophore conjugates have shown increased resistance to β-lactamases. The hydroxypyridone conjugates MB-1, MC-1 (a monocarbam), and SMC-3176 were insensitive to inactivation by representative enzymes from all classes of β-lactamases, including carbapenemases, extended-spectrum β-lactamases, and metallo-β-lactamases.^44,96,98^ Although we assessed only a subset of β-lactamase enzymes, our data suggest that MLEB-22043 is resistant to hydrolysis by metallo-β-lactamases but remains susceptible to serine β-lactamase. This finding explains MLEB-22043’s lack of activity against some of the resistant Enterobacterales strains possessing serine β-lactamases. Fortunately, the combination of MLEB-22043 with avibactam was effective against every strain tested in this work, including *K. pneumoniae* C0612 and C0650, which encode the NDM-1 metallo-β-lactamase along with several additional serine-β-lactamases. The combination of aztreonam-avibactam was also effective against the Enterobacterales strains we tested. Indeed, this combination is undergoing clinical trials, and off-label uses have shown promise against some of the most difficult to treat metallo-β-lactamase producing pathogens, which often coexpress serine β-lactamases.^99^ However, unlike MLEB-22043 plus avibactam, aztreonam plus avibactam is not effective against *P. aeruginosa* or *A. baumannii*.^100^ Thus, MLEB-22043 in combination with avibactam represents a truly broad-spectrum combination compared to aztreonam-avibactam.

It is important to note that the original high throughput screen was designed to find compounds with antibacterial activity, and thus a more appropriately designed screen may increase the sensitivity to detect growth enhancers. Additionally, we envision modifications to our screening approach that would allow for the detection of synthetic siderophores that use specific TBDTs for uptake. For example, bacterial mutants lacking endogenous siderophore production would fail to grow in serum unless an exogenous synthetic siderophore was added. By comparing the growth rescue of various mutants lacking siderophore production plus at least one TBDT, the synthetic siderophore mechanism of uptake could quickly be established. By extension, these strains could be used in a differential screening approach to target a specific TBDT pathway. Performing such screens with representatives from a range of bacterial species would generate a toolbox of both broad- and narrow-spectrum siderophore partners for conjugates. While these types of growth rescue experiments are common when testing synthetic siderophores in low iron media,^87,101^ to our knowledge they have not been used in an unbiased high-throughput approach for siderophore discovery. In addition, most growth recovery approaches use media that lacks iron, or is artificially lowered through the addition of nonbiological chelators, whereas serum contains sufficient iron but is sequestered by host transferrin. The work of Kline et al. perhaps represents one of the most broad searches for synthetic siderophores.^102^ In that work, the authors manually curated a list of 352 commercially available compounds with structural homology to catechols or described metal binding activity in a biological context. These were then assayed for growth promotion of a siderophore-deficient mutant in media containing the chelator dipyridyl. While they detected several noncatechol compounds with growth promoting activity, antibiotic conjugates synthesized with these compounds failed to enhance antibacterial activity.

In total, we believe that this work begins to address growing calls for innovation in the selection of siderophore partners for antibiotic conjugates,^14^ and provides a platform to discover the next generation of synthetic siderophores for Trojan Horse antibiotics.

## Materials and Methods

### Identification of Growth Promoting Compounds

The normalized data from our previous high-throughput screening assay was used to identify compounds that resulted in increased absorbance at the end point read of the serum growth screen.^36^ A total of 4 screening compound libraries were previously screened: Pharmakon, CCC, Gram-negatives, and Glyconet. For the CCC, Gram-negatives, and Glyconet libraries, hits were defined as compounds that resulted in an absorbance value at least 3 standard deviations greater than the mean. For the Pharmakon library, which has 1600 pharmacologically active compounds and therefore a high active rate and standard deviation, we used a 2 standard deviation cutoff. The number of compounds screened and the number of hits from each library can be found in Figure S1, and the hit compounds and structures from each library are found in Table S1.

### Bacterial Strains, Growth Conditions and Chemical Compounds

All bacterial strains are listed in Table S10. Predictions of β-lactamase genes were done using the CARD database from genome sequences.^74^ Detection of siderophore biosynthetic gene clusters in *K. pneumoniae* MKP103 was done with antiSMASH v7.1.0^103^ using the genome (accession: NZ_CP008827.1) and plasmid (accession: NZ_CP008829.1, NZ_CP008830.1, NZ_CP008828.1) sequences of *K. pneumoniae* KPNIH1 (the parental strain from which MKP103 is derived), finding only enterobactin biosynthesis genes. Bacteria were routinely cultured in cation-adjusted Mueller Hinton II Broth (MHB) or MH agar (BD). Iron-depleted MHB was prepared using Chelex-100 (Bio-Rad) as recommended by CLSI methods,^57,58^ and, where appropriate supplemented with 100 μM FeCl_3_. Preparation of 50% serum is described below.

All compounds used in this study are listed in Table S2. Where applicable, compounds were obtained from commercial sources as indicated. Chemical synthesis of precursors and conjugates SH-263, SH-267, and MLEB-22043 is described below.

### Growth Promotion Assays

Commercial human serum (BioIVT) was diluted to 50% with 1xM9 salts plus 25 mM sodium bicarbonate and 1× redox dye mix A (Biolog Inc.) as previously described.^36^ Serum was frozen at −80 °C and thawed at 37 °C prior to use. *K. pneumoniae* MKP103 was grown overnight on MH agar and colonies resuspended in 0.85% NaCl to an OD_600_ of 1.0. For growth promotion experiments done in MHB, resuspended *K. pneumoniae* MKP103 was inoculated at 7 μL per 20 mL of media, yielding a starting inoculum of ∼3 × 10^5^ CFU/mL, as per CLSI guidelines.^58^ For 50% serum, 70 μL of *Klebsiella pneumonia* MKP103 was inoculated per 20 mL of 50% serum, yielding a starting inoculum of ∼3 × 10^6^ CFU/mL which allows for adequate growth, as described previously.^36^ 384-well plates were prepared by adding 0.5 μL of 10, 31.6 or 1 mM of compound dissolved in DMSO to each well, or DMSO only as a control. Then, 49.5 μL of the serum or MHB solutions were added, resulting in final compound concentrations of 100, 31.6, or 10 μM. Uninoculated media served as a sterility control. Plates were incubated in a plate reader at 37 °C and the OD_600_ was read every 20 min. Experiments were performed in biological triplicate. Growth enhancement in serum was assessed as the time in hours it took for cultures to reach an OD_600_ of 1.0. Statistical significance compared to the DMSO control was determined by one-way ANOVA with Dunnett’s multiple comparisons test (GraphPad Prism).

### CAS and UV–Vis Iron-Binding Assays

The CAS assay was performed following previously published protocols.^50,51^ Briefly, 1.5 mL of a 1 mM FeCl_3_ solution in 10 mM HCl was added to 7.5 mL of a 2 mM CAS solution. The iron-loaded CAS solution was then added to 25 mL of a 2.4 mM Cetrimonium bromide (CTAB) solution. Finally, 50 mL of a 1 M 4-Morpholineethanesulfonic acid (MES) solution was added, and the final volume brough to 100 mL with dH_2_O, resulting in the final CAS reagent. Some protocols include the optional addition of a shuttling agent, 5-sulfosalicylic acid, which we found was not required and omitted. For the assay, 3 μL of a 10 mM solution of each compound was added to a 384-well plate followed by 57 μL CAS reagent. The plates were incubated for 1 h at room temperature in the dark and then absorbance was read at 630 nm. A significant reduction in absorbance compared to a DMSO control, as determined by one-way ANOVA with Dunnett’s multiple comparisons test (GraphPad Prism), was considered positive for iron mobilization away from the CAS dye to the synthetic siderophore.

For the colorimetric UV–vis iron-binding assay,^49,52^ 5 μL of a 10 mM solution of each compound (dissolved in DMSO) was added to a 384-well plate followed by 5 μL of a 10 mM FeCl_3_ solution (dissolved in water). The plates were then spectrophotometrically scanned between 350 and 900 nm with 20 nm steps. Controls consisted of compounds with water, DMSO with iron, and DMSO with water. Shifts in absorbance in the presence of iron were used to detect iron binding. The CAS and UV–vis assays were performed in triplicate.

### Growth Recovery Assays

Growth recovery assays were performed as described in.^56^ The *E. coli* wild type strain BW25113 and the mutant strains Δ*entA* was grown in 1× LB medium (5 mL) overnight at 37 °C and 180 rpm. The *E. coli* mutants were always grown in the presence of 50 μg/mL Kanamycin. The *P. aeruginosa* wild type strain PAO1 and the mutant strain PAO1 Δ*pvdE* Δ*pchF* were grown in 1x LB medium (5 mL) overnight at 37 °C and 180 rpm. The next morning the overnight inoculums were diluted 1:100 in 20 mL LB-medium, and grown to OD_600nm_ = 0.5 within 1–2 h at 37 °C and 180 rpm. Fifteen ml of the dilution culture were pelleted by centrifugation at 4500*g*, 5 min and 4 °C. The supernatant was discarded and the pellet was washed twice with iron-free 1× LMR (5 mL) or 1× PBS. The OD_600nm_ of the bacterial suspension was adjusted 0.01 with iron-free 1× LMR with glycerol as carbon source, prepared as previously described.^56^ Compound stocks in DMSO were added to iron-free or iron-supplemented (20 μM FeCl_3_) LMR media to yield a 20 μM compound concentration. The dilution was prepared under a laminar flow bench with sterile medium, sterile microcentrifuge tubes and sterile filtered iron solutions. The microcentrifuge tubes were vortexed and the tubes were incubated on an shaker at 600 rpm, 25 °C overnight. The complex formation was indicated by a slight purple color of compounds with iron in 1xLMR. The control solution of siderophore containing no iron did not show any coloring. The tubes were collected from the shaker in the morning, and 25 μL of the 20 μM precomplexed compound was added per well into a 384 well plate under a laminar flow bench. Twenty-five μL of bacterial suspension with a OD_600nm_ = 0.01 in iron-free 1× LMR with glycerol as carbon source was added to the compound in the plate in a 1:1 dilution to yield the final 10 μM compound concentration as well as a final 10 μM iron-concentration (or iron-free). Empty wells were filled with 50 μL 1× LMR with glycerol as a sterile control, the plate was sealed with parafilm and incubated in a humid chamber at 37 °C for 48 h. Then the OD_600nm_ was determined with a plate UV–vis spectrometer.

### MIC Determinations

MICs were determined using the broth microdilution method following CLSI protocols.^58^ Briefly, bacterial strains were cultured on MH-agar plates and resuspended in 0.85% saline. Plates containing serial dilutions of the compounds to be tested were inoculated with a suspension containing 2–5 × 10^5^ cfu/mL of the relevant bacterial strain in the indicated growth media. MICs were determined visually after 18 h incubation at 37 °C, and all were performed in duplicate. For the MIC_90_ experiment with aztreonam and MLEB-22043, 95 clinical isolates plus 2 laboratory isolates (*P. aeruginosa* PAO1 and PA14) were included. The MICs for each strain were binned into their respective distributions to calculate the MIC_90_ for both compounds, defined as the concentration of compound required to inhibit 90% of isolates.

### Chemical Synthesis

Commercially obtained chemicals were used without further purification. All organic solvents were of HPLC-grade purity. All reactions were performed under inert atmosphere with oven-dried glassware unless stated otherwise. Dried solvents were purchased in water-free form. Reaction progress was controlled by LC-coupled MS.

Purification was performed by Reverse-Phase High Performance Liquid Chromatography (RP-HPLC) with a Phenomenex Luna C18 RP-column (5 μm C18(2) 100 Å, 250 × 21.2 mm, 00G-4252-P0-AX, flow rate 10 mL/min) on a Thermo Fisher Scientific Dionex Ultimate 3000 HPLC-System. Product containing fractions were identified by LCMS and lyophilized to dryness. NMR analysis was performed on Bruker Avance III or Bruker Avance III HD instruments. ^1^H NMR-spectra were recorded at 400 MHz, 500 or 700 MHz, ^13^C NMR-spectra at 101 MHz, 126 or 176 MHz, respectively. Chemical shifts δ are stated in ppm, relative to the residual solvent signal. Peak multiplicity is stated in short forms: s (singlet), bs (broad singlet), d (doublet), t (triplet), q (quartet), m (multiplet), dd (doublet of doublets), td (triplet of doublets). For acetylated compounds 0.1% deuterated acetic acid was added to the solvent to prevent deacetylation. NMR data can be found in Data set S1 in the Supporting Information.

LCMS was conducted with an Agilent 1100 HPLC System with a DAD detector and an API 150 EX Quadrupole Mass Detector with Electro Spray Ionization (ESI) (MeCN/H_2_O + 0.1% formic acid) or with a Dionex Ultimate 3000 HPLC System with a DAD Detector and a Bruker Amazon Ion Trap Mass Detector with ESI coupling. High Resolution Mass Spectroscopy was conducted with a Dionex Ultimate 3000 HPLC System equipped with a DAD Detector and a Bruker MAXIS HD QTOF Mass Detector with ESI.

#### 5-(Chloromethyl)quinolin-8-yl (**S1**)

Hydroxyquinoline (4.67 g, 32.2 mmol, 1 equiv) was dissolved in 40 mL of 12 M HCl and cooled to 0 °C in an ice bath. 37% Formaldehyde (5.12 mL, 68.7 mmol, 2.15 equiv) was added followed by ZnCl_2_ (0.48 g, 3.5 mmol, 0.11 equiv). The ice bath was removed, and the reaction was left to stir overnight at room temperature. The mixture was filtered and washed with excess cold acetone to isolate 5-(chloromethyl)quinolin-8-ol (**S1**) as a yellow solid (6.0527 g, 26.3 mmol, 82%).

*R*_*f*_ 0.55 (10% MeOH/DCM). ^1^H NMR (400 MHz, DMSO-*d*_6_): δ 9.12 (d, *J* = 8.8 Hz, 1H), 9.09 (dd, *J* = 5.0, 1.4 Hz, 1H), 8.05 (dd, *J* = 8.7, 5.0 Hz, 1H), 7.83 (d, *J* = 8.0 Hz, 1H), 7.40 (d, *J* = 8.0 Hz, 1H), 5.31 (s, 1H). ^13^C NMR (101 MHz, DMSO): δ 150.5, 145.2, 140.9, 131.8, 131.5, 127.6, 124.38, 122.7, 114.2, 43.3.

#### 5-(Azidomethyl)quinolin-8-yl (**S2**)

5-(Chloromethyl)quinolin-8-ol (**S1**) (1 g, 4.33 mmol, 1 equiv) was added to a suspension of sodium azide (1.3 g, 17 mmol, 3 equiv) in 50 mL of acetone at rt. The mixture heated to reflux overnight, and after 16 h the solvent was evaporated under reduced pressure. The crude reaction mixture was dissolved in chloroform and washed with water and brine, and the organic phase was dried with sodium sulfate. The solution was concentrated and purified using column chromatography (0–30% EtOAc/Hexanes) resulting in 5-(azidomethyl)quinolin-8-ol (**S2**) as a white solid in 61% yield.

*R*_*f*_ 0.34 (20% EtOAc/Hex). ^1^H NMR (400 MHz, CDCl_3_): δ 8.84 (dd, *J* = 4.2, 1.5 Hz, 1H), 8.42 (s, 1H), 8.38 (dd, *J* = 8.5, 1.5 Hz, 1H), 7.54 (dd, *J* = 8.5, 4.2 Hz, 1H), 7.44 (d, *J* = 7.7 Hz, 1H), 7.13 (d, *J* = 7.8 Hz, 1H), 4.65 (s, 2H). ^**13**^**C NMR** (101 MHz, CDCl_3_): δ 153.2, 148.1, 138.9, 132.8, 129.6, 127.0, 122.5, 121.6, 109.0, 52.4.

#### 4-(Chloromethyl)-6,7-dihydroxy-2*H*-chromen-2-one (**S3**)

Triacetoxybenzene (4.713 g, 1 equiv, 18.69 mmol) and ethyl chloroacetoacetate (6.150g, 1.6 equiv, 29.8 mmol) were solubilized in 5 mL of perchloric acid at room temperature and stirred overnight. After 16 h the reaction was poured into 100 mL of ice-water and let sit until a yellow-white precipitate formed. The precipitate was collected through vacuum filtration and rinsed with cold water. The crude product was recrystallized from methanol providing 4-(chloromethyl)-6,7-dihydroxy-2*H*-chromen-2-one (**S3**) as a pale yellow solid (2.498 g, 11.0 mmol, 59%).

*R*_*f*_ 0.44 (10% MeOH/DCM). ^1^H NMR (400 MHz, DMSO-*d*_6_): δ 10.36 (s, 1H), 9.45 (s, 1H), 7.11 (s, 1H), 6.78 (s, 1H), 6.39 (s, 1H), 4.89 (d, *J* = 0.9 Hz, 2H). ^**13**^**C NMR** (101 MHz, DMSO): δ 160.5, 150.7, 150.6, 142.9, 111.2, 109.4, 108.9, 102.9, 41.6. LCMS (ESI) *m*/*z*: 227.0106 calcd for C_10_H_8_ClO_4_^+^ [M + H]^+^; found, 227.0117.

#### 4-(Azidomethyl)-6,7-dihydroxy-2*H*-chromen-2-one (**S4**)

NaN_3_ (432.0 mg, 3 equiv, 6.64 mmol) was added to a solution of 4-(chloromethyl)-6,7-dihydroxy-2*H*-chromen-2-one (**S3**) (500 mg, 1 equiv, 2.21 mmol) in DMF (5 mL) at rt. After stirring for 16 h, ice cold water (15 mL) was added, and the solution was left to sit for 10 min until precipitate stopped forming. The precipitate was collected through vacuum filtration and recrystallized from methanol providing 4-(azidomethyl)-6,7-dihydroxy-2*H*-chromen-2-one (**S4**) as a red-brown solid (420.3 mg, 1.80 mmol, 81%).

*R*_*f*_ 0.43 (10% MeOH/DCM). ^1^H NMR (400 MHz, DMSO-*d*_6_): δ 9.89 (s, 2H), 6.98 (s, 1H), 6.77 (s, 1H), 6.30–6.19 (m, 1H), 4.72 (d, *J* = 1.2 Hz, 2H), 2.08 (s, 1H). ^**13**^**C NMR** (101 MHz, DMSO-*d*_6_): δ 160.5, 150.6, 149.9, 148.2, 143.0, 109.6, 109.0, 109.0, 102.9, 49.9. LCMS (ESI) *m*/*z*: 234.0509 calcd for C_10_H_8_N_3_O_4_^+^ [M + H]^+^; found, 234.0509.

#### 5-(Azidomethyl)quinolin-8-yl Acetate (**S5**)

The azido-functionalized 8HQ (**S2**) (50 mg, 0.25 mmol) was solved in MeCN (2.5 mL) and triethylamine (38 mg, 52 μL, 0.37 mmol, 1.5 equiv) was added. The solution was cooled down to 0 °C and acetyl chloride (29 mg, 27 μL, 0.37 mmol, 1.5 equiv) was added. The reaction was allowed to warm to room temperature and stirred for 30 min. Then, mQ-H_2_O (2.5 mL) was added and the aqueous phase was extracted with DCM (3 × 5 mL). The combined organic phases were washed with brine (1 × 5 mL). Acetic acid (100 μL) was added to prevent deacetylation before drying over NaSO_4_. Then, the combined organic phases were filtered and evaporated. The acetylated 8HQ (**S5**) (60 mg, quantitative) was obtained as a clear oil and was used without further purification.

^**1**^**H NMR** (500 MHz, MeOD-*d*_4_): δ [ppm] = 8.91 (dd, *J* = 4.2, 1.6 Hz, 1H), 8.61 (dd, *J* = 8.6, 1.6 Hz, 1H), 7.70–7.63 (m, 2H), 7.50 (d, *J* = 7.7 Hz, 1H), 4.86 (s, 2H), 2.46 (s, 3H).

^**13**^**C NMR** (126 MHz, MeOD-*d*_4_): δ [ppm] = 175.3, 171.4, 151.5, 148.9, 142.5, 134.7, 131.9, 129.2, 128.8, 123.4, 122.6, 52.6, 20.9, 20.8.

ESI-HRMS: C_12_H_11_N_4_O_2_^+^ calcd [M + H]^+^ 243.0877, measured [M + H]^+^ 243.0877.

#### 4-(1-((8-Acetoxyquinolin-5-yl)methyl)-1*H*-1,2,3-triazol-4-yl)butanoic Acid (**S6**)

The acetylated 8HQ (**S5**) (5.4 mg, 0.022 mmol) was dissolved in DMF (1.0 mL) and 5-hexynoic acid (3.2 mg, 3.2 μL, 0.029 mmol, 1.3 equiv) was added. Acetic acid (10 μL) was added to prevent deacetylation. Freshly prepared aqueous solutions of CuSO_4_ (100 mM, 0.011 mmol, 0.5 eq., 110 μL) and sodium ascorbate (100 mM, 0.022 mmol, 1.0 equiv, 220 μL) were mixed in an Eppendorf tube. Then, an aqueous solution of THPTA (100 mM, 0.011 mmol, 0.5 equiv, 110 μL) was added, upon which the solution turns clear. The aqueous Cu(I)-solution was added immediately to the reaction mixture. The reaction was stirred at room temperature for 1 h, before being filtered and purified by RP-HPLC (MeCN/H_2_O 0.1% AcOH). Product containing fractions were identified by LCMS and lyophilized to dryness. The product (**S6**) (6.0 mg, 0.017 mmol, 77%) was obtained as a white solid.

^**1**^**H NMR** (500 MHz, MeOD-*d*_4_): δ [ppm] = 8.90 (dd, *J* = 4.3, 1.5 Hz, 1H), 8.67 (dd, *J* = 8.7, 1.6 Hz, 1H), 7.74 (s, 1H), 7.68–7.60 (m, 2H), 7.53 (d, *J* = 7.7 Hz, 1H), 6.08 (s, 2H), 2.73–2.67 (m, 2H), 2.46 (s, 3H), 2.29 (t, *J* = 7.4 Hz, 2H), 1.90 (p, *J* = 7.4 Hz, 2H).

^**13**^**C NMR** (126 MHz, MeOD-*d*_4_): δ [ppm] = 176.9, 175.3, 151.6, 148.9, 134.1, 131.2, 129.3, 123.6, 123.5, 122.9, 51.8, 34.0, 25.7, 25.6, 20.9, 20.4, 20.2, 20.1, 19.9, 19.8.

ESI-HRMS: C_18_H_19_N_4_O_4_^+^ calcd [M + H]^+^ 355.1401, measured [M + H]^+^ 355.1400.

#### (2*S*,5*R*,6*S*)-6-((*R*)-2-(4-(1-((8-Acetoxyquinolin-5-yl)methyl)-1*H*-1,2,3-triazol-4-yl)butanamido)-2-phenylacetamido)-3,3-dimethyl-7-oxo-4-thia-1-azabicyclo[3.2.0]heptane-2-carboxylic Acid (**SH-263**)

The acid functionalized 8HQ (**S6**) (3.0 mg, 0.008 mmol) was dissolved in DCM (200 μL) under argon and DMF (20 μL) was added. The solution was cooled down to 0 °C and then oxalyl chloride (2.2 mg, 1.5 μL, 0.017 mmol, 2.0 equiv) was added. The reaction was stirred for 1 h at 0 °C. The reaction progress was controlled by quenching an aliquot with MeOH. Formation of the Me-ester can be observed by LCMS and indicates formation of the corresponding acyl chloride. Ampicillin (3.25 mg, 0.009 mmol, 1.1 equiv) was then added as a solution in DMF (100 μL) and triethylamine (10 μL). The reaction mixture was allowed to warm to room temperature and stirred for 1 h at room temperature. The reaction mixture was filtered and immediately purified by RP-HPLC (MeCN/H_2_O 0.1% AcOH). Product containing fractions were identified by LCMS and lyophilized to dryness. The compound (3.0 mg, 0.004 mmol, 50%) was obtained as a white solid.

^**1**^**H NMR** (700 MHz, DMSO-*d*_6_): δ [ppm] = 9.07 (d, *J* = 7.9 Hz, 1H), 8.93 (dd, *J* = 4.1, 1.5 Hz, 1H), 8.69 (dd, *J* = 8.7, 1.6 Hz, 1H), 8.51 (d, *J* = 8.3 Hz, 1H), 7.94 (s, 1H), 7.67 (dd, *J* = 8.6, 4.2 Hz, 1H), 7.54 (d, *J* = 7.7 Hz, 1H), 7.49 (d, *J* = 7.8 Hz, 1H), 7.43–7.39 (m, 2H), 7.33–7.29 (m, 2H), 7.27–7.24 (m, 1H), 6.05 (s, 2H), 5.73–5.69 (m, 1H), 5.52–5.48 (m, 1H), 5.38 (d, *J* = 4.0 Hz, 1H), 4.18 (s, 1H), 2.59–2.53 (m, 2H), 2.40 (s, 3H), 2.27–2.21 (m, 2H), 1.83–1.74 (m, 2H), 1.53 (s, 3H), 1.40 (s, 3H).

^**13**^**C NMR** (176 MHz, DMSO-*d*_6_): δ [ppm] = 173.4, 172.0, 171.7, 169.1, 168.9, 150.5, 147.4, 146.9, 140.7, 138.2, 132.5, 130.6, 128.2, 127.5, 127.3, 127.2, 127.0, 122.4, 122.3, 121.1, 70.4, 67.2, 63.8, 58.1, 58.0, 55.4, 49.6, 34.4, 30.4, 26.6, 25.2, 24.6, 20.7, 20.5, 20.4, 20.3, 20.2, 20.1, 20.0.

ESI-HRMS: C_35_H_36_N_7_O_7_S^+^ calcd [M + H]^+^ 686.2391, measured [M + H]^+^ 686.2390.

#### 4-(Azidomethyl)-2-oxo-2H-chromene-6,7-diyl Diacetate (**S7**)

The azido-functionalized dihydroxycoumarin (**S4**) (50 mg, 0.21 mmol) was solved in MeCN (2.5 mL) and triethylamine (65 mg, 89 μL, 0.64 mmol, 3.0 equiv) was added. The solution was cooled down to 0 °C and acetyl chloride (51 mg, 46 μL, 0.64 mmol, 3.0 equiv) was added. The reaction was allowed to warm to room temperature and stirred for 30 min. Then, mQ-H_2_O (2.5 mL) was added and the phases were separated. The aqueous phase was extracted with DCM (3 × 5 mL) and washed with brine (1 × 5 mL). Acetic acid (100 μL) was added to prevent deacetylation before drying over NaSO_4_. Then, the combined organic phases were filtered and evaporated. The acetylated dihydroxy coumarin (**S7**) (67 mg, quantitative) was obtained as a brown solid and was used without further purification.

^**1**^**H NMR** (500 MHz, MeOD-*d*_4_): δ [ppm] = 7.61 (s, 1H), 7.37 (s, 1H), 6.56 (t, *J* = 1.4 Hz, 1H), 4.73 (d, *J* = 1.4 Hz, 2H), 2.32 (d, *J* = 1.1 Hz, 6H).

^**13**^**C NMR** (126 MHz, MeOD-*d*_4_): δ [ppm] = 169.9, 169.2, 161.8, 152.8, 151.0, 146.7, 140.6, 120.1, 117.0, 114.8, 113.4, 51.4, 20.5, 20.4, 20.3, 20.2, 20.1, 19.9, 19.8.

ESI-HRMS: C_14_H_12_N_3_O_6_^+^ calcd [M + H]^+^ 318.0721, measured [M + H]^+^ 318.0721.

#### 4-(1-((6,7-Diacetoxy-2-oxo-2*H*-chromen-4-yl)methyl)-1*H*-1,2,3-triazol-4-yl)butanoic Acid (**S8**)

The acetylated dihydroxycoumarin (**S7**) (5.0 mg, 0.016 mmol) was dissolved in DMF (1.0 mL) and 5-hexynoic acid (2.3 mg, 2.3 μL, 0.020 mmol, 1.3 equiv) was added. Acetic acid (10 μL) was added to prevent deacetylation. Freshly prepared aqueous solutions of CuSO_4_ (100 mM, 0.010 mmol, 0.5 eq., 100 μL) and sodium ascorbate (100 mM, 0.020 mmol, 1.0 equiv, 200 μL) were mixed in an Eppendorf tube. Then, an aqueous solution of THPTA (100 mM, 0.010 mmol, 0.5 equiv, 100 μL) was added, upon which the solution turns clear. The aqueous Cu(I)-solution was added immediately to the reaction mixture. The reaction was stirred at room temperature for 1 h, before being filtered and purified by RP-HPLC (MeCN/H_2_O 0.1% AcOH). Product containing fractions were identified by LCMS and lyophilized to dryness. The product (**S8**) (4.5 mg, 0.010 mmol, 63%) was obtained as a white solid.

^**1**^**H NMR** (500 MHz, DMSO-*d*_6_): δ [ppm] = 8.03 (s, 1H), 7.79 (s, 1H), 7.53 (s, 1H), 5.91–5.82 (m, 3H), 2.66 (t, *J* = 7.6 Hz, 2H), 2.34 (s, 3H), 2.32 (s, 3H), 2.26 (t, *J* = 7.4 Hz, 2H), 1.82 (p, *J* = 7.5 Hz, 2H).

^**13**^**C NMR** (126 MHz, DMSO-*d*_6_): δ [ppm] = 174.2, 172.1, 168.4, 167.9, 159.2, 151.1, 149.4, 147.1, 145.1, 138.7, 123.2, 119.3, 115.6, 112.5, 33.0, 24.4, 24.3, 20.7, 20.5, 20.5, 20.4, 20.3, 20.2, 20.1, 19.9.

ESI-HRMS: C_20_H_20_N_3_O_8_^+^ calcd [M + H]^+^ 430.1245, measured [M + H]^+^ 430.1245.

#### (2*S*,5*R*,6*R*)-6-((*R*)-2-(4-(1-((6,7-Diacetoxy-2-oxo-2*H*-chromen-4-yl)methyl)-1*H*-1,2,3-triazol-4-yl)butanamido)-2-phenylacetamido)-3,3-dimethyl-7-oxo-4-thia-1-azabicyclo[3.2.0]heptane-2-carboxylic Acid (**SH-267**)

The acid functionalized dihydroxycoumarin (**S8**) (7.0 mg, 0.016 mmol) was dissolved in DCM (200 μL) under argon and DMF (20 μL) was added. The solution was cooled down to 0 °C and then oxalyl chloride (4.1 mg, 2.8 μL, 0.033 mmol, 2.0 equiv) was added. The reaction was stirred for 1 h at 0 °C. The reaction progress was controlled by quenching an aliquot with MeOH. Formation of the Me-ester can be observed by LCMS and indicates formation of the corresponding acyl chloride. Ampicillin (6.3 mg, 0.018 mmol, 1.1 equiv) was then added as a solution in DMF (100 μL) and triethylamine (20 μL). The reaction mixture was allowed to warm to room temperature and stirred for 1 h at room temperature. The reaction mixture was filtered and immediately purified by RP-HPLC (MeCN/H_2_O 0.1% AcOH). Product containing fractions were identified by LCMS and lyophilized to dryness. The compound (9.5 mg, 0.012 mmol, 75%) was obtained as a white solid.

^**1**^**H NMR** (700 MHz, DMSO-*d*_6_): δ [ppm] = 9.08 (d, *J* = 7.9 Hz, 1H), 8.53 (d, *J* = 8.1 Hz, 1H), 8.01 (s, 1H), 7.80 (s, 1H), 7.52 (s, 1H), 7.44–7.40 (m, 2H), 7.33–7.30 (m, 2H), 7.29–7.24 (m, 1H), 5.86 (s, 3H), 5.75–5.70 (m, 1H), 5.53–5.49 (m, 1H), 5.38 (d, *J* = 4.0 Hz, 1H), 4.19 (s, 1H), 2.63 (td, *J* = 7.3, 1.9 Hz, 2H), 2.33 (s, 3H), 2.32 (s, 3H), 2.29 (t, *J* = 7.4 Hz, 2H), 1.86–1.81 (m, 2H), 1.54 (s, 3H), 1.40 (s, 3H).

^**13**^**C NMR** (176 MHz, DMSO-*d*_6_): δ [ppm] = δ 172.0, 168.8, 168.3, 167.8, 159.0, 151.0 147.2, 145.0, 138.7, 128.2, 127.5, 127.2, 126.9 (extracted from HSQC), 123.1 122.7 (extracted from HSQC), 119.2, 113.8 (extracted from HSQC), 112.4, 70.4, 67.2, 63.8, 58.0, 55.1 (extracted from HSQC), 34.4, 30.4, 26.6, 25.2, 24.7, 20.5, 20.4, 20.3, 20.2, 20.1, 20.0.

ESI-HRMS: C_36_H_37_N_6_O_11_S^+^ calcd [M + H]^+^ 761.2236, measured [M + H]^+^ 761.2235.

#### Aztreonam-Alkyne (**S9**)

Aztreonam (300 mg, 0.689 mmol, 1 equiv) was added to a solution of EDC·HCl (272.4 mg, 1.378 mmol, 1.5 equiv), HOBt (211.0 mg, 1.378 mmol, 1.5 equiv) and DIPEA (0.3 mL, 1.722 mmol, 2.5 equiv) in DMF (15 mL) at 0 °C under argon. After stirring the reaction mixture at 0 °C for 30 min, propargylamine (75.9 mg, 1.378 mmol, 1.5 equiv) was added and the reaction was warmed to rt. After 16 h, the reaction was concentrated under reduced pressure and purified on an Agilent 1290 Infinity II Preparative HPLC (0 → 100% 1% Formic Acid H_2_O/MeCN, 18 min). The product coelutes with HOBt, however could be carried forward crude to the next reaction.

^**1**^**H NMR** (400 MHz, DMSO-*d*_6_): δ 9.46 (d, *J* = 8.1 Hz, 1H), 7.81 (t, *J* = 5.8 Hz, 1H), 6.98 (s, 1H), 4.56 (dd, *J* = 8.1, 2.6 Hz, 1H), 3.91 (dd, *J* = 5.9, 2.5 Hz, 2H), 3.73 (qd, *J* = 6.1, 2.6 Hz, 1H), 3.07 (t, *J* = 2.5 Hz, 1H), 1.50–1.34 (m, 9H). LCMS (ESI) *m*/*z*: 473.0908 calcd for C_16_H_21_N_6_O_7_S_2_^+^ [M + H]^+^; found, 473.0911.

### MLEB-22043

4-(Azidomethyl)-6,7-dihydroxy-2H-chromen-2-one (**S4**) (80.0 mg, 0.34 mmol, 1 equiv) was added to a solution of aztreonam-alkyne (**S9**) (162.1 mg, 0.34 mmol, 1 equiv) in a mixture of THF (1 mL), tBuOH (1 mL) and H_2_O (1 mL). Sodium ascorbate (18.9 mg, 0.10 mmol, 0.28 equiv) and CuSO_4_·5H_2_O (18.7 mg, 0.07 mmol, 0.22 equiv) were added and the solution was stirred at rt for 5 h. A second portion of sodium ascorbate (18.9 mg, 0.10 mmol, 0.28 equiv) and CuSO_4_·5H_2_O (18.7 mg, 0.07 mmol, 0.22 equiv) were added and the reaction was stirred at rt overnight. The reaction was concentrated under reduced pressure and purified on an Agilent 1290 Infinity II Preparative HPLC (0 → 100% 1% Formic Acid H_2_O/MeCN, 18 min) providing **MLEB-22043** as a brown solid (33.0 mg, 0.047 mmol, 14%).

^**1**^**H NMR** (700 MHz, DMSO-*d*_6_): δ 9.39 (d, *J* = 8.3 Hz, 1H), 8.07 (s, 1H), 7.94 (t, *J* = 6.2 Hz, 1H), 7.12 (s, 1H), 6.87 (br s, 1H), 6.77 (s, 1H), 5.80–5.77 (m, 2H), 5.33 (s, 1H), 4.53 (dd, *J* = 8.2, 2.7 Hz, 1H), 4.46 (dd, *J* = 15.5, 6.0 Hz, 1H), 4.41 (dd, *J* = 15.5, 6.0 Hz, 1H), 3.73 (dq, *J* = 2.7, 6.1 Hz, 1H), 3.17 (s, 2H), 1.41 (m, 9H). LCMS (ESI) *m*/*z*: 706.1344 calcd for C_26_H_28_N_9_O_11_S_2_^+^ [M + H]^+^; found, 706.1345.

### Construction of the *P. aeruginosa* PA14 Mutants and TonB-dependent Transporter Overexpression Strains

All primers used were purchased from IDT, reconstituted in nuclease free water to a concentration of 1 mg/mL and stored at −20 °C. Primers for the construction of TBDT overexpression library in the pHERD20T vector are provided in Table S11. Clean deletions of *piuA*,*pirA, and tonB3* were made as previously described.^104−106^ The clean deletion of *tonB2* was constructed as previously described.^107^ The primers used to make each deletion construct are list in Table S11. Clean deletion mutants were complemented with *piuA* or pirA with the arabinose-inducible expression vector pHERD20T.^104−106,108^ The primers used to make each expression construct are listed in Table S11.

### Cytotoxicity Testing

Cytotoxicity of MLEB-22043 and aztreonam was assessed by evaluating cell viability in the HEK 293 cell line. The immortalized HEK 293 cell line was maintained in DMEM media (Thermo Scientific) supplemented with 10% heat inactivated FBS (Gibco) and cultured at 37 °C in a humidified atmosphere at 5% CO_2_. Cells were plated into white optical-bottom 96-well plates (Thermo Scientific, Nunc) at a density of 6000 cells per well. The next day cells were individually treated with MLEB-22043 or aztreonam for 24 h. The compounds were tested at concentrations ranging from 128 μg/mL to 0.25 or 1 μg/mL (in serial dilution) for MLEB-22043 and aztreonam, respectively. The DMSO concentration was corrected to 0.5% in all wells. Cell metabolic activity was determined by preforming PrestoBlue assays (Invitrogen). Therefore, the presto blue reagent was added, a minimum of 5 h prior to the end of treatment, and incubated in darkness. Resorufin fluorescence was measured using a BioTek Neo microplate reader with excitation and emission wavelength set at 560 and 590 nm, respectively. The cell viability was expressed as a percentage relative to solvent-treated control cells (considered as 100% viable). The data is expressed as mean ± SD of triplicate experiments.

## Supporting Information Available

Figure S1—Putative growth-enhancing compounds identified in high-throughput screening data; Figure S2—Growth curves of *K. pneumoniae* MKP103 in MHB in the presence of the indicated compounds; Figure S3—Growth curves of *K. pneumoniae* MKP103 in 50% serum in the presence of the indicated compounds; Figure S4—UV–vis spectroscopy of compounds in the presence of iron; Figure S5—Iron-binding activity of siderophore-antibiotic conjugates; Figure S6—Cytotoxicity testing of MLEB-22043 against HEK 293 cells, Table S3—MICs of MLEB-22043, cefiderocol, and aztreonam against *E. coli* BW25113 wild-type and Δ*tonB*; Table S4—MICs of MLEB-22043, cefiderocol, and aztreonam against *P. aeruginosa* PA14 overexpressing TonB-dependent transporters; Table S5- MICs of MLEB-22043, cefiderocol, and aztreonam against *P. aeruginosa* PA14 TBDT mutant strains; Table S6- MICs of MLEB-22043 and aztreonam against *P. aeruginosa* PA14 *tonB* mutants; Table S7- MICs of MLEB-22043 and aztreonam against efflux-deficient *P. aeruginosa mPAO1*; Table S8—Activity of MLEB-22043 and other β-lactam antibiotics against *K. pneumoniae* and *P. aeruginosa* strains; Table S9—MICs of SH-263, SH-267, and ampicillin in combination with avibactam against *K. pneumoniae*, *E. coli*, and *P. aeruginosa*; Table S10—Bacterial strain list, Data set S1—Compound NMR (PDF)
Table S1—All hits from primary screen (XLSX)
Table S2—Summary data for all compounds (XLSX)
Table S11—Primers for construction of TonB-dependent transporter expression plasmid library (XLSX)
