# Supplementary material for: High-Throughput Discovery of Synthetic Siderophores for Trojan Horse Antibiotics
Source: ACS Infect Dis. 2024 Oct 22;10(11):3821–41. doi: 10.1021/acsinfecdis.4c00359 (PMC11556397; doi:10.1021/acsinfecdis.4c00359)
Supplement: Supplementary file 1 — id4c00359_si_001.pdf [file id4c00359_si_001.pdf]

Supporting Information

High-throughput discovery of synthetic siderophores for Trojan Horse antibiotics

Brent S. Weber[1,2,7], Nikki E. Ritchie[1,2], Simon Hilker[3], Derek C. K. Chan[1,2], Carsten Peukert[3], Julia P. Deisinger[1,2], Rowan Ives[1,2], Christine Årdal[4], Lori L. Burrows[1,2], Mark Brönstrup[3,5,6], Jakob Magolan[1,2], Tracy L. Raivio[7], Eric D. Brown[1,2*]

[1]Department of Biochemistry and Biomedical Sciences, McMaster University, Hamilton, Ontario, Canada L8S 4L8

[2]Michael G. DeGroote Institute for Infectious Disease Research, McMaster University, Hamilton, Ontario, Canada L8S 4L8

[3]Department of Chemical Biology, Helmholtz Centre for Infection Research Inhoffenstraße 7 38124 Braunschweig, Germany

[4]Antimicrobial Resistance Centre, Norwegian Institute of Public Health, Oslo 0213, Norway

[5]German Center for Infection Research (DZIF), Site Hannover-Braunschweig, Inhoffenstraße 7, 38124 Braunschweig, Germany

[6]Institute for Organic Chemistry (IOC), Leibniz Universität Hannover, Schneiderberg 1B, 30167 Hannover, Germany

[7]Department of Biological Sciences, University of Alberta, Edmonton, Alberta, Canada T6G 2R3

[*]Correspondence: ebrown@mcmaster.ca

Supplementary Figures

Fig. S1- Putative growth-enhancing compounds identified in high-throughput screening data.

Fig. S2- Growth curves of *K. pneumoniae* MKP103 in MHB in the presence of the indicated compounds.

Fig. S3- Growth curves of *K. pneumoniae* MKP103 in 50% serum in the presence of the indicated compounds.

Fig. S4- Uv-vis spectroscopy of compounds in the presence of iron.

Fig. S5- Iron-binding activity of siderophore-antibiotic conjugates.

Fig. S6- Cytotoxicity testing of MLEB-22043 against HEK 293 cells.

Supplementary Tables

Table S1- All hits from primary screen (excel file)

Table S2- Summary data for all compounds (excel file)

Table S3- MICs of MLEB-22043, cefiderocol, and aztreonam against *E. coli* BW25113 wild-type and Δ*tonB*

Table S4- MICs of MLEB-22043, cefiderocol, and aztreonam against *P. aeruginosa* PA14 overexpressing TonB-dependent transporters

Table S5- MICs of MLEB-22043, cefiderocol, and aztreonam against *P. aeruginosa* PA14 TBDT mutant strains

Table S6- MICs of MLEB-22043 and aztreonam against *P. aeruginosa* PA14 *tonB* mutants

Table S7- MICs of MLEB-22043 and aztreonam against efflux-deficient *P. aeruginosa*

Table S8- Activity of MLEB-22043 and other β-lactam antibiotics against *K. pneumoniae* and *P. aeruginosa* strains

Table S9- MICs of SH-263, SH-267, and ampicillin in combination with avibactam against *K. pneumoniae*, *E. coli*, and *P. aeruginosa*

Table S10- Bacterial strain list

Table S11- Primers for construction of TonB-dependent transporter expression plasmid library (excel file)

Supporting Dataset

Dataset S1- Compound NMR

Supplementary Figures

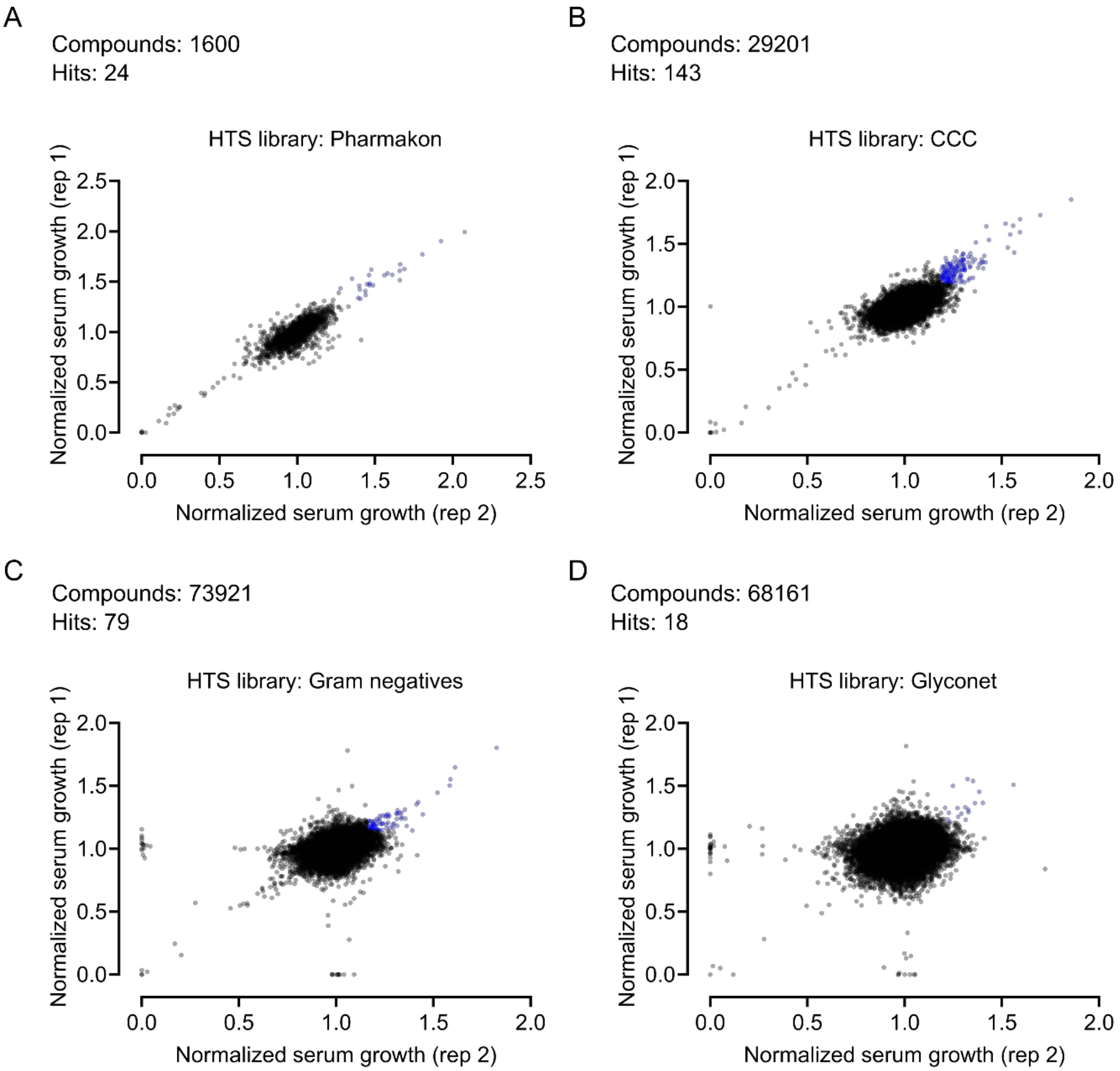

Figure S1. Putative growth-enhancing compounds identified in high-throughput screening data. The number of compounds screened and the number of growth-promoting hits identified for the A) Pharmakon small molecule library, B) CCC small molecule library, C) Gram-negatives small molecule library, and D) Glyconet small molecule library. Each compound in the library is represented as a black dot, with the growth-promoting hits (as defined in the materials and methods) colored in blue. See Table S1 for compound structures.

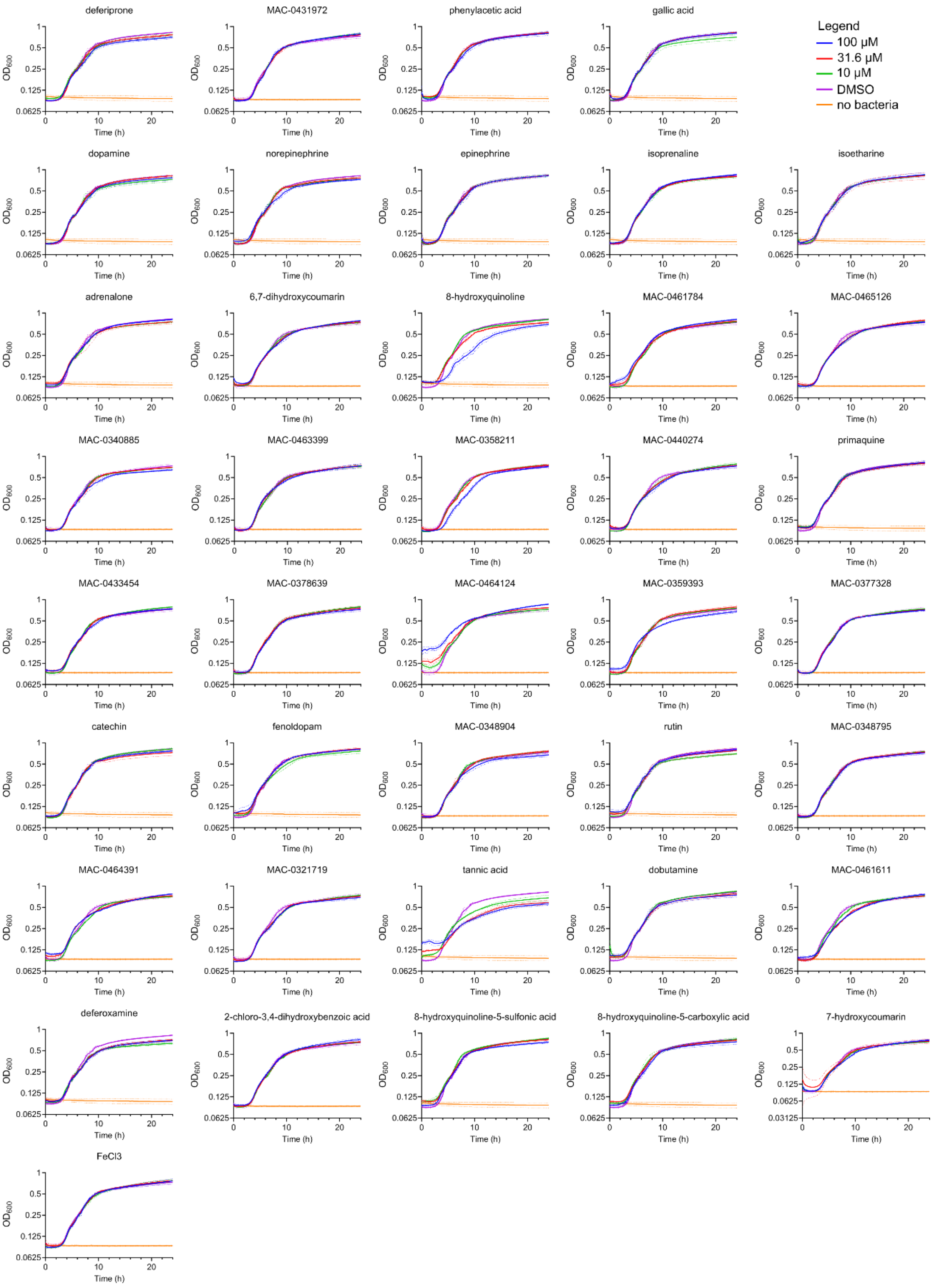

Figure S2. Growth curves of *K. pneumoniae* MKP103 in MHB in the presence of the indicated compounds. DMSO alone or compounds at 100, 31.6, or 10 μM were added to 384-well plates and inoculated with *K. pneumoniae* in MHB. Growth was monitored kinetically over 24h. Graphs show the mean ± standard deviation for 3 biological replicates.

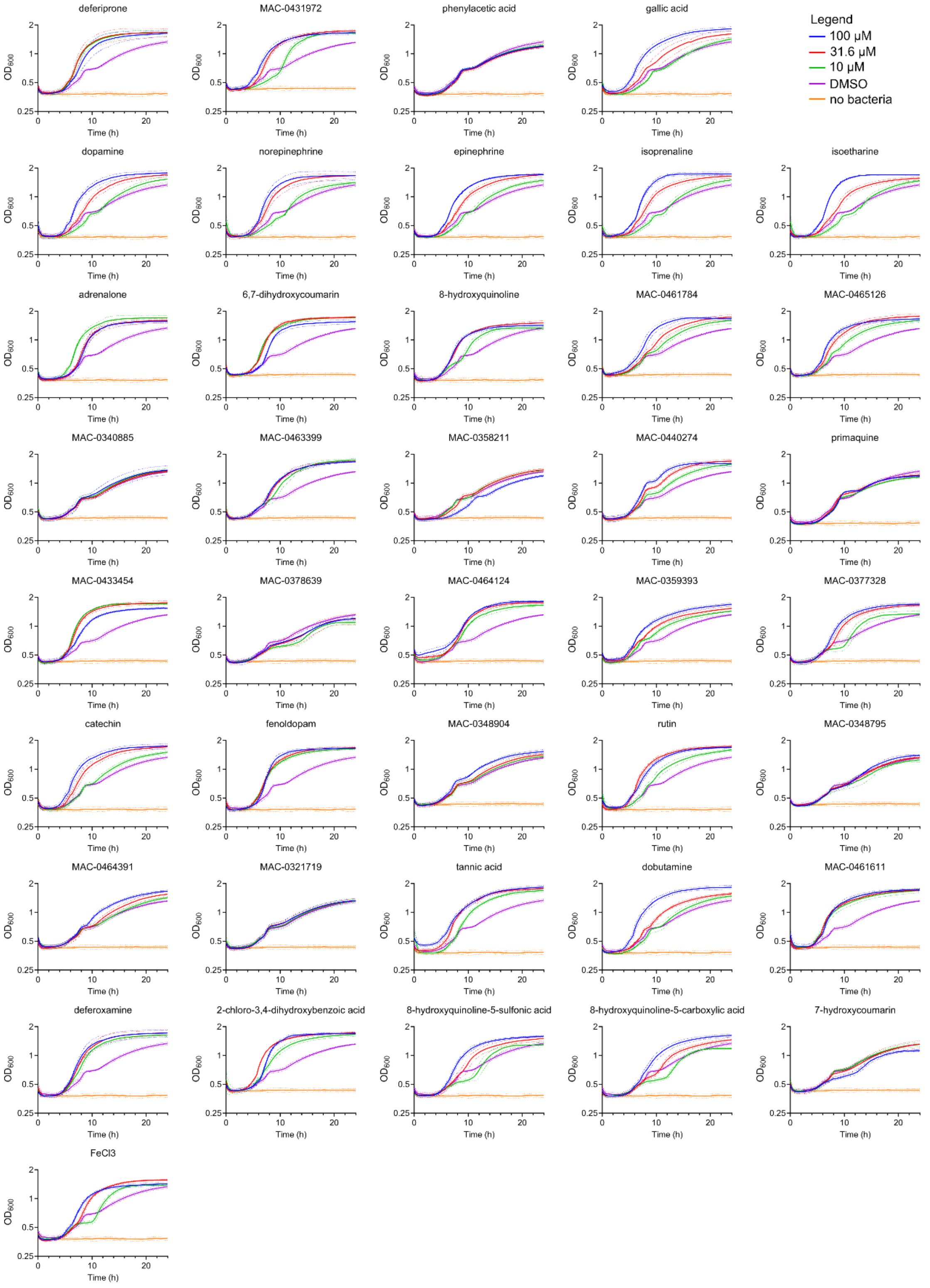

Figure S3. Growth curves of *K. pneumoniae* MKP103 in 50% serum in the presence of the indicated compounds. DMSO alone or compounds at 100, 31.6, or 10 µM were added to 384-well plates and inoculated with *K. pneumoniae* in 50% serum. Growth was monitored kinetically over 24h. Graphs show the mean ± standard deviation for 3 biological replicates.

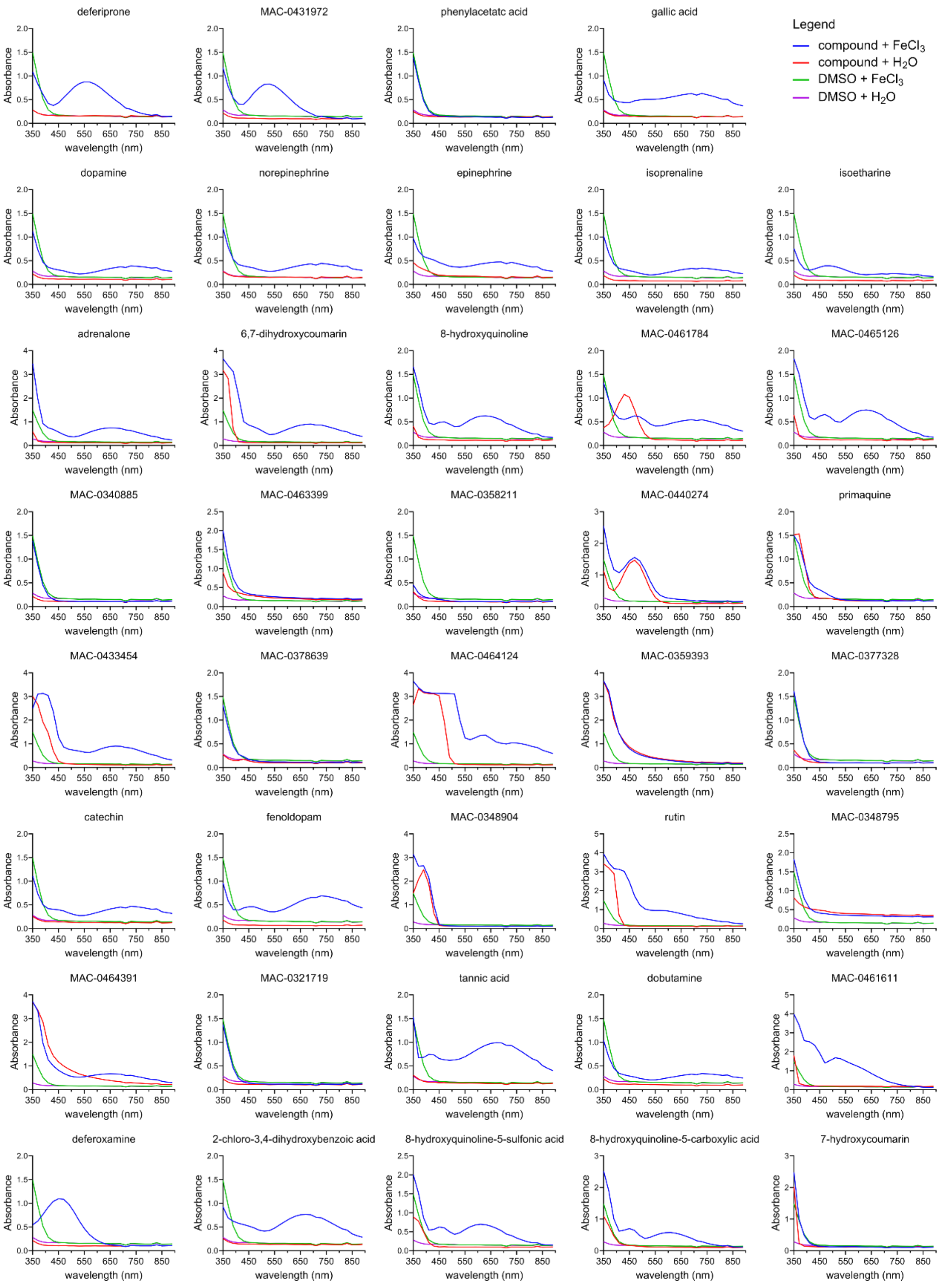

Figure S4. Uv-vis spectroscopy of compounds in the presence of iron. 5 µl of a 10 mM solution of each compound (dissolved in DMSO) was added to a 384-well plate followed by 5 µl of a 10 mM $FeCl_3$ solution (dissolved in water). The plates were then spectrophotometrically scanned between 350-900 nm with 20 nm steps. Controls consisted of compounds with water, DMSO with iron, and DMSO with water. Shifts in absorbance in the presence versus absence of iron were used to detect iron binding. The experiment was performed in triplicate with the line showing the mean value.

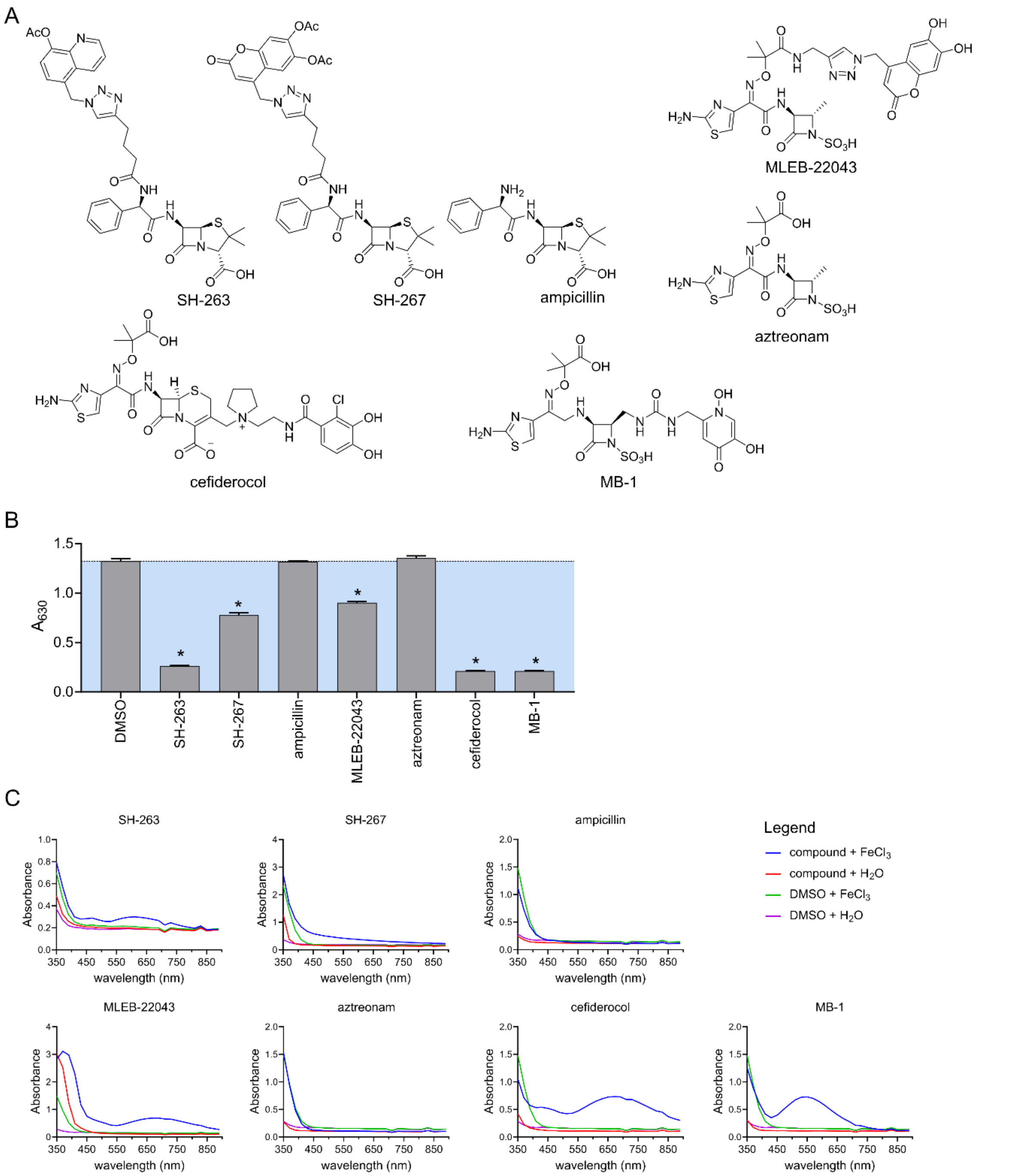

Figure S5. Iron-binding activity of siderophore-antibiotic conjugates. A) Structures of relevant antibiotic and sideorphore-antibiotic conjugates. B) Chrome Azurol S (CAS) assay data. Each compound was tested at a final concentration of 0.5mM in the presence of the CAS reagent. A reduction in absorbance compared to the DMSO control is considered positive for iron-binding.*-indicates significant difference ($p<0.05$) from the DMSO control as determined by one-way ANOVA and Dunnet's multiple comparisons test. The experiment was performed in triplicate and the graphed data show the mean ± standard deviation. C) UV-vis absorption spectra for indicated compounds. 5 µl of a 10 mM solution of each compound (dissolved in DMSO) was added to a 384-well plate followed by 5 µl of a 10 mM $FeCl_3$ solution (dissolved in water). The plates were then spectrophotometrically scanned between 350-900 nm with 20 nm steps. Controls consisted of compounds with water, DMSO with iron, and DMSO with water. Shifts in absorbance in the presence versus absence of iron were used to detect iron binding. The experiment was performed in triplicate with the line showing the mean value.

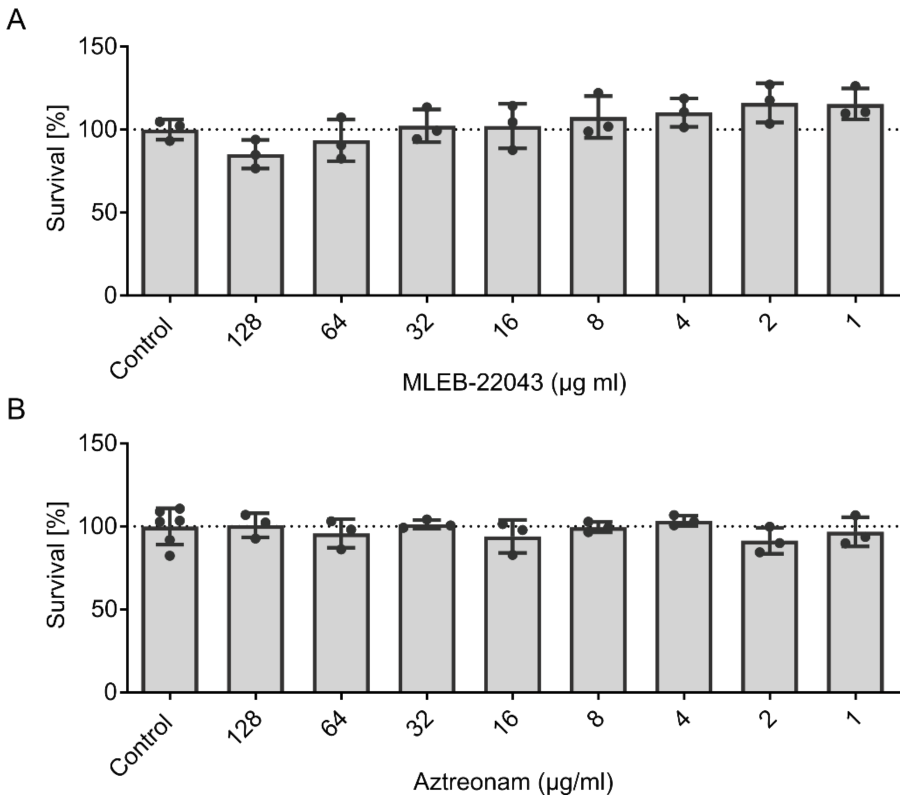

Figure S6. Cytotoxicity testing of MLEB-22043 against HEK 293 cells. HEK 293 cells were incubated with the indicated concentrations of A) MLEB-22043 or B) aztreonam for 24 hours and cell viability assessed using the PrestoBlue assay. The results are expressed as percent survival as compared to DMSO controls. The data is from 3 biological replicates and expressed as the mean ± standard deviation. No significant difference between treatment groups and control groups was seen for any compound at any concentration as assessed by a one-way ANOVA and Dunnett's multiple comparisons test.

Supplementary Tables

Table S3. MICs of MLEB-22043, cefiderocol, and aztreonam against *E. coli* BW25113 wild-type and Δ*tonB*

	MIC (μg/mL)					
	MLEB-22043		cefiderocol		aztreonam	
strain	MHB	ID-MHB	MHB	ID-MHB	MHB	ID-MHB
E. coli BW25113	0.5	0.12	0.06	0.008	0.12	0.12
E. coli BW25113 Δ*tonB*	16	ND[a]	2	ND[a]	0.12	ND[a]

[a]ND: not done; the *tonB* mutant does not grow in ID-MHB

Table S4. MICs of MLEB-22043, cefiderocol, and aztreonam against *P. aeruginosa* PA14 overexpressing TonB-dependent transporters

	MIC (μg/mL) in MHB + 1% arabinose		
P. aeruginosa PA14 overexpression strain	MLEB-22043	cefiderocol	aztreonam
pHERD20T (empty vector)	0.5	0.5	4
PA14_09340 (FptA)	1	0.5	4
PA14_09970 (FpvB)	1	0.5	4
PA14_33680 (FpvA)	1	0.5	4
PA14_63960	1	0.5	4
PA14_54180	1	0.5	4
PA14_30590	1	0.5	4
**PA14_58570 (PiuA)**	**<0.25**	**<0.25**	4
**PA14_52230 (PirA)**	**<0.25**	**<0.25**	4
PA14_05640	1	0.5	4
PA14_29350 (PfeA)	ND	ND	ND
PA14_39650 (CirA)	1	0.5	4
PA14_10200 (FvbA)	1	0.5	4
PA14_39820 (FemA)	1	0.5	4
PA14_61850 (ChtA)	1	0.5	4
PA14_37730	1	0.5	4
PA14_43650	1	0.5	4
PA14_06160 (FiuA)	1	0.5	4
PA14_32740 (FoxA)	1	0.5	4
PA14_46640 (AleB)	1	0.5	4
PA14_13430 (FecA)	1	0.5	4
PA14_21730	ND	ND	ND
PA14_34990	1	0.5	4
PA14_26420	1	0.5	4
PA14_01870	ND	ND	ND
**PA14_47140 (PfuA)**	**<0.25**	**<0.25**	4
PA14_55050	1	0.5	4
PA14_37490	1	0.5	4
PA14_37900	1	0.5	4
PA14_20010 (HasR)	1	0.5	4
PA14_47380 (HxuC)	ND	ND	ND
PA14_62350 (PhuR)	1	0.5	4
PA14_64710	1	0.5	4
PA14_15070 (OprC)	ND	ND	ND
PA14_47800 (BtuB)	ND	ND	ND
PA14_02410	1	0.5	4

[a]ND: not done; overexpression inhibited growth

Table S5. MICs of MLEB-22043, cefiderocol, and aztreonam against *P. aeruginosa* PA14 TBDT mutant strains

	MIC (µg/mL), MHB[a]		
strain	MLEB-22043	cefiderocol	aztreonam
P. aeruginosa PA14	0.5	0.5	8
P. aeruginosa PA14 Δ*piuA*	**2**	**4**	8
P. aeruginosa PA14 Δ*pirA*	1	0.5	8
P. aeruginosa PA14 Δ*pfuA*	0.5	0.5	8
P. aeruginosa PA14 Δ*piuApirA*	**>8**	**8**	8
P. aeruginosa PA14 Δ*piuApirA* pHERD20T	**>8**	**>8**	4
P. aeruginosa PA14 Δ*piuApirA* pHERD20T-*piuA*	0.5	0.5	4
P. aeruginosa PA14 Δ*piuApirA* pHERD20T-*pirA*	0.5	**2**	4

[a]bold values indicate ≥4-fold change in MIC from the wildtype strain

Table S6. MICs of MLEB-22043 and aztreonam against *P. aeruginosa* PA14 *tonB* mutants

	P. aeruginosa PA14		*P. aeruginosa* PA14 Δ*tonB1*		*P. aeruginosa* PA14 Δ*tonB2*		*P. aeruginosa* PA14 Δ*tonB3*	
	MIC (µg/mL)		MIC (µg/mL)		MIC (µg/mL)		MIC (µg/mL)	
compound	MHB	ID	MHB	ID	MHB	ID	MHB	ID
MLEB-22043	1	0.25	1	0.125	2	0.25	1	0.125
aztreonam	8	4	16	8	8	4	8	4

Table S7. MICs of MLEB-22043 and aztreonam against efflux-deficient *P. aeruginosa* mPAO1

	P. aeruginosa mPAO1		*P. aeruginosa* mPAO1 Δ*opmD,* Δ*oprJ,* Δ*oprM*	
	MIC (µg/mL)		MIC (µg/mL)	
compound	MHB	ID-MHB	MHB	ID-MHB
MLEB-22043	2	0.125	1	0.125
aztreonam	2	1	0.25	0.125

Table S8. Activity of MLEB-22043 and other β-lactam antibiotics against *K. pneumoniae* and *P. aeruginosa* strains

		MIC (µg/mL)[a]							
Strain	Predicted β-lactamases[b]	043[c]	043+AVI[c]	ATM[d]	ATM+AVI[d]	CAZ[e]	CAZ+AVI[e]	CFDC[f]	MER[g]
K. pneumoniae C0612	SHV-1, CTX-M-14, NDM-1, CTX-M-15, OXA-48	2	0.06	**>16**	0.25	**>16**	**>16**	0.12	**>16**
K. pneumoniae C0650	SHV-27, NDM-1, CTX-M-15, OXA-232, OXA-9, TEM-55, CMY-23	**8**	0.25	**>16**	0.5	**>16**	**>16**	0.5	**>16**
P. aeruginosa C0292	PDC-5, OXA-50	0.12	<0.03	**>16**	**>16**	**>16**	**16**	<0.03	**>16**
P. aeruginosa C0334	OXA-486, PDC-7	8	0.12	**>16**	**16**	**>16**	**16**	0.25	**>16**
P. aeruginosa C0177	OXA-488, PDC-2	0.12	<0.03	**>16**	**16**	**16**	8	<0.03	**>16**
P. aeruginosa C0029	OXA-488, PDC-2	0.5	0.06	**>16**	**16**	**>16**	8	<0.03	**8**
P. aeruginosa C0410	OXA-488, PDC-2	<0.03	<0.03	**>16**	**>16**	4	4	<0.03	**>16**
P. aeruginosa C0028	PDC-3	<0.03	<0.03	**16**	**16**	4	4	<0.03	**>16**
P. aeruginosa C0263	PDC-1, OXA-50	<0.03	<0.03	**16**	**16**	4	4	<0.03	**>16**
P. aeruginosa C0293	PDC-5, OXA-50	0.12	0.12	**16**	**16**	4	4	0.25	**>16**
P. aeruginosa C0070	OXA-486, PDC-3,	<0.03	<0.03	8	8	4	4	<0.03	**>16**
P. aeruginosa C0089	OXA-50, PDC-7	0.06	<0.03	8	8	8	2	<0.03	**8**
P. aeruginosa PAO1	PDC-1	0.06	0.06	8	8	2	2	<0.03	2

[a]bold values indicate non-susceptible based on CLSI breakpoints [1]. Aztreonam breakpoints used as surrogate for MLEB-22043
[b]predictions from genome sequence by CARD [2]

[c]043: MLEB-22043, AVI: avibactam (at 4 μg/mL); tested in ID-MHB
[d]ATM: aztreonam, AVI: avibactam (at 4 μg/mL)
[e]CAZ: ceftazidime, AVI: avibactam (at 4 μg/mL)
[f]CFDC: cefiderocol; tested in ID-MHB
[g]MER: meropenem

Table S9. MICs of SH-263, SH-267, and ampicillin in combination with avibactam against *K. pneumoniae*, *E. coli*, and *P. aeruginosa*

	K. pneumoniae MKP103		*E. coli* BW25113		*K. pneumoniae* ATCC 43816		*P. aeruginosa* PAO1	
	MIC (μg/mL)		MIC (μg/mL)		MIC (μg/mL)		MIC (μg/mL)	
compound	ID	ID+AVI	ID	ID+AVI	ID	ID+AVI	ID	ID+AVI
SH-263	>32	>32	4	4	8	2	>32	32
SH-267	>32	4	1	1	2	1	8	1
ampicillin	>32	16	8	4	16	1	>32	>32

Table S10. Bacterial strain list

Strain	Note	Source or reference
K. pneumoniae MKP103	*Δkpc-3*	[3]
K. pneumoniae KPNIH1	parent strain for MKP103	[3,4]
K. pneumoniae ATCC 43816		ATCC
P. aeruginosa PAO1		Laboratory strain; Dr. Jon Dennis
E. coli BW25113		[5]
E. coli BW25113 Δ*tonB*		[5]
P. aeruginosa PA14		[6]
P. aeruginosa PA14 Δ*piuA*		This work
P. aeruginosa PA14 Δ*pirA*		This work
P. aeruginosa PA14 Δ*PA14_47140*		This work
P. aeruginosa PA14 Δ*piuApirA*		This work
P. aeruginosa PA14 Δ*piuApirA* pHERD20T	vector control strain	This work
P. aeruginosa PA14 Δ*piuApirA* pHERD20T-*piuA*	PiuA overexpression strain	This work
P. aeruginosa PA14 Δ*piuApirA* pHERD20T-*pirA*	PirA overexpression strain	This work
P. aeruginosa PA14 Δ*tonB1*	*tonB1*::Mar2xT7	[6]
P. aeruginosa PA14 Δ*tonB2*		This work
P. aeruginosa PA14 Δ*tonB3*		This work
P. aeruginosa PAO1 ARC545	wildtype	[7]
P. aeruginosa PAO1 ARC4736	Δ[PiuC(PA4515)…PA4521]	[7]
P. aeruginosa PAO1 ARC6046	p*fecI* (-44 T→C)	[7]
P. aeruginosa PAO1 ARC4238	Δ*piuA*	[7]
P. aeruginosa PAO1 ARC5406	Δ*piuC*	[7]
P. aeruginosa PAO1 ARC4239	Δ*pirA*	[7]
P. aeruginosa PAO1 ARC4242	Δ*piuApirA*	[7]
P. aeruginosa mPAO1		[8]
P. aeruginosa mPAO1 Δ*opmDoprJM*	Efflux-deficient strain	[9]
E. coli Δ*bamB*Δ*tolC*	Host strain for β-lactamase overexpression	[10]
K. pneumoniae C0026		Clinical Isolate, McMaster University
K. pneumoniae C0612		Clinical Isolate, McMaster University
K. pneumoniae C0650		Clinical Isolate, McMaster University
E. coli C0005		Clinical Isolate, McMaster University
A. baumannii AB5075		[11]
A. baumannii ATCC 17978		Reference strain, ATCC
A. baumannii ATCC 19606		Reference strain, ATCC
A. baumannii C0015		Clinical Isolate, McMaster University
A. baumannii C0044		Clinical Isolate, McMaster University
A. baumannii C0074		Clinical Isolate, McMaster University

A. baumannii C0092		Clinical Isolate, McMaster University
Pseudomonas aeruginosa C0007		Clinical Isolate, McMaster University
Pseudomonas aeruginosa C0028		Clinical Isolate, McMaster University
Pseudomonas aeruginosa C0029		Clinical Isolate, McMaster University
Pseudomonas aeruginosa C0030		Clinical Isolate, McMaster University
Pseudomonas aeruginosa C0031		Clinical Isolate, McMaster University
Pseudomonas aeruginosa C0042		Clinical Isolate, McMaster University
Pseudomonas aeruginosa C0043		Clinical Isolate, McMaster University
Pseudomonas aeruginosa C0053		Clinical Isolate, McMaster University
Pseudomonas aeruginosa C0056		Clinical Isolate, McMaster University
Pseudomonas aeruginosa C0057		Clinical Isolate, McMaster University
Pseudomonas aeruginosa C0060		Clinical Isolate, McMaster University
Pseudomonas aeruginosa C0062		Clinical Isolate, McMaster University
Pseudomonas aeruginosa C0070		Clinical Isolate, McMaster University
Pseudomonas aeruginosa C0071		Clinical Isolate, McMaster University
Pseudomonas aeruginosa C0072		Clinical Isolate, McMaster University
Pseudomonas aeruginosa C0073		Clinical Isolate, McMaster University
Pseudomonas aeruginosa C0080		Clinical Isolate, McMaster University
Pseudomonas aeruginosa C0089		Clinical Isolate, McMaster University
Pseudomonas aeruginosa C0090		Clinical Isolate, McMaster University
Pseudomonas aeruginosa C0091		Clinical Isolate, McMaster University
Pseudomonas aeruginosa C0098		Clinical Isolate, McMaster University
Pseudomonas aeruginosa C0099		Clinical Isolate, McMaster University
Pseudomonas aeruginosa C0100		Clinical Isolate, McMaster University
Pseudomonas aeruginosa C0129		Clinical Isolate, McMaster University
Pseudomonas aeruginosa C0138		Clinical Isolate, McMaster University
Pseudomonas aeruginosa C0139		Clinical Isolate, McMaster University
Pseudomonas aeruginosa C0148		Clinical Isolate, McMaster University
Pseudomonas aeruginosa C0155		Clinical Isolate, McMaster University
Pseudomonas aeruginosa C0156		Clinical Isolate, McMaster University
Pseudomonas aeruginosa C0157		Clinical Isolate, McMaster University
Pseudomonas aeruginosa C0166		Clinical Isolate, McMaster University
Pseudomonas aeruginosa C0167		Clinical Isolate, McMaster University
Pseudomonas aeruginosa C0168		Clinical Isolate, McMaster University
Pseudomonas aeruginosa C0176		Clinical Isolate, McMaster University
Pseudomonas aeruginosa C0177		Clinical Isolate, McMaster University
Pseudomonas aeruginosa C0178		Clinical Isolate, McMaster University
Pseudomonas aeruginosa C0179		Clinical Isolate, McMaster University
Pseudomonas aeruginosa C0189		Clinical Isolate, McMaster University
Pseudomonas aeruginosa C0190		Clinical Isolate, McMaster University
Pseudomonas aeruginosa C0200		Clinical Isolate, McMaster University
Pseudomonas aeruginosa C0201		Clinical Isolate, McMaster University
Pseudomonas aeruginosa C0202		Clinical Isolate, McMaster University
Pseudomonas aeruginosa C0203		Clinical Isolate, McMaster University
Pseudomonas aeruginosa C0204		Clinical Isolate, McMaster University
Pseudomonas aeruginosa C0213		Clinical Isolate, McMaster University
Pseudomonas aeruginosa C0214		Clinical Isolate, McMaster University
Pseudomonas aeruginosa C0215		Clinical Isolate, McMaster University
Pseudomonas aeruginosa C0222		Clinical Isolate, McMaster University
Pseudomonas aeruginosa C0223		Clinical Isolate, McMaster University
Pseudomonas aeruginosa C0224		Clinical Isolate, McMaster University
Pseudomonas aeruginosa C0225		Clinical Isolate, McMaster University
Pseudomonas aeruginosa C0231		Clinical Isolate, McMaster University
Pseudomonas aeruginosa C0232		Clinical Isolate, McMaster University
Pseudomonas aeruginosa C0233		Clinical Isolate, McMaster University
Pseudomonas aeruginosa C0234		Clinical Isolate, McMaster University
Pseudomonas aeruginosa C0245		Clinical Isolate, McMaster University
Pseudomonas aeruginosa C0254		Clinical Isolate, McMaster University
Pseudomonas aeruginosa C0260		Clinical Isolate, McMaster University
Pseudomonas aeruginosa C0261		Clinical Isolate, McMaster University
Pseudomonas aeruginosa C0262		Clinical Isolate, McMaster University
Pseudomonas aeruginosa C0263		Clinical Isolate, McMaster University
Pseudomonas aeruginosa C0274		Clinical Isolate, McMaster University
Pseudomonas aeruginosa C0275		Clinical Isolate, McMaster University
Pseudomonas aeruginosa C0276		Clinical Isolate, McMaster University
Pseudomonas aeruginosa C0284		Clinical Isolate, McMaster University

Pseudomonas aeruginosa C0292		Clinical Isolate, McMaster University
Pseudomonas aeruginosa C0293		Clinical Isolate, McMaster University
Pseudomonas aeruginosa C0294		Clinical Isolate, McMaster University
Pseudomonas aeruginosa C0295		Clinical Isolate, McMaster University
Pseudomonas aeruginosa C0296		Clinical Isolate, McMaster University
Pseudomonas aeruginosa C0305		Clinical Isolate, McMaster University
Pseudomonas aeruginosa C0306		Clinical Isolate, McMaster University
Pseudomonas aeruginosa C0307		Clinical Isolate, McMaster University
Pseudomonas aeruginosa C0315		Clinical Isolate, McMaster University
Pseudomonas aeruginosa C0332		Clinical Isolate, McMaster University
Pseudomonas aeruginosa C0333		Clinical Isolate, McMaster University
Pseudomonas aeruginosa C0334		Clinical Isolate, McMaster University
Pseudomonas aeruginosa C0335		Clinical Isolate, McMaster University
Pseudomonas aeruginosa C0344		Clinical Isolate, McMaster University
Pseudomonas aeruginosa C0345		Clinical Isolate, McMaster University
Pseudomonas aeruginosa C0346		Clinical Isolate, McMaster University
Pseudomonas aeruginosa C0355		Clinical Isolate, McMaster University
Pseudomonas aeruginosa C0356		Clinical Isolate, McMaster University
Pseudomonas aeruginosa C0365		Clinical Isolate, McMaster University
Pseudomonas aeruginosa C0366		Clinical Isolate, McMaster University
Pseudomonas aeruginosa C0376		Clinical Isolate, McMaster University
Pseudomonas aeruginosa C0377		Clinical Isolate, McMaster University
Pseudomonas aeruginosa C0378		Clinical Isolate, McMaster University
Pseudomonas aeruginosa C0379		Clinical Isolate, McMaster University
Pseudomonas aeruginosa C0398		Clinical Isolate, McMaster University
Pseudomonas aeruginosa C0399		Clinical Isolate, McMaster University
Pseudomonas aeruginosa C0400		Clinical Isolate, McMaster University
Pseudomonas aeruginosa C0401		Clinical Isolate, McMaster University
Pseudomonas aeruginosa C0409		Clinical Isolate, McMaster University
Pseudomonas aeruginosa C0410		Clinical Isolate, McMaster University

Supporting Dataset- Compound NMR

5-(chloromethyl)quinolin-8-ol (**S1**) (^{1}H NMR; 400 MHz; DMSO-d_6)

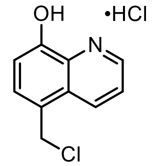

S1

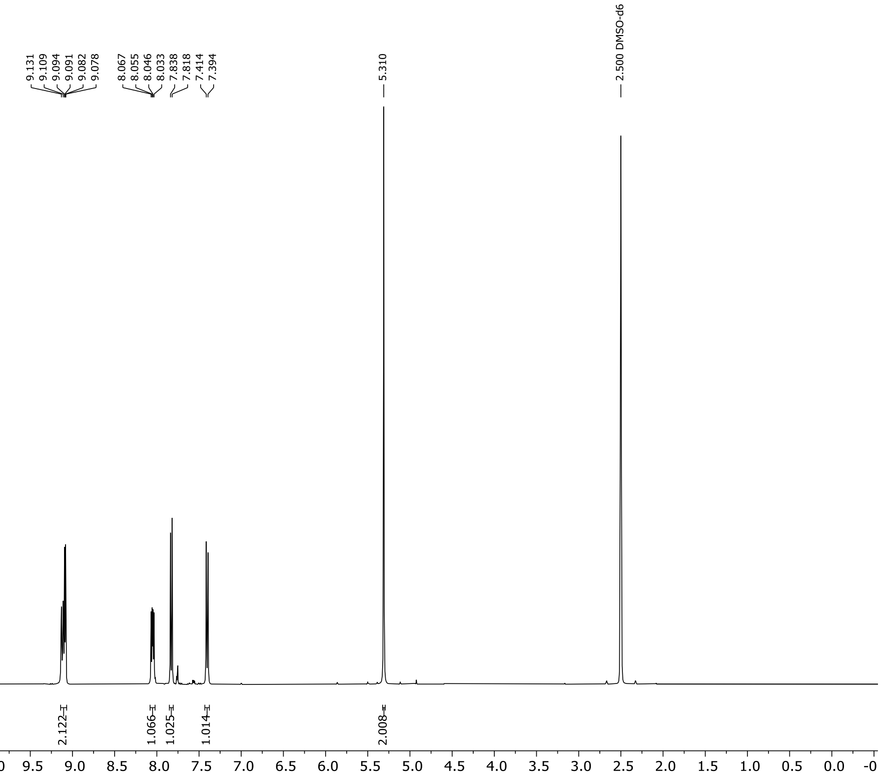

5-(chloromethyl)quinolin-8-ol (**S1**) (^{13}C NMR; 101 MHz; DMSO-d_6)

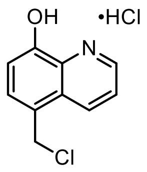

S1

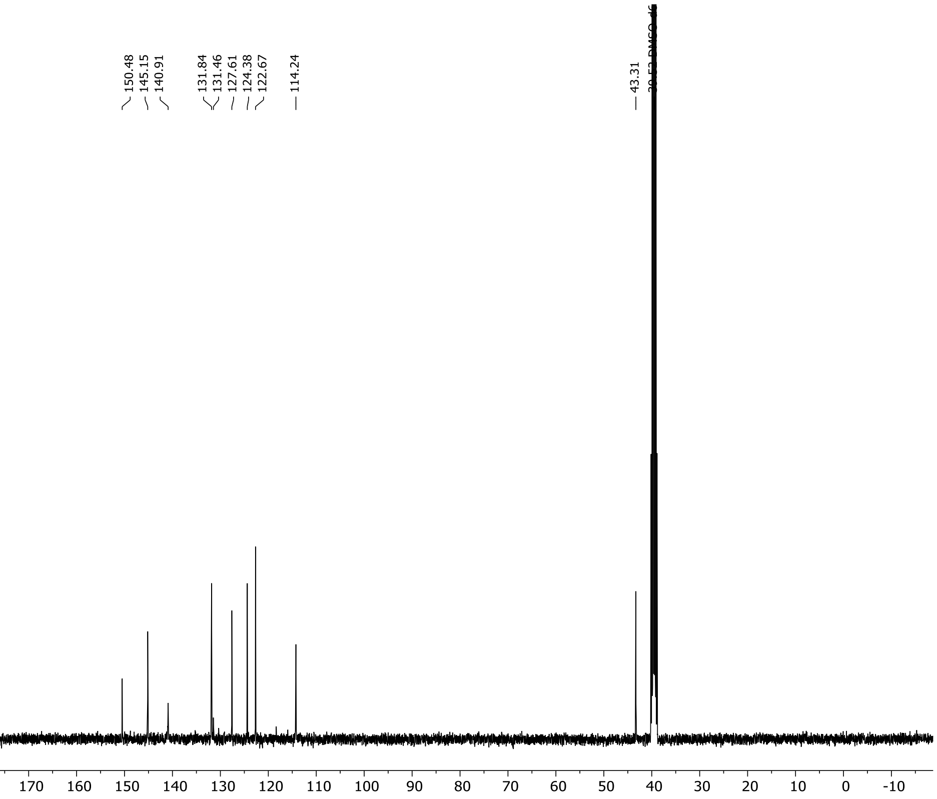

5-(azidomethyl)quinolin-8-ol (**S2**) (^{1}H NMR; 400 MHz; DMSO-d_6)

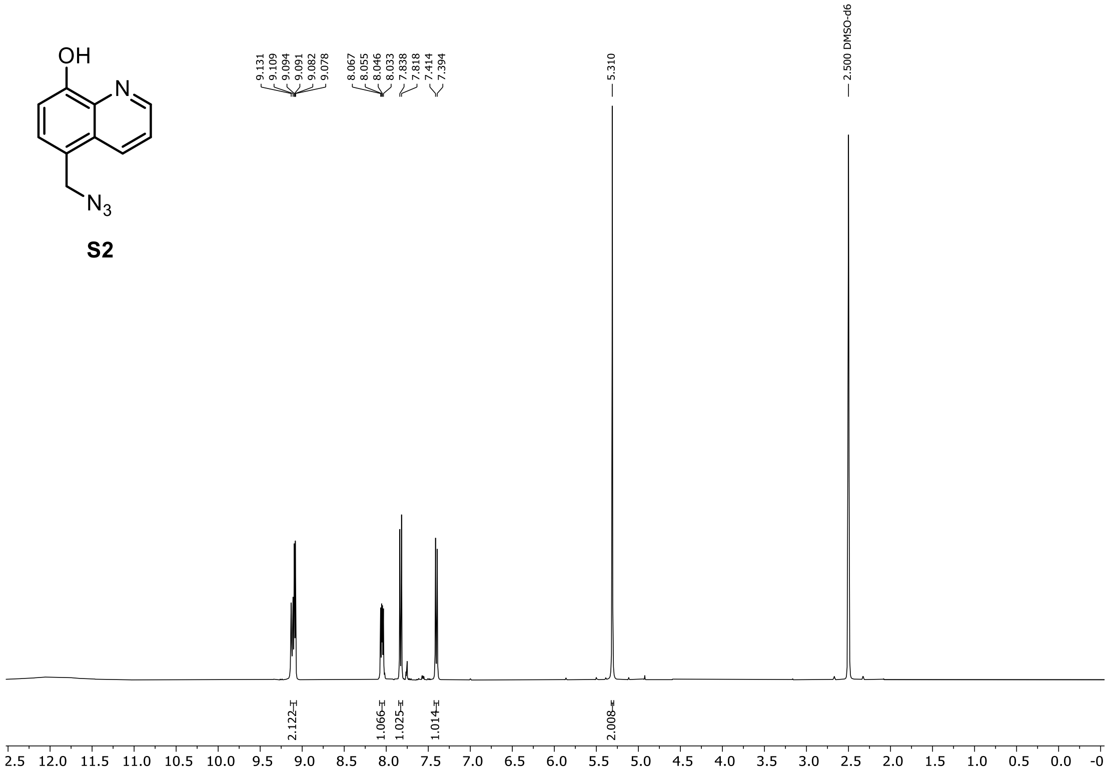

5-(azidomethyl)quinolin-8-ol (**S2**) (^{13}C NMR; 101 MHz; DMSO-d_6)

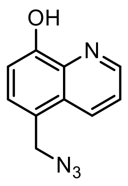

S2

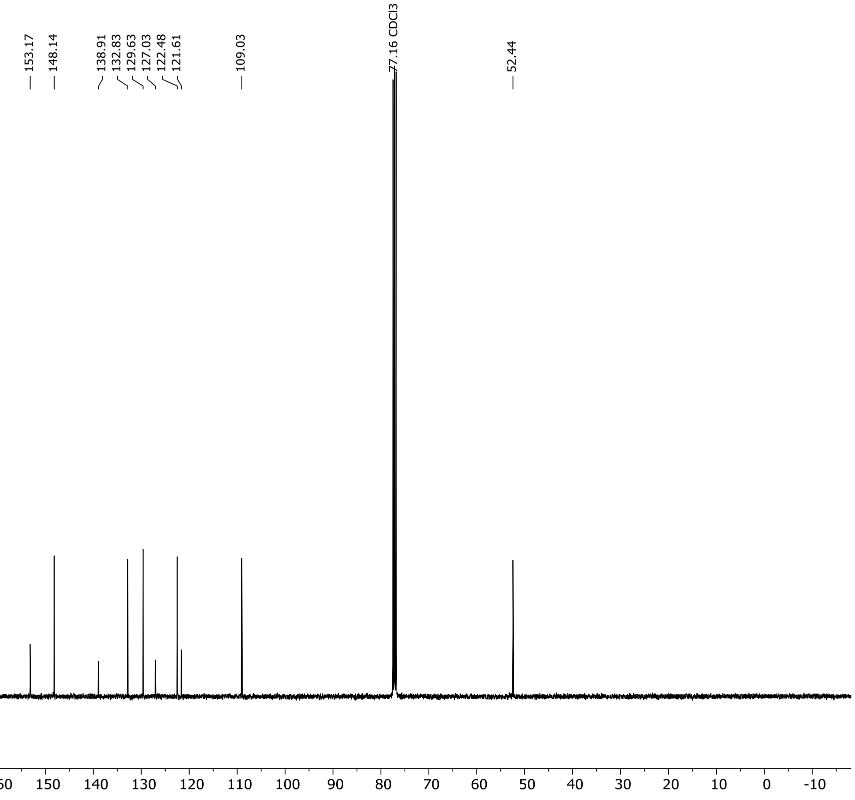

^{1}H and ^{13}C NMR Spectra

Compound name (**S3**) (^{1}H NMR; 400 MHz; DMSO-d_6)

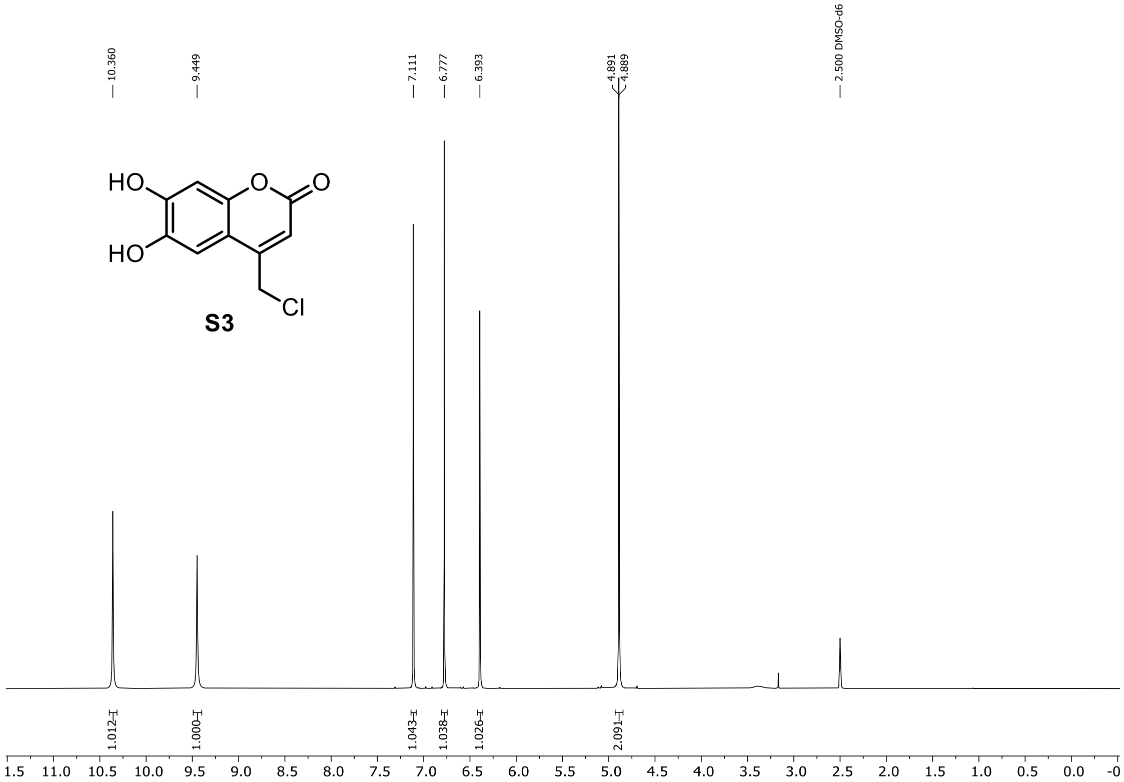

Compound name (**S3**) (^{13}C NMR; 101 MHz; DMSO-d_6)

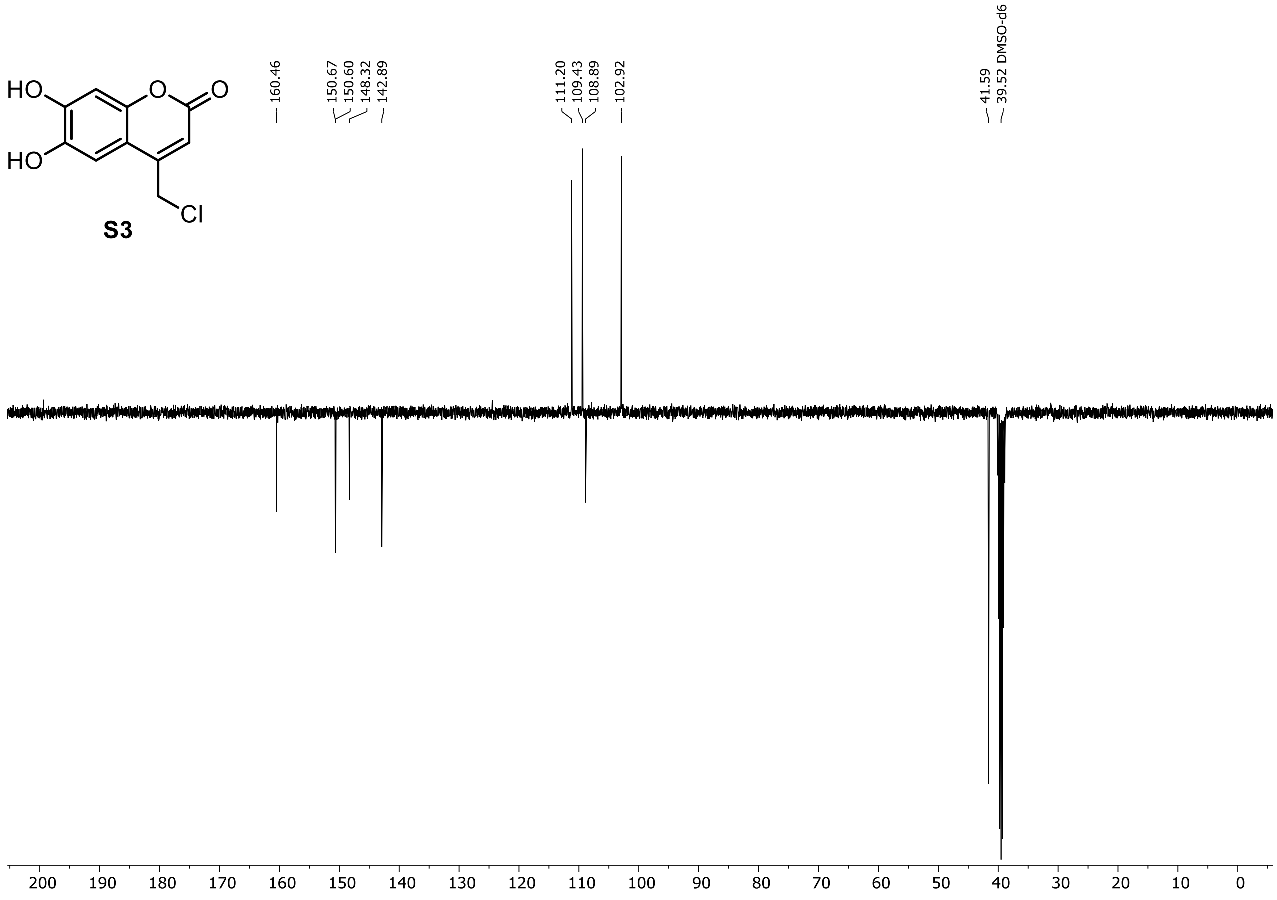

Compound name (**S4**) (^{1}H NMR; 400 MHz; DMSO-d_6)

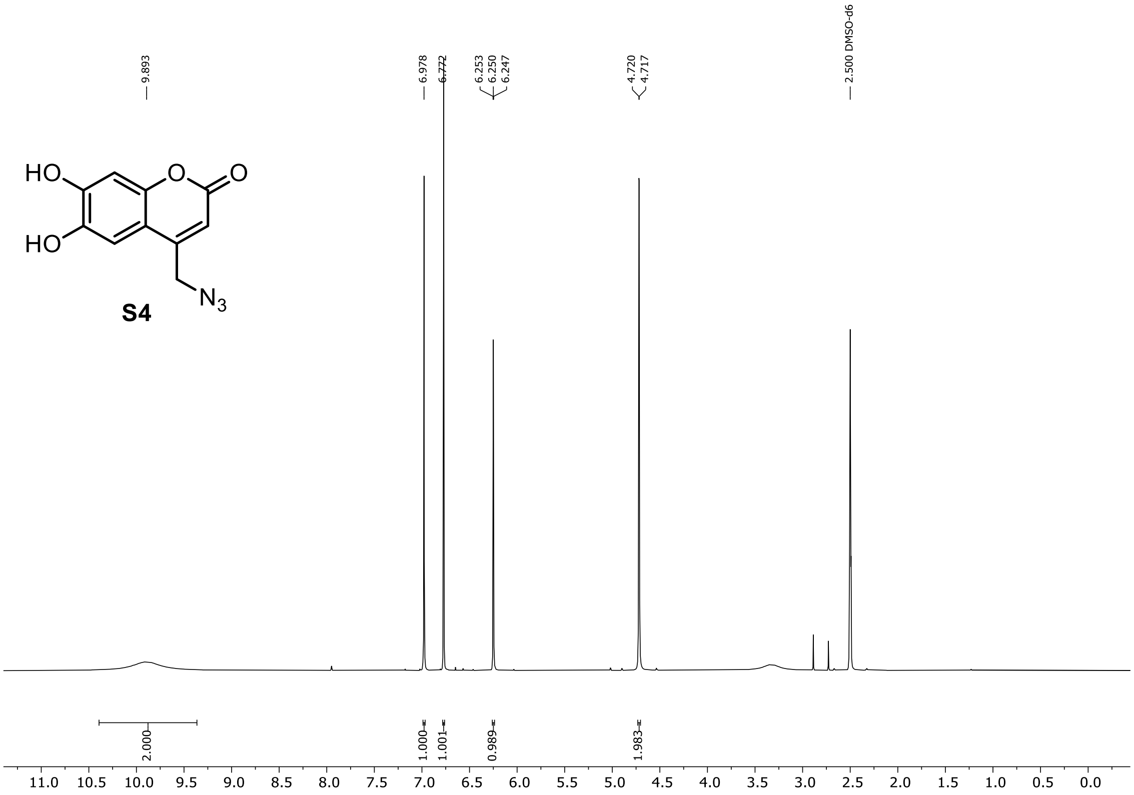

Compound name (**S4**) (^{13}C NMR; 101 MHz; DMSO-d_6)

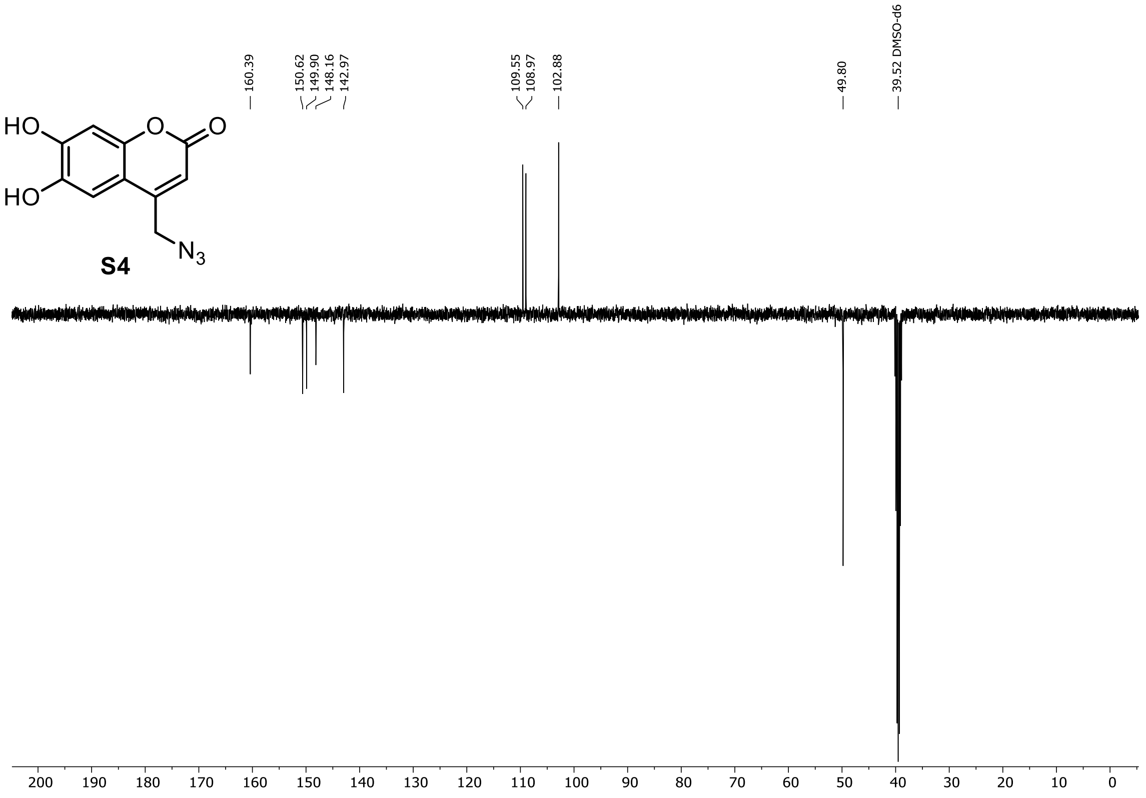

5-(Azidomethyl)quinolin-8-yl acetate (**S5**):

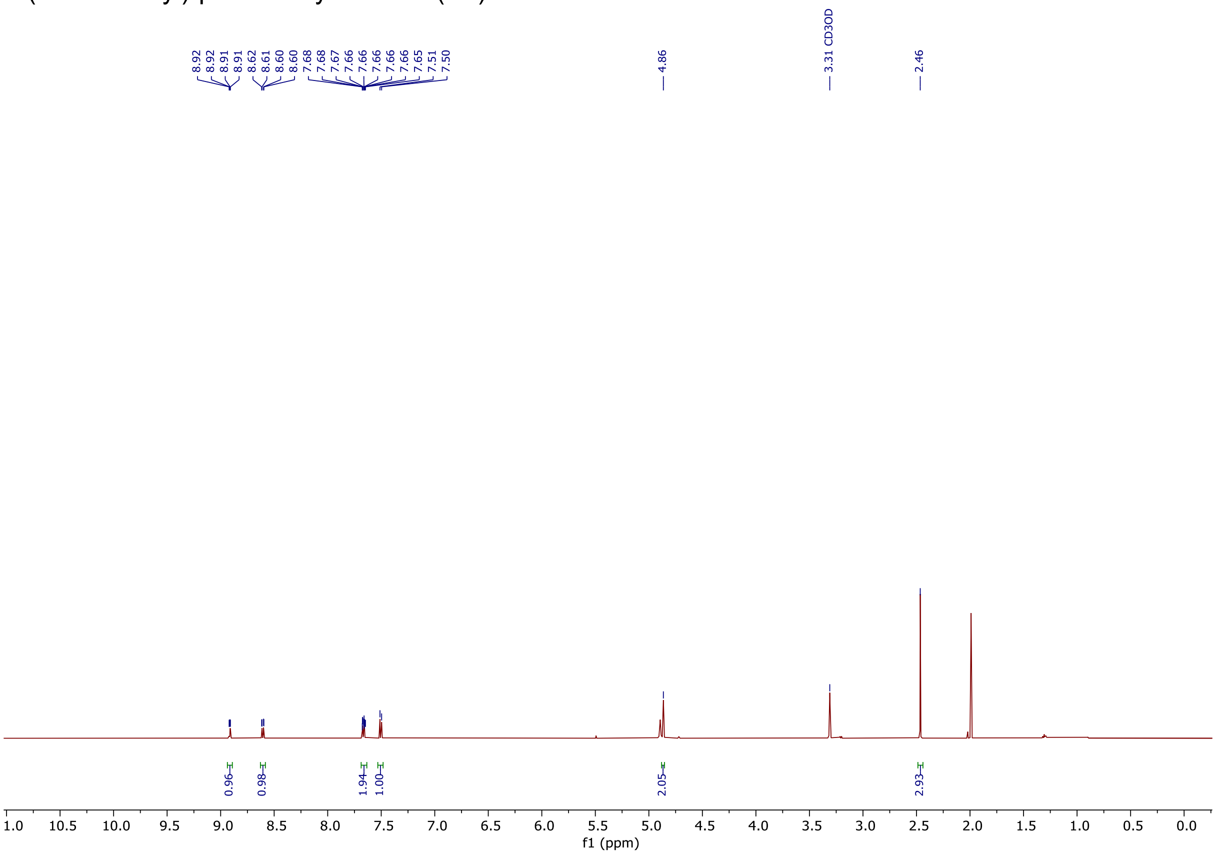

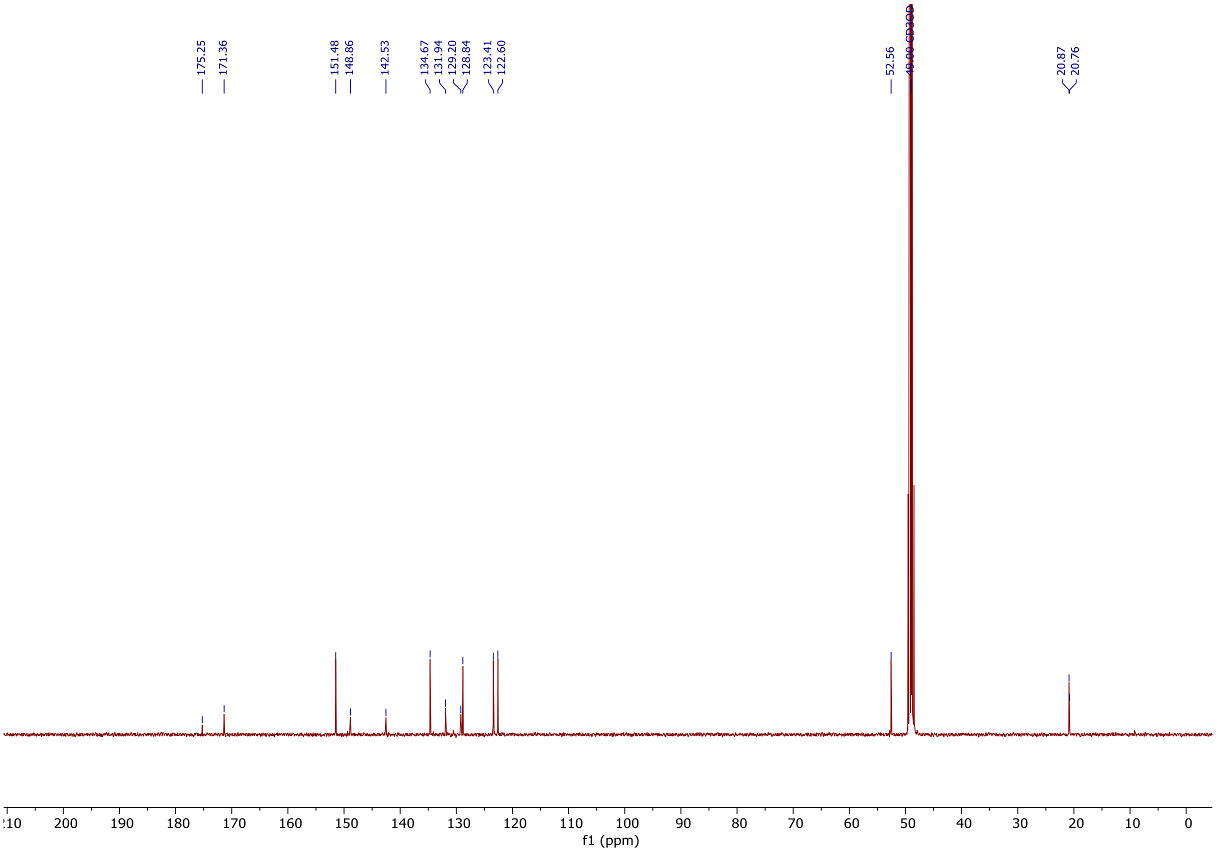
175.25
171.36
151.48
148.86
142.53
134.67
131.94
129.20
128.84
123.41
122.60
52.56
49.00 CD3OD
20.87
20.76
f1 (ppm)

4-(1-((8-acetoxyquinolin-5-yl)methyl)-1H-1,2,3-triazol-4-yl)butanoic acid (**S6**):

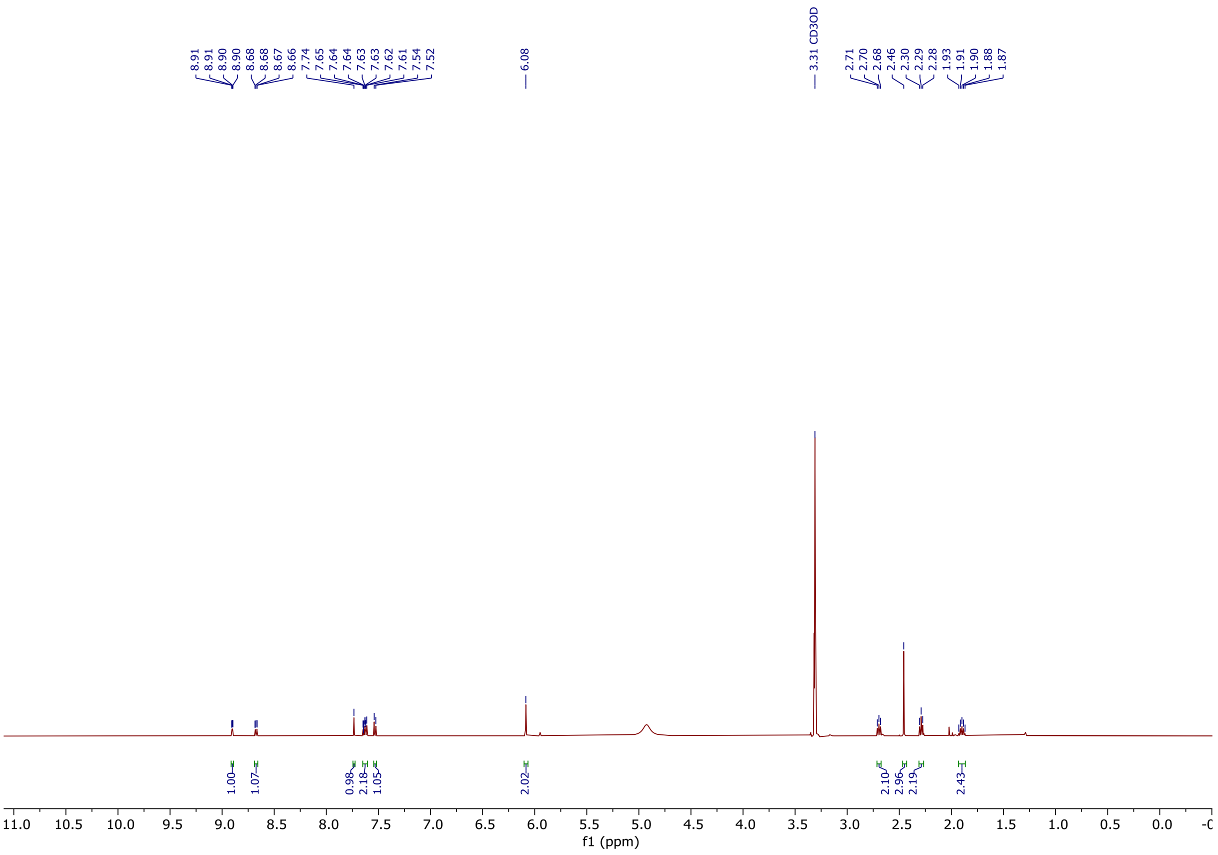

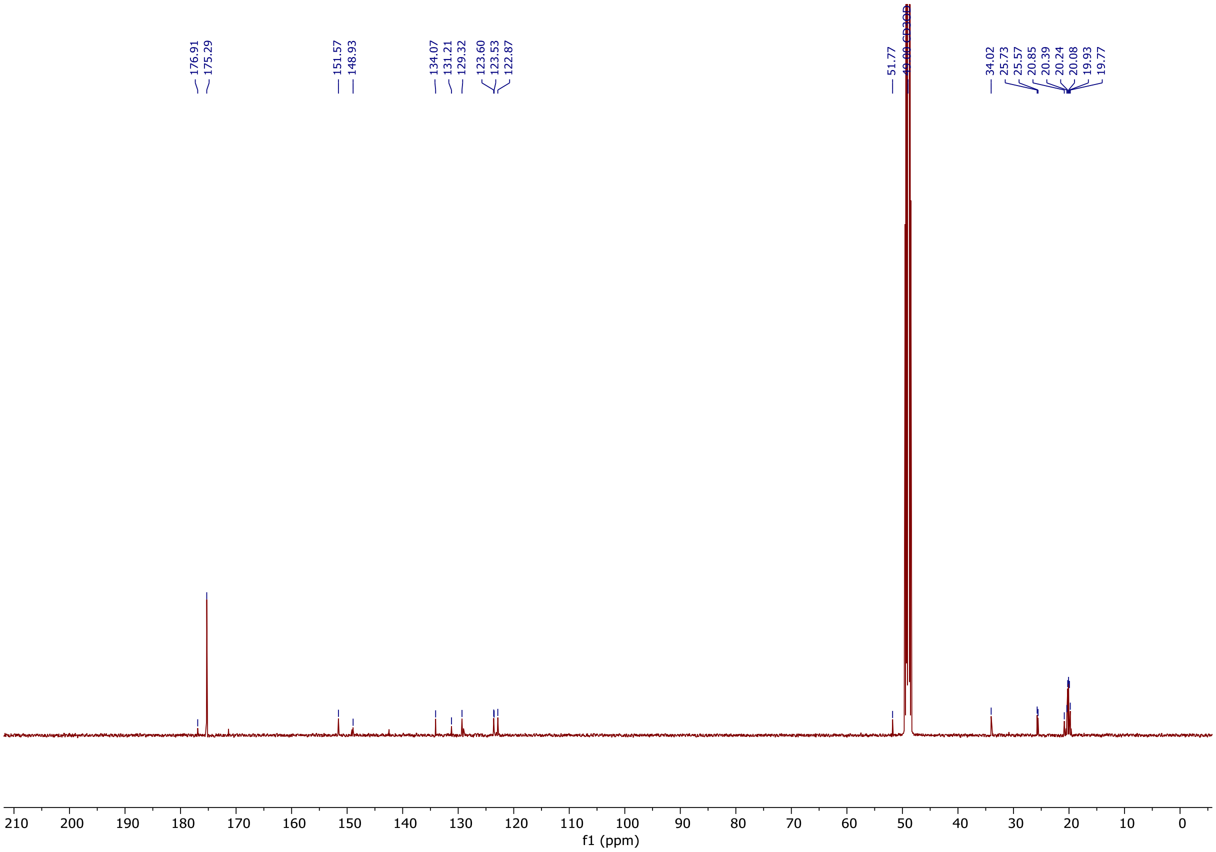
176.91
175.29
151.57
148.93
134.07
131.21
129.32
123.60
123.53
122.87
51.77
49.00 CD3OD
34.02
25.73
25.57
20.85
20.39
20.24
20.08
19.93
19.77
210 200 190 180 170 160 150 140 130 120 110 100 90 80 70 60 50 40 30 20 10 0
f1 (ppm)

(2*S*,5*R*,6*S*)-6-((*R*)-2-(4-(1-((8-acetoxyquinolin-5-yl)methyl)-1H-1,2,3-triazol-4-yl)butanamido)-2-phenylacetamido)-3,3-dimethyl-7-oxo-4-thia-1-azabicyclo[3.2.0]heptane-2-carboxylic acid (**SH-263**):

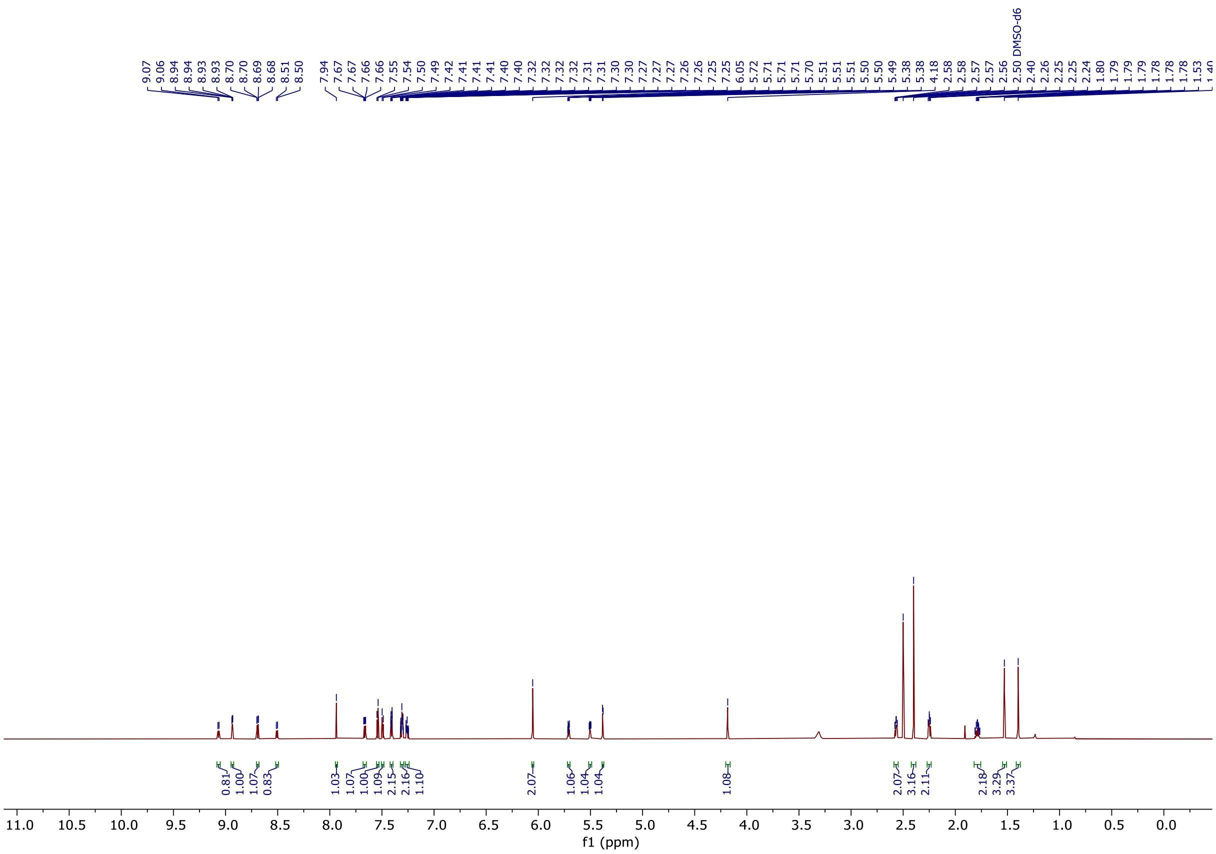

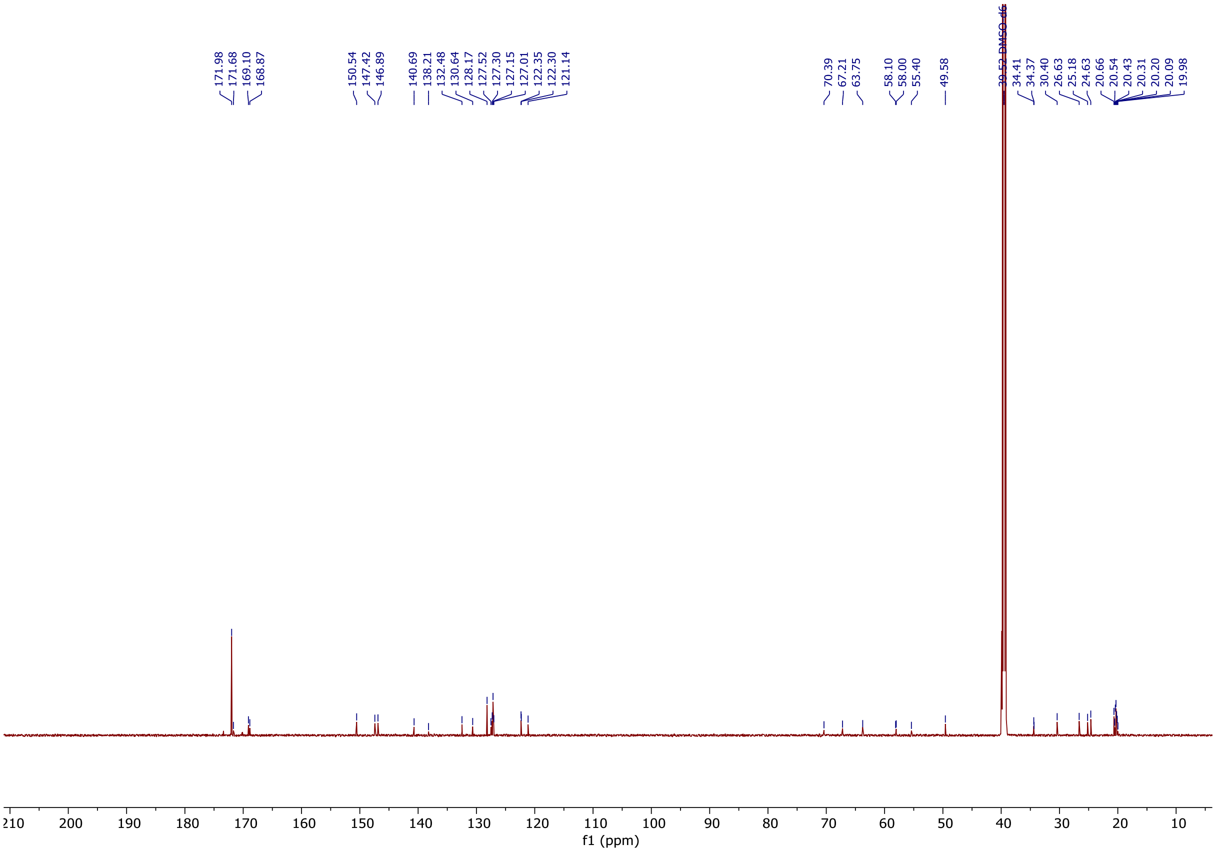

171.98
171.68
169.10
168.87
150.54
147.42
146.89
140.69
138.21
132.48
130.64
128.17
127.52
127.30
127.15
127.01
122.35
122.30
121.14
70.39
67.21
63.75
58.10
58.00
55.40
49.58
DMSO-d6
34.41
34.37
30.40
26.63
25.18
24.63
20.66
20.54
20.43
20.31
20.20
20.09
19.98
210 200 190 180 170 160 150 140 130 120 110 100 90 80 70 60 50 40 30 20 10
f1 (ppm)

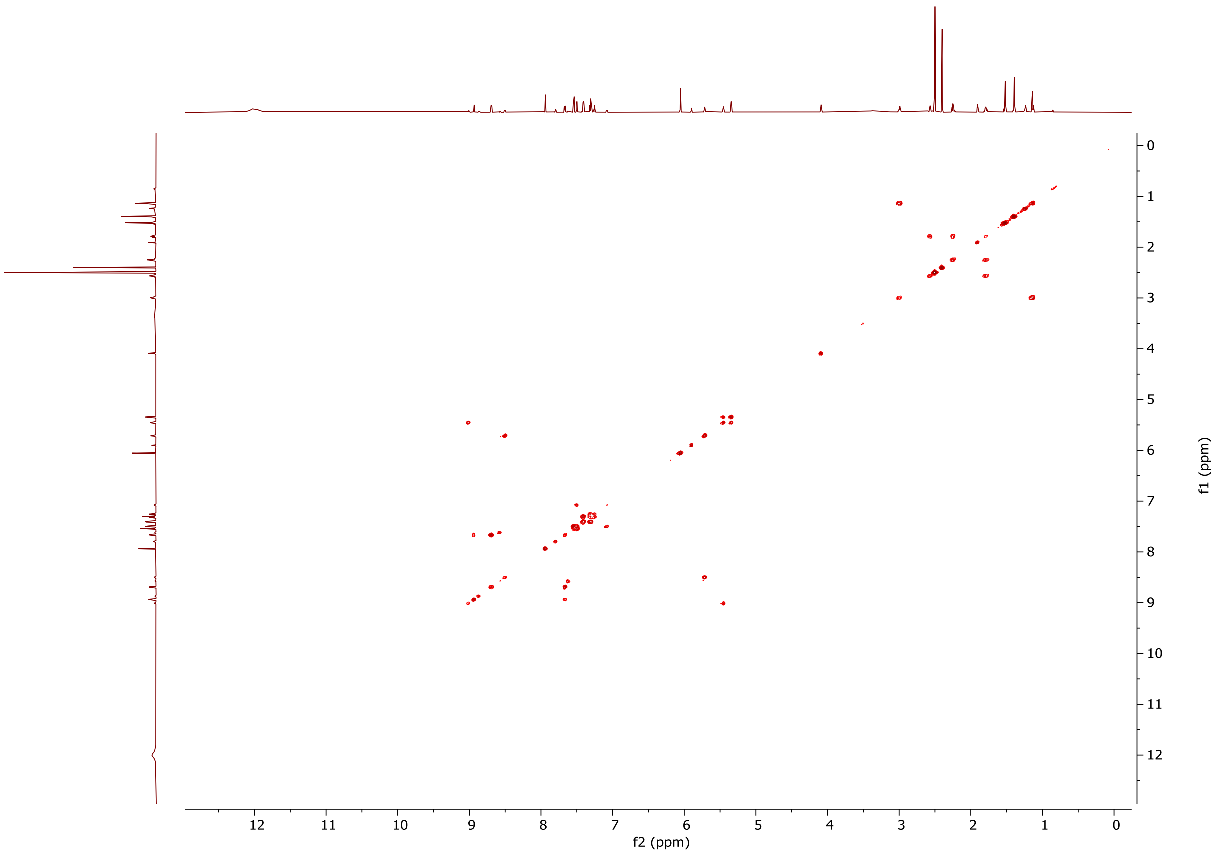

12 11 10 9 8 7 6 5 4 3 2 1 0
f2 (ppm)
0 1 2 3 4 5 6 7 8 9 10 11 12
f1 (ppm)

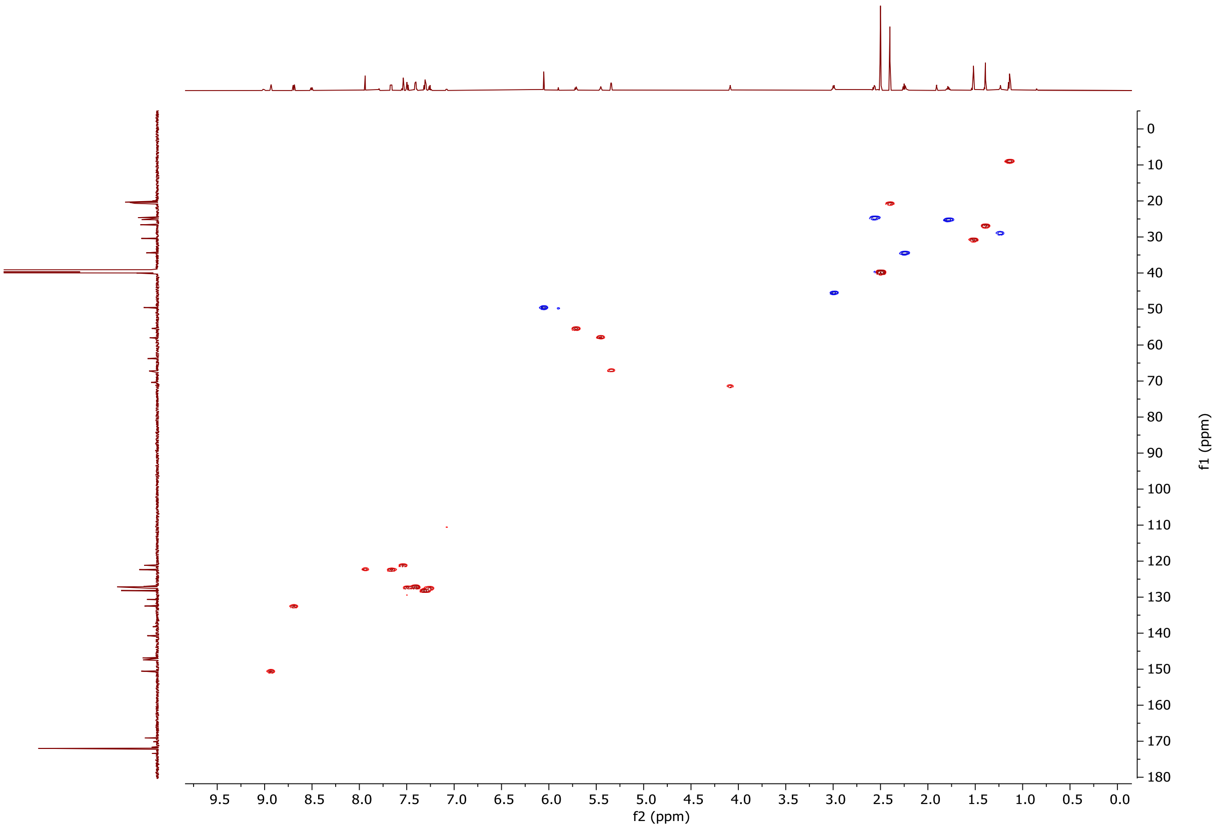
f1 (ppm)
f2 (ppm)

4-(Azidomethyl)-2-oxo-2H-chromene-6,7-diyl diacetate (**S7**):

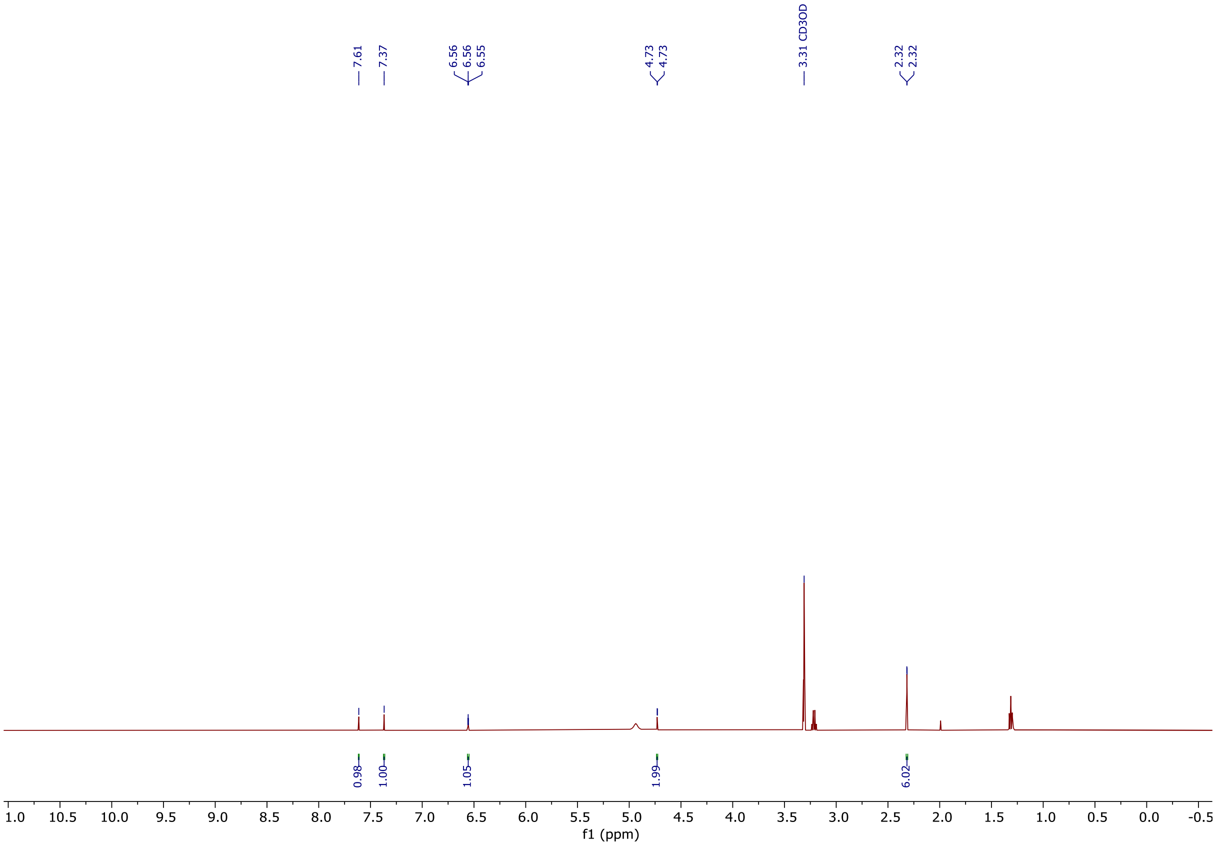

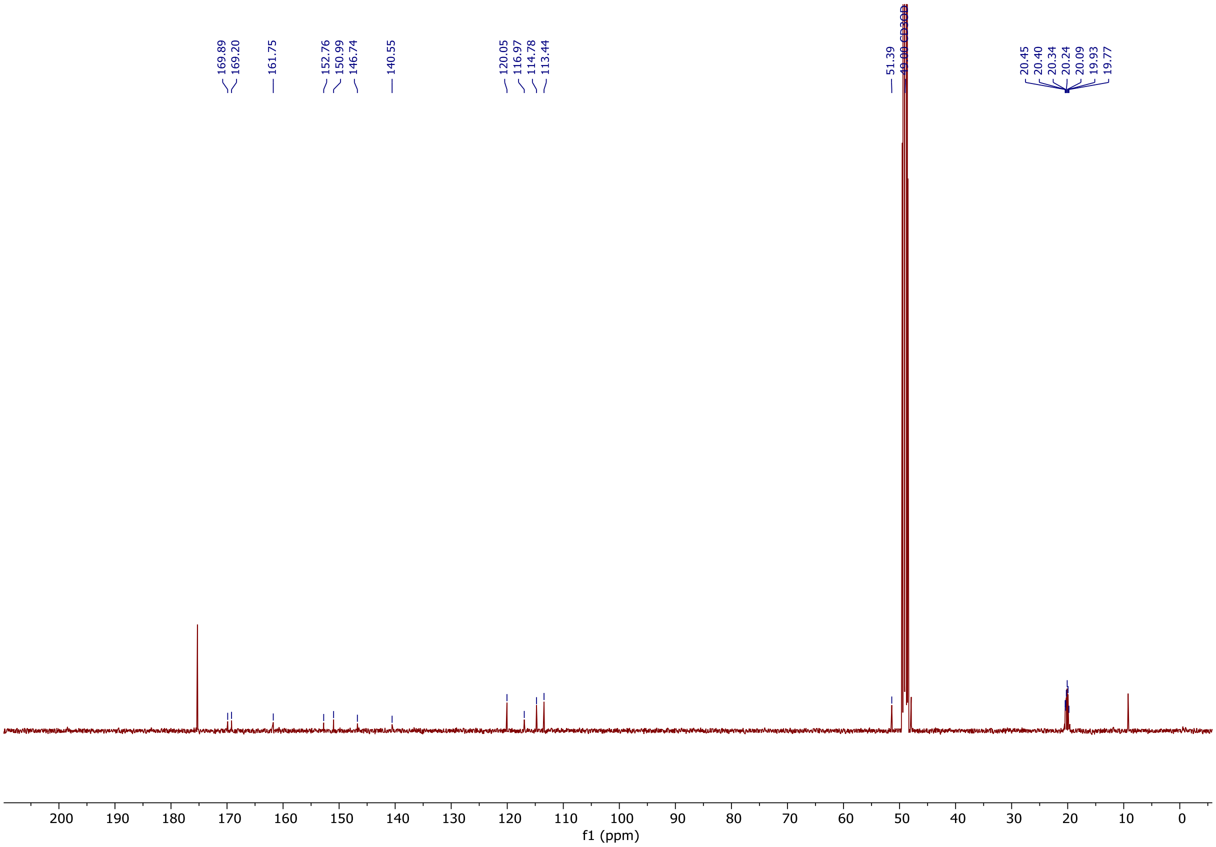
169.89
169.20
161.75
152.76
150.99
146.74
140.55
120.05
116.97
114.78
113.44
51.39
49.00 CD3OD
20.45
20.40
20.34
20.24
20.09
19.93
19.77
200 190 180 170 160 150 140 130 120 110 100 90 80 70 60 50 40 30 20 10 0
f1 (ppm)

4-(1-((6,7-diacetoxy-2-oxo-2H-chromen-4-yl)methyl)-1H-1,2,3-triazol-4-yl)butanoic acid (**S8**):

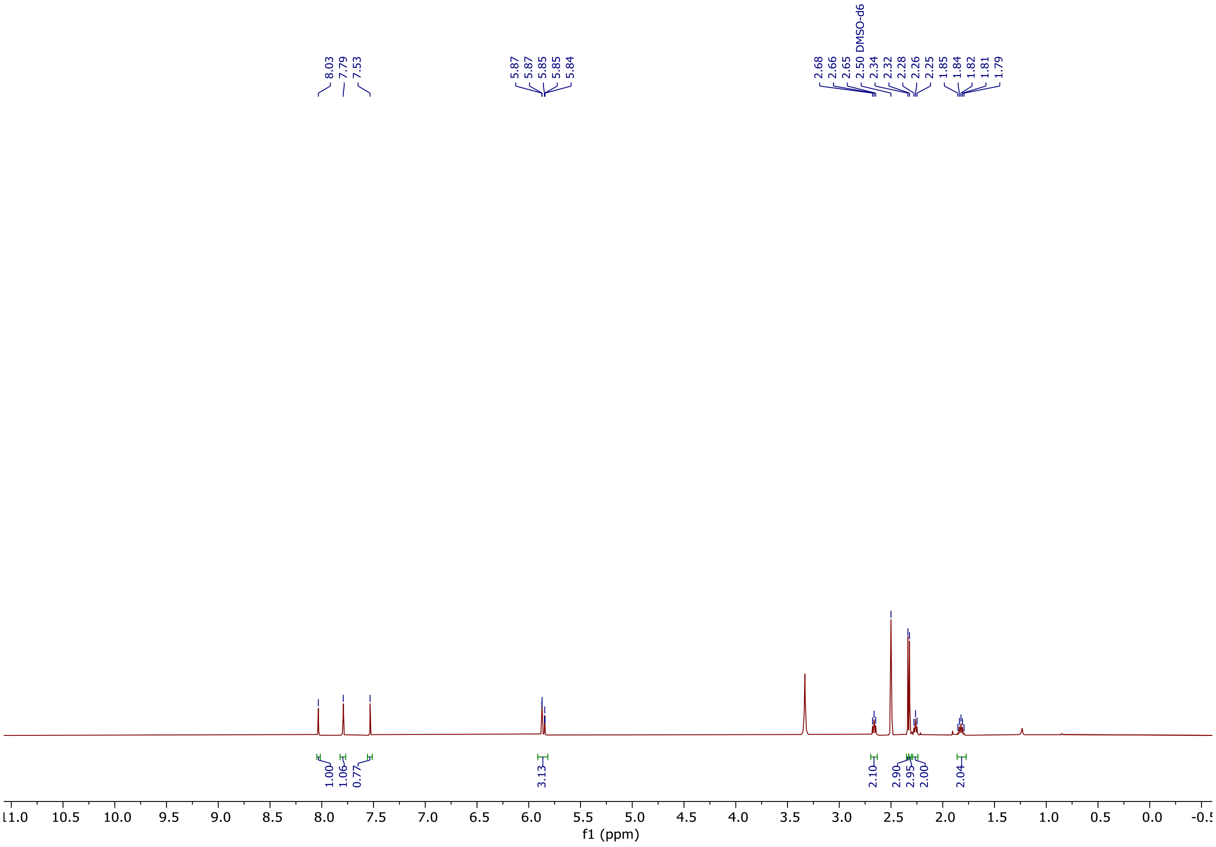

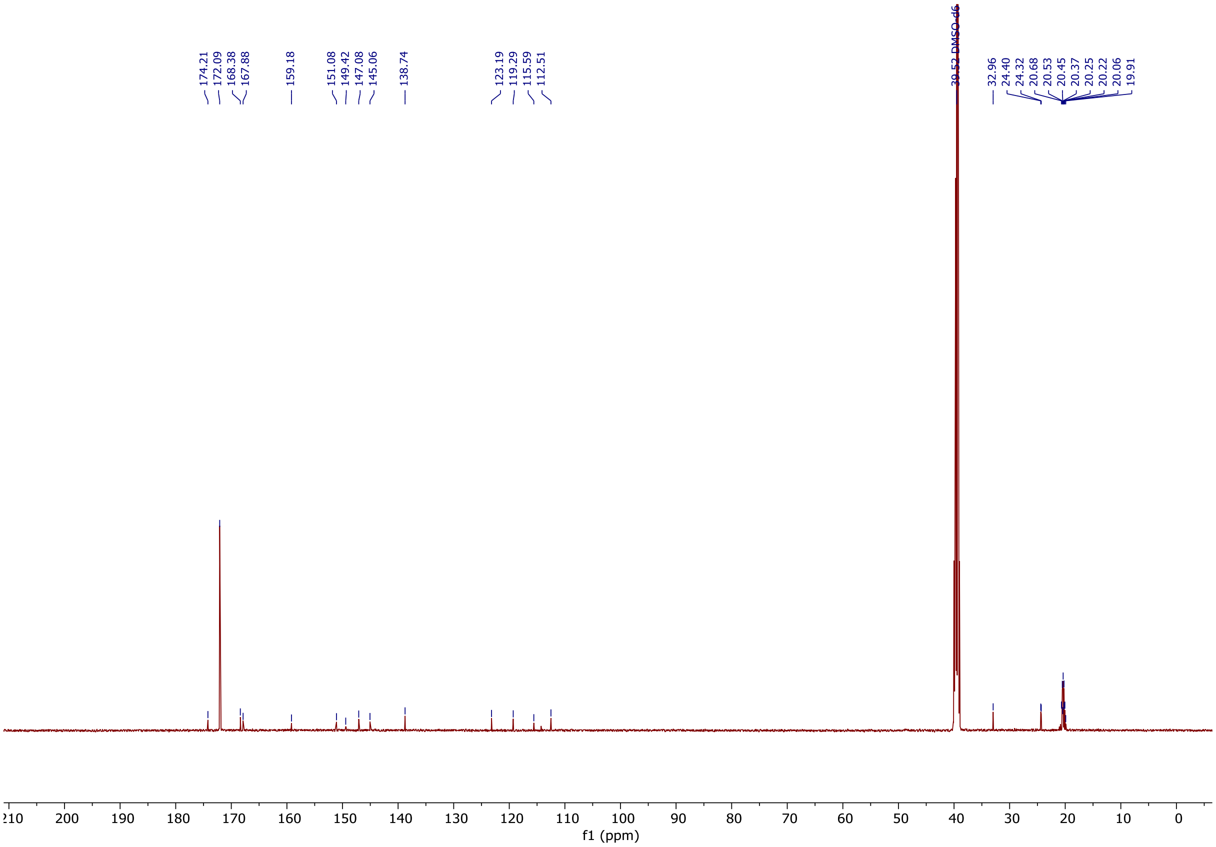

174.21
172.09
168.38
167.88
159.18
151.08
149.42
147.08
145.06
138.74
123.19
119.29
115.59
112.51
39.52 DMSO-d6
32.96
24.40
24.32
20.68
20.53
20.45
20.37
20.25
20.22
20.06
19.91
f1 (ppm)

(2*S*,5*R*,6*R*)-6-((*R*)-2-(4-(1-((6,7-diacetoxy-2-oxo-2H-chromen-4-yl)methyl)-1H-1,2,3-triazol-4-yl)butanamido)-2-phenylacetamido)-3,3-dimethyl-7-oxo-4-thia-1-azabicyclo[3.2.0]heptane-2-carboxylic acid (**SH-267**):

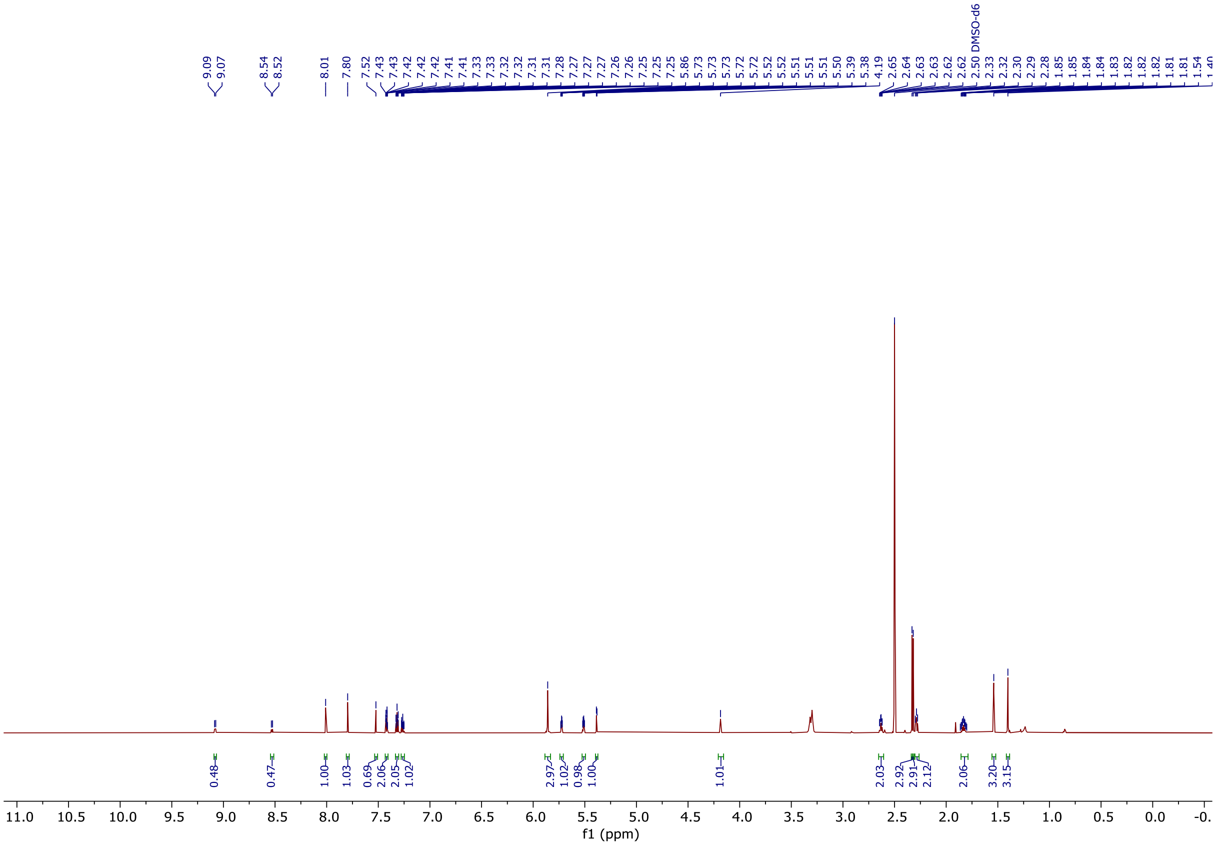

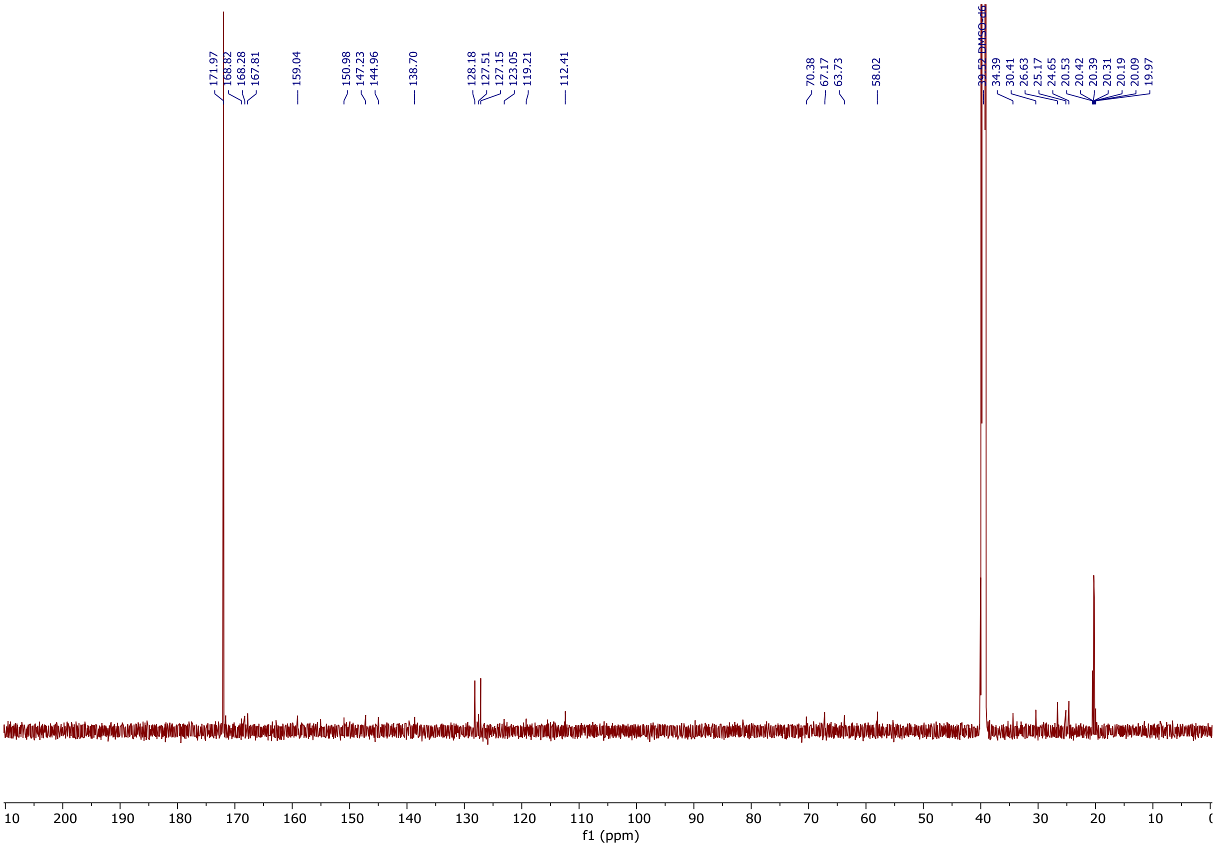
171.97
168.82
168.28
167.81
159.04
150.98
147.23
144.96
138.70
128.18
127.51
127.15
123.05
119.21
112.41
70.38
67.17
63.73
58.02
39.52 DMSO-d6
34.39
30.41
26.63
25.17
24.65
20.53
20.42
20.39
20.31
20.19
20.09
19.97
f1 (ppm)

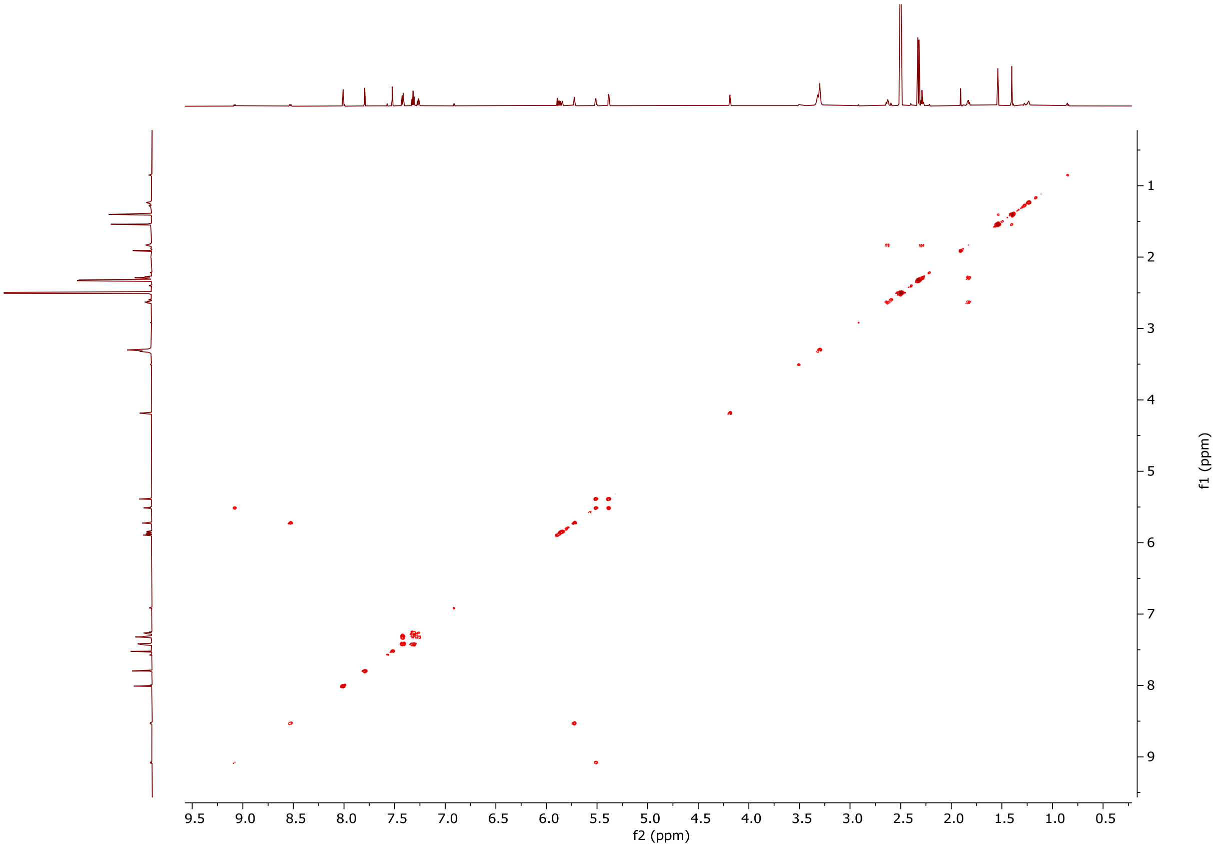
f2 (ppm)
f1 (ppm)

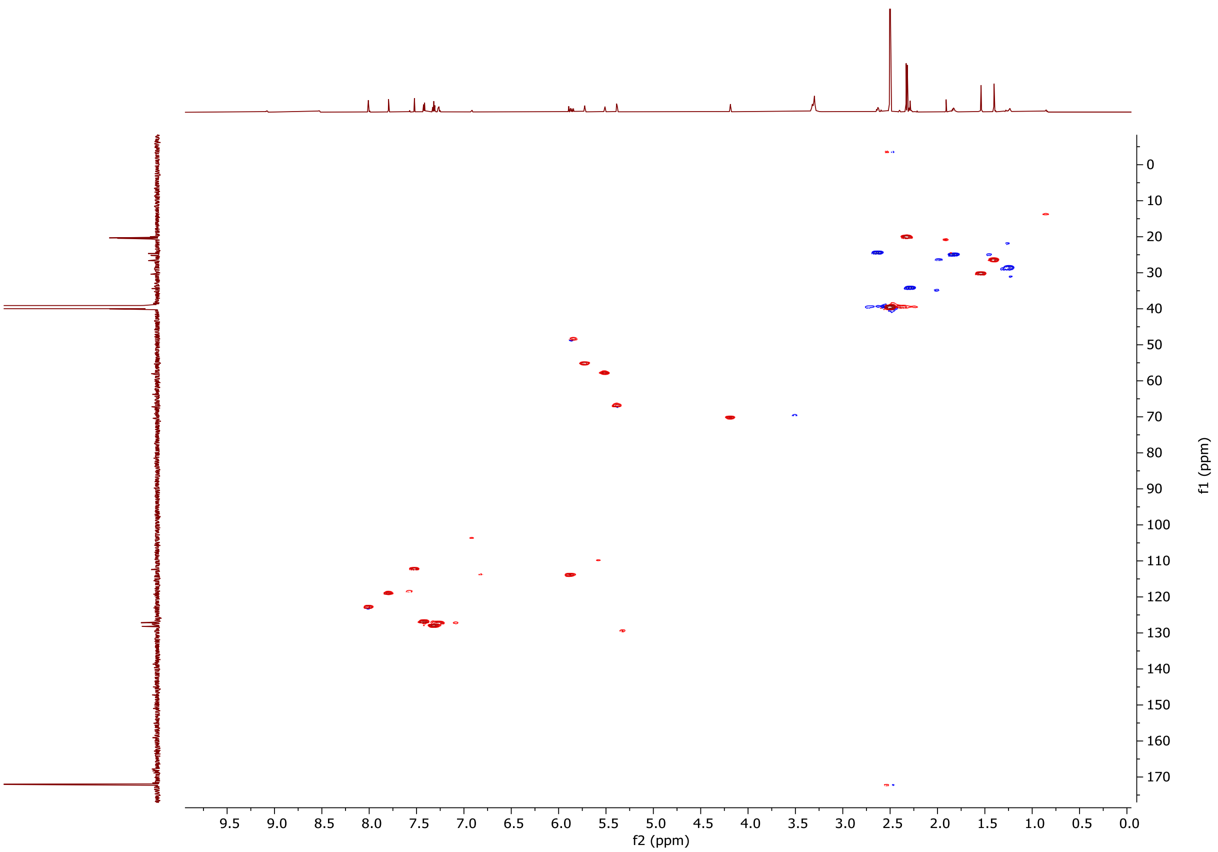
f1 (ppm)
f2 (ppm)

Aztreonam-Alkyne (**S9**) (^{1}H NMR; 400 MHz; DMSO-d_6)

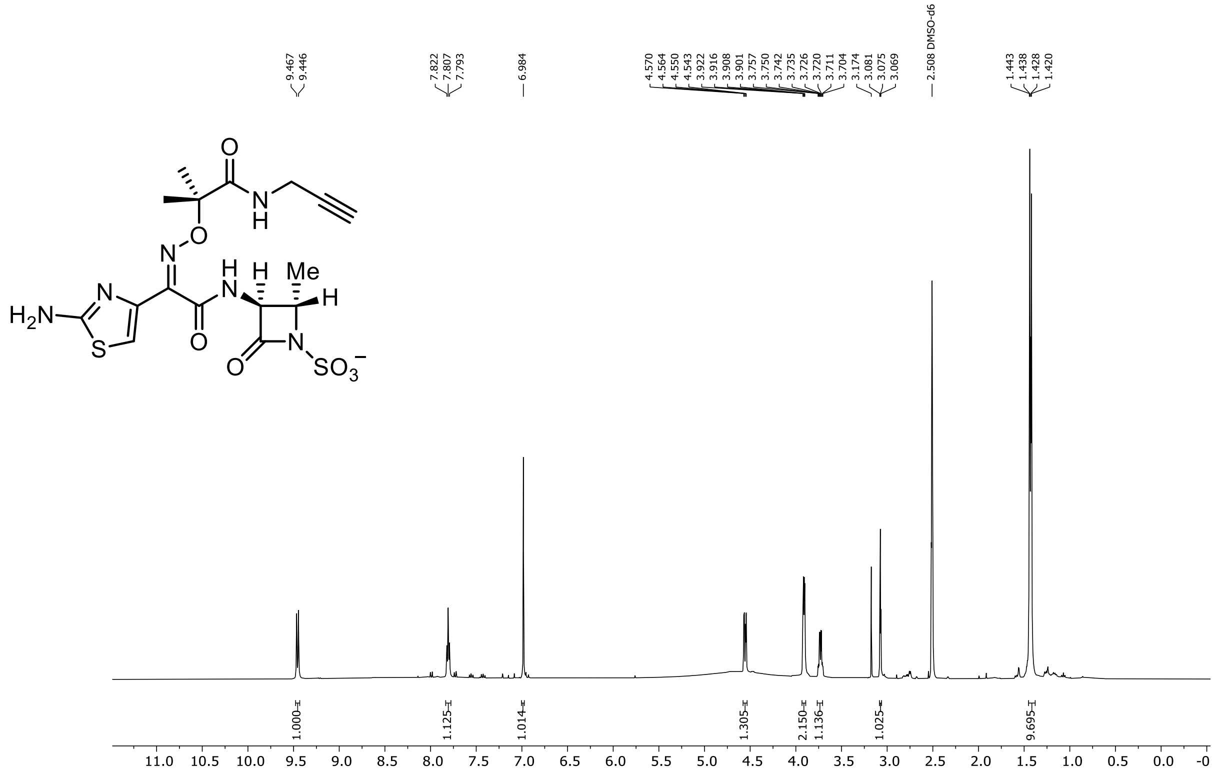

MLEB-22043 (^{1}H NMR; 400 MHz; DMSO-d_6)

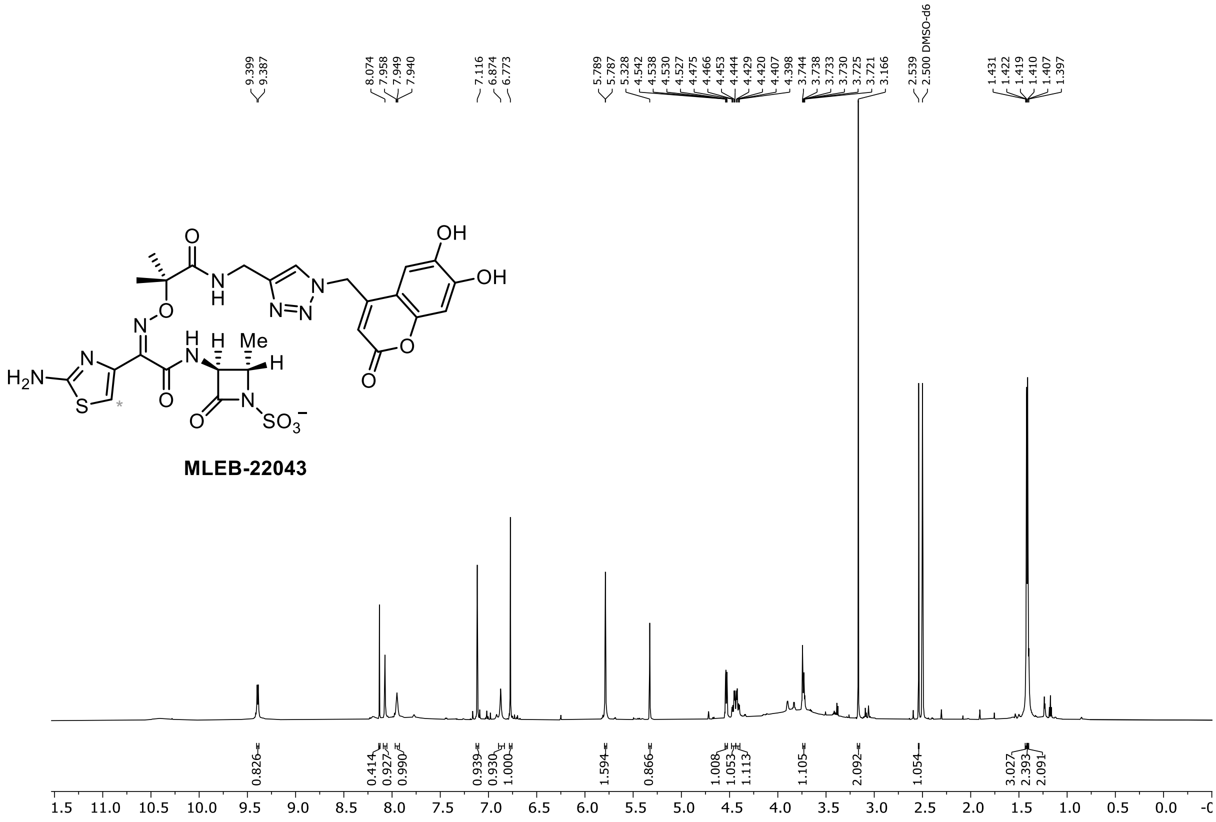

MLEB-22043

References

(1) *M100Ed33 | Performance Standards for Antimicrobial Susceptibility Testing, 33rd Edition*. Clinical & Laboratory Standards Institute. https://clsi.org/standards/products/microbiology/documents/m100/ (accessed 2023-05-28).

(2) Alcock, B. P.; Huynh, W.; Chalil, R.; Smith, K. W.; Raphenya, A. R.; Wlodarski, M. A.; Edalatmand, A.; Petkau, A.; Syed, S. A.; Tsang, K. K.; Baker, S. J. C.; Dave, M.; McCarthy, M. C.; Mukiri, K. M.; Nasir, J. A.; Golbon, B.; Imtiaz, H.; Jiang, X.; Kaur, K.; Kwong, M.; Liang, Z. C.; Niu, K. C.; Shan, P.; Yang, J. Y. J.; Gray, K. L.; Hoad, G. R.; Jia, B.; Bhando, T.; Carfrae, L. A.; Farha, M. A.; French, S.; Gordzevich, R.; Rachwalski, K.; Tu, M. M.; Bordeleau, E.; Dooley, D.; Griffiths, E.; Zubyk, H. L.; Brown, E. D.; Maguire, F.; Beiko, R. G.; Hsiao, W. W. L.; Brinkman, F. S. L.; Van Domselaar, G.; McArthur, A. G. CARD 2023: Expanded Curation, Support for Machine Learning, and Resistome Prediction at the Comprehensive Antibiotic Resistance Database. *Nucleic Acids Res* **2023**, *51* (D1), D690–D699. https://doi.org/10.1093/nar/gkac920.

(3) Ramage, B.; Erolin, R.; Held, K.; Gasper, J.; Weiss, E.; Brittnacher, M.; Gallagher, L.; Manoil, C. Comprehensive Arrayed Transposon Mutant Library of Klebsiella Pneumoniae Outbreak Strain KPNIH1. *J. Bacteriol.* **2017**, *199* (20). https://doi.org/10.1128/JB.00352-17.

(4) Snitkin, E. S.; Zelazny, A. M.; Thomas, P. J.; Stock, F.; NISC Comparative Sequencing Program Group; Henderson, D. K.; Palmore, T. N.; Segre, J. A. Tracking a Hospital Outbreak of Carbapenem-Resistant Klebsiella Pneumoniae with Whole-Genome Sequencing. *Sci Transl Med* **2012**, *4* (148), 148ra116. https://doi.org/10.1126/scitranslmed.3004129.

(5) Baba, T.; Ara, T.; Hasegawa, M.; Takai, Y.; Okumura, Y.; Baba, M.; Datsenko, K. A.; Tomita, M.; Wanner, B. L.; Mori, H. Construction of Escherichia Coli K-12 in-Frame, Single-Gene Knockout Mutants: The Keio Collection. *Mol Syst Biol* **2006**, *2*, 2006.0008. https://doi.org/10.1038/msb4100050.

(6) Liberati, N. T.; Urbach, J. M.; Miyata, S.; Lee, D. G.; Drenkard, E.; Wu, G.; Villanueva, J.; Wei, T.; Ausubel, F. M. An Ordered, Nonredundant Library of Pseudomonas Aeruginosa Strain PA14 Transposon Insertion Mutants. *Proc Natl Acad Sci U S A* **2006**, *103* (8), 2833–2838. https://doi.org/10.1073/pnas.0511100103.

(7) Kim, A.; Kutschke, A.; Ehmann, D. E.; Patey, S. A.; Crandon, J. L.; Gorseth, E.; Miller, A. A.; McLaughlin, R. E.; Blinn, C. M.; Chen, A.; Nayar, A. S.; Dangel, B.; Tsai, A. S.; Rooney, M. T.; Murphy-Benenato, K. E.; Eakin, A. E.; Nicolau, D. P. Pharmacodynamic Profiling of a Siderophore-Conjugated Monocarbam in Pseudomonas Aeruginosa: Assessing the Risk for Resistance and Attenuated Efficacy. *Antimicrob Agents Chemother* **2015**, *59* (12), 7743–7752. https://doi.org/10.1128/AAC.00831-15.

(8) Jacobs, M. A.; Alwood, A.; Thaipisuttikul, I.; Spencer, D.; Haugen, E.; Ernst, S.; Will, O.; Kaul, R.; Raymond, C.; Levy, R.; Chun-Rong, L.; Guenthner, D.; Bovee, D.; Olson, M. V.; Manoil, C. Comprehensive Transposon Mutant Library of Pseudomonas Aeruginosa. *Proc Natl Acad Sci U S A* **2003**, *100* (24), 14339–14344. https://doi.org/10.1073/pnas.2036282100.

(9) Ranieri, M. R. M.; Chan, D. C. K.; Yaeger, L. N.; Rudolph, M.; Karabelas-Pittman, S.; Abdo, H.; Chee, J.; Harvey, H.; Nguyen, U.; Burrows, L. L. Thiostrepton Hijacks Pyoverdine Receptors To Inhibit Growth of Pseudomonas Aeruginosa. *Antimicrob Agents Chemother* **2019**, *63* (9), e00472-19. https://doi.org/10.1128/AAC.00472-19.

(10) Cox, G.; Sieron, A.; King, A. M.; De Pascale, G.; Pawlowski, A. C.; Koteva, K.; Wright, G. D. A Common Platform for Antibiotic Dereplication and Adjuvant Discovery. *Cell Chem Biol* **2017**, *24* (1), 98–109. https://doi.org/10.1016/j.chembiol.2016.11.011.

(11) Gallagher, L. A.; Ramage, E.; Weiss, E. J.; Radey, M.; Hayden, H. S.; Held, K. G.; Huse, H. K.; Zurawski, D. V.; Brittnacher, M. J.; Manoil, C. Resources for Genetic and Genomic Analysis of Emerging Pathogen Acinetobacter Baumannii. *J Bacteriol* **2015**, *197* (12), 2027–2035. https://doi.org/10.1128/JB.00131-15.
